# Mechanisms used for cDNA synthesis and site-specific integration of RNA into DNA genomes by a reverse transcriptase–Cas1 fusion protein

**DOI:** 10.1126/sciadv.adk8791

**Published:** 2024-04-12

**Authors:** Georg Mohr, Jun Yao, Seung Kuk Park, Laura Markham, Alan M. Lambowitz

**Affiliations:** Departments of Molecular Biosciences and Oncology, University of Texas at Austin, Austin, TX 78712, USA.

## Abstract

Reverse transcriptase–Cas1 (RT-Cas1) fusion proteins found in some CRISPR systems enable spacer acquisition from both RNA and DNA, but the mechanism of RNA spacer acquisition has remained unclear. Here, we found that *Marinomonas mediterranea* RT-Cas1/Cas2 adds short 3′-DNA (dN) tails to RNA protospacers, enabling their direct integration into CRISPR arrays as 3′-dN-RNAs or 3′-dN-RNA/cDNA duplexes at rates comparable to similarly configured DNAs. Reverse transcription of RNA protospacers is initiated at 3′ proximal sites by multiple mechanisms, including recently described de novo initiation, protein priming with any dNTP, and use of short exogenous or synthesized DNA oligomer primers, enabling synthesis of near full-length cDNAs of diverse RNAs without fixed sequence requirements. The integration of 3′-dN-RNAs or single-stranded DNAs (ssDNAs) is favored over duplexes at higher protospacer concentrations, potentially relevant to spacer acquisition from abundant pathogen RNAs or ssDNA fragments generated by phage defense nucleases. Our findings reveal mechanisms for site-specifically integrating RNA into DNA genomes with potential biotechnological applications.

## Teaser

A promiscuous reverse transcriptase fused to a DNA integrase promotes site-specific integration of RNA into DNA genomes.

## INTRODUCTION

Bacteria face incessant attacks by viruses and genomic parasites and have evolved defense systems to combat these threats. Among the most widely studied are CRISPR-Cas systems found in diverse bacteria and archaea (*1*). CRISPR-Cas systems typically include an RNA-guided nuclease (effector) complex, a CRISPR repeat locus that accepts snippets of nucleic acids (protospacers) derived from threats, enzymes (Cas4, Cas5, or Cas6) that process transcribed spacers into RNA guides for nuclease effector complexes, and a DNA integrase (Cas1/Cas2) that site-specifically integrates new spacers from invading pathogens into CRISPR arrays (*2*–*8*). Six types of CRISPR systems (types I to VI) each with multiple subclasses have been distinguished (*1*). Type III systems differ from the others in their ability to cleave both DNA and RNA in a transcriptionally coupled reaction (*9*–*11*). In addition, some type III CRISPR systems have an associated reverse transcriptase (RT), either coexpressed with Cas1/Cas2 or present as an RT-Cas1 fusion protein, some of which also have an N-terminal Cas6 domain that processes guide RNAs for incorporation into effector complexes (*12*–*16*). Most CRISPR-associated RTs are closely related to the RTs encoded by mobile group II introns, prolific bacterial retrotransposons whose dissociated RTs have evolved in different bacteria to perform a variety of cellular functions (*17*–*22*). Four RT-Cas1 proteins have been shown to site-specifically integrate RNA as well as DNA into CRISPR arrays in vivo (*12*, *23*–*25*), but the mechanisms by which RNA protospacers are reverse-transcribed and integrated into CRISPR arrays have remained unclear.

Although the six types of CRISPR systems vary in the mechanisms used for processing guide RNAs and destroying invading nucleic acids, all use the same Cas1/Cas2-mediated cleavage-ligation (transesterification) mechanism to site-specifically integrate double-stranded DNA (dsDNA) protospacers into CRISPR arrays (*26*–*30*). Structural studies have shown that Cas1 and Cas2 form a hexameric complex that binds a dsDNA protospacer across its length with overhanging or splayed single-stranded 3′ ends of opposite strands inserted in one or both active sites of Cas1 proteins on opposite sides of the complex (*25*, *31*–*37*). Cas1/Cas2 complexes from different organisms have structural variations that favor different length spacers and splay open different length single-stranded 3′ ends (*30*, *38*). A single-molecule study of spacer acquisition by an *Enterococcus faecalis* type II-A CRISPR system indicated that the Cas1/Cas2 complex remains stably bound to the integrated spacer until it is dislodged by transcription-coupled DNA repair, which fills in and seals single-stranded gaps to fully integrate the newly acquired spacer into the host genome (*39*). This process enables integration of protospacers into CRISPR arrays without introducing deleterious double-strand breaks in bacterial chromosomal DNA.

**Fig. 1.**
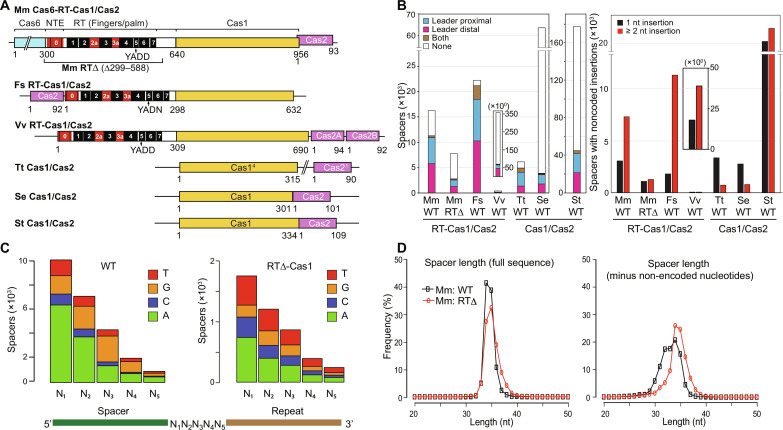
Spacers acquired by RT-Cas1/Cas2 proteins have higher numbers of noncoded nucleotides at spacer-repeat junctions than those acquired by Cas1/Cas2 proteins lacking an RT domain. (**A**) Schematics of RT-Cas1/Cas2 and Cas1/Cas2 proteins associated with type III CRISPR systems from *M. mediterranea* (Mm), *F. saccharivorans* (Fs), *V. vulnificus* (Vv), *T. thermophilus* (Tt), *S. epidermidis* (Se), and *S. thermophilus* (St). RT1 to S7 (black), conserved sequence blocks present in all RTs; NTE/RT0, RT2a, and RT3a (red), structurally conserved regions of non-LTR retroelement RTs that are absent in retroviral RTs. (**B**) Analysis of noncoded nucleotides associated with leader-proximal spacers acquired in vivo by different RT-Cas1/Cas2 or Cas1/Cas2 proteins. Left, numbers of spacers with noncoded nucleotides at their leader-proximal end only (blue), leader-distal end only (red), both ends (brown), or neither end (none, white). Right, numbers of spacers with a single (black) or ≥2 noncoded nucleotides (red), excluding small numbers of spacers with noncoded nucleotides at both spacer-repeat junctions. The analysis was done using datasets listed in Materials and Methods for unique spacer sequences mapped to the host or phage genomes (table S4). The inset bar graphs for the smaller number of Vv RT-Cas1/Cas2 spacers are plotted on a different scale (×10^0^). (**C**) Stacked bar graphs comparing the number of spacers acquired by Mm WT RT-Cas1/Cas2 or RTΔ-Cas1/Cas2 with A, C, G, or T residues at noncoded nucleotide positions N_1_ to N_5_. The analysis was done for spacers that mapped to the *M. mediterranea* strain MMB1 genome and had noncoded nucleotides at one end only, putatively the 3′ end used for terminal transferase addition of noncoded nucleotides. (**D**) Length distribution of unique spacer sequences acquired by Mm WT RT-Cas1 (black) or RTΔ-Cas1 (red). Left, length distribution of spacers defined as the sequence between two repeats; right, length distribution of the same spacers after removing noncoded nucleotides.

Biochemical analyses of the mechanism by which RT-Cas1 proteins acquire spacers from RNA have been sparse. The four RT-Cas1 fusion proteins that acquire spacers from RNA are composed of an RT domain corresponding to the fingers and palm of retroviral RTs but fused directly to Cas1 rather than a canonical thumb domain as in other RTs (Fig. 1A). The RT domain of RT-Cas1 proteins contains seven conserved sequence blocks (RT1 to RT7) found in all RTs plus an N-terminal extension with an RT0 loop and two expanded regions (RT2a and RT3a) between the conserved RT sequence blocks (Fig. 1A). These additional regions are absent in retroviral RTs, but structurally conserved and functionally important in group II intron and other bacterial RTs, as well as in LINE-1 and other eukaryotic non–long terminal repeat (LTR) retrotransposon RTs (collectively termed non-LTR retroelement RTs) (*40*). The *Marinomonas mediterranea* (Mm) RT-Cas1 protein, associated with a type III-A CRISPR system, was shown to function in complex with Cas2 to site-specifically integrate DNA and RNA protospacers into CRISPR arrays in vivo and in vitro (*12*). It was also shown to have an active N-terminal Cas6 domain that functions in CRISPR RNA processing and whose interaction with the RT domain is required for RT activity (*14*). A cryo–electron microscopy structure showed that a closely related *Thiomicrospira* type III Cas6-RT-Cas1/Cas2 forms a hexameric complex similar to Cas1/Cas2 proteins that acquire spacers from DNA, but with the Cas6 and RT domains interacting with each other to form separate flexibly attached lobes and structural differences in regions of Cas1/Cas2 that function in protospacer binding (*14*, *25*). The *Fusicatenibacter saccharivorans* (Fs) and *Vibrio vulnificus* (Vv) RT-Cas1 proteins, both associated with type III-D CRISPR systems, were shown to acquire RNA-derived spacers in vivo but have not been investigated biochemically (*23*, *24*). Mm RT-Cas1/Cas2 could acquire both RNA and DNA protospacers in its native host, but only DNA protospacers in *Escherichia coli*, suggesting that host-specific factors contribute to RNA protospacer acquisition in vivo (*12*).

Here, we focused on mechanisms used for reverse transcription and RNA protospacer integration by Mm RT-Cas1/Cas2. Zabrady *et al.* (*41*) recently reported that Mm RT-Cas1/Cas2 could initiate reverse transcription de novo at C residues by using a Mn^2+^-dependent primase activity with a strong preference for initiating at CC sequences. Here, we found that Mm RT-Cas1/Cas2 could also initiate reverse transcription at 3′-proximal sites by protein priming with any deoxynucleotide triphosphate (dNTP) and by using short exogenous and likely synthesized DNA oligonucleotide primers with no fixed sequence requirements, as desired for an enzyme whose biological function is to acquire spacers from diverse RNAs. Zabrady *et al.* (*41*) also reported that Mm RT-Cas1/Cas2 could add short 3′-DNA (3′-dN) extensions to RNA protospacers but that the efficient integration of RNA protospacers into a CRISPR array required synthesis of a complementary DNA (cDNA) to generate an RNA/cDNA duplex that is a preferred substrate for the Cas1/Cas2 DNA integrase. Here, we found that Mm RT-Cas1/Cas2 could directly integrate single-stranded 3′-dN RNAs as well as 3′-dN-RNA/cDNA duplexes into a CRISPR array at rates comparable to similarly configured DNA protospacers with bioinformatic analysis, showing that spacers acquired from 3′-dN–tailed RNAs comprised a high proportion of those acquired by RT-Cas1/Cas2 proteins in vivo.

## RESULTS

### Spacers acquired from RNA have higher numbers of noncoded nucleotides at spacer-repeat junctions than spacers acquired from DNA

We wondered whether spacers acquired from RNA might have distinctive features that could provide clues about their acquisition mechanism. To identify such features, we compared the sequences of newly acquired spacers (i.e., those closest to the leader) for three RT-Cas1 proteins (Mm, Fs, and Vv) in host strains that support spacer acquisition from RNA compared to spacers acquired from DNA by an Mm RT-Cas1 mutant lacking the RT domain (Cas6-RTΔ-Cas1, denoted RTΔ-Cas1) and Cas1 proteins from *Thermus thermophilus* (Tt), *Staphylococcus epidermidis* (Se), and *Streptococcus thermophilus* (St) type III systems that lack an associated RT (*28*, *42*, *43*).

The leader-proximal spacers acquired by these RT-Cas1/Cas2 and Cas1/Cas2 proteins included different proportions with noncoded nucleotides at one or both spacer-repeat junctions that did not correlate with their ability to acquire spacers from RNA (Fig. 1B, left). However, the number of noncoded nucleotides at the spacer-repeat junctions differed markedly for proteins that could acquire spacers from RNA compared to those that could not. Most newly acquired spacers for the wild-type (WT) Mm, Fs, and Vv RT-Cas1 proteins, which acquire spacers from RNA, had two or more noncoded nucleotides at the spacer-repeat junctions, while deletion of the RT domain of Mm RTΔ-Cas1 sharply decreased the proportion of spacers that had ≥2 noncoded nucleotides, approaching those for the Tt, Se, and St Cas1/Cas2 proteins, which acquire spacers from DNA (Fig. 1B, right). For WT Mm RT-Cas1, the first two noncoded nucleotides at the spacer-repeat junction of spacers that had noncoded nucleotides at one end only, putatively the 3′ end, were predominantly A residues (>50%) followed by G > T > C residues, while spacers acquired by Mm RTΔ-Cas1/Cas2 showed less bias for A residues and had higher proportions of C and T residues at all positions (Fig. 1C; confirmed by analyzing noncoded nucleotides at the 3′ end of RNA sense-strand sequences in the top and bottom strands of the CRISPR array; fig. S1A). The length distribution of spacers acquired in vivo by Mm WT RT-Cas1/Cas2 and RTΔ-Cas1/Cas2 was similar with peaks at 34 to 35 nucleotides (nt) (Fig. 1D, left). However, spacers acquired by WT RT-Cas1/Cas2 required addition of one to six noncoded nucleotides to achieve a length distribution similar to that of spacers acquired from DNA by RTΔ-Cas1 (Fig. 1D, right). Collectively these findings suggested that Mm RT-Cas1 might have a terminal transferase activity that adds noncoded nucleotides, preferentially A residues, to the 3′ ends of RNA protospacers.

**Fig. 2.**
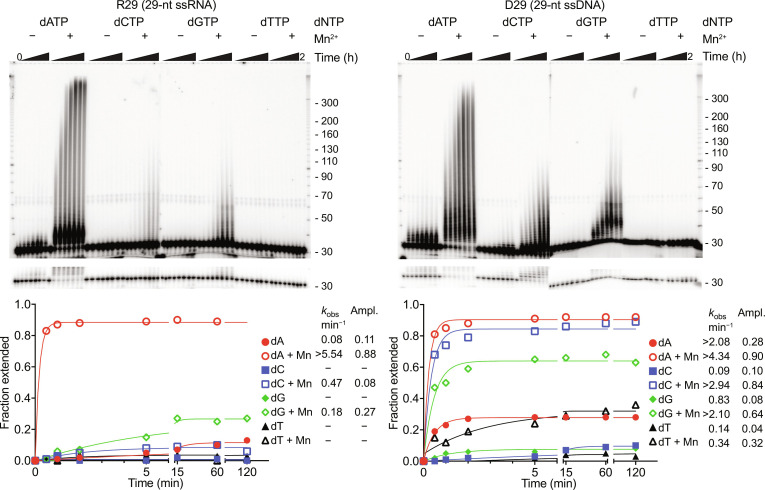
Nucleotide preferences for addition of noncoded deoxynucleotides to the 3′ end of RNA or DNA oligonucleotide protospacers by Mm RT-Cas1/Cas2 terminal transferase activity. Terminal transferase assays were done as described in Materials and Methods by incubating purified Mm WT RT-Cas1/Cas2 with 5′ ^32^P-labeled 29-nt RNA (R29, left) or DNA (D29, right) oligonucleotides having the same nucleotide sequence and 1 mM of the indicated dNTP in reaction medium containing 10 mM MgCl_2_ without (−) or with (+) 1 mM MnCl_2_. After incubating at 37°C for times up to 2 hours, the products were analyzed in a denaturing 6% polyacrylamide gel, which was dried and quantified with a phosphorimager. Lighter exposures showing resolution of single-nucleotide extensions are appended below the bottom of gel. The hatch marks to the right of the gel indicate the positions of 5′-labeled size markers (10-nt ssDNA ladder; Invitrogen) in a parallel lane. The plots show the fraction of labeled product as a function of time with the data fit to a single exponential equation to obtain values for *k*_obs_ and amplitude. *R*^2^ values for curve fits are listed in table S2. A dash indicates no detectable extension confirmed by the lighter exposure of the bottom part of the gel. A repeat of the terminal transferase assays with 5′-labeled oligonucleotides and unlabeled dNTPs incubated for 1 hour at 37°C showed similar nucleotide preferences (fig. S8).

Prompted by these findings, we assayed terminal transferase activity of WT Mm RT-Cas1/Cas2 with 29-nt RNA and DNA oligonucleotide substrates (R29 and D29, respectively) in reaction medium containing 10 mM Mg^2+^ in the absence or presence of 1 mM Mn^2+^, a physiologically relevant divalent cation that modulates the activity of many polymerases (*44*, *45*). Consistent with the in vivo findings, the assays showed that WT RT-Cas1/Cas2 has a terminal transferase activity that adds noncoded DNA tails to the 3′ end of RNA and DNA substrates with nucleotide preferences A >> G > C > T for the RNA substrate and A > C > G > T for the DNA substrate and Mn^2+^ strongly increasing both the activity and preference for adding A residues to the 3′ end of the RNA substrate (Fig. 2). Additional terminal transferase assays with RNA and DNA oligonucleotide substrates having a different sequence and 3′ nucleotide showed a strong bias for purines over pyrimidines but little or no bias for dA over dG residues in the presence of Mn^2+^ (figs. S1B and S8). Notably, the Mm RT-Cas1/Cas2 terminal transferase had a propensity to stop or pause after addition of short (~4 nt) dN tails, particularly evident for addition of dA tails to the RNA substrate (Fig. 2 and fig. S1B), possibly a feature that helps keep overall protospacer length in a range that can be accommodated by Cas1/Cas2. The greater preference of Mm RT-Cas1/Cas2 for adding A residues to the 3′ end of the RNA than the DNA substrate explains why spacers acquired by Mm RT-Cas1/Cas2 in vivo have a higher proportion of A residues at RNA proximal positions 1 and 2, but lower proportions of A residues at more distal positions 3 to 5, where nucleotide addition occurs to progressively longer stretches of DNA resulting from prior dNTP additions. Further analysis showed that spacers acquired in vivo by WT Mm RT-Cas1/Cas2 had significantly higher frequencies of noncoded AA, AG, and GG dinucleotides at noncoded nucleotide positions 1 and 2 than those acquired by Mm RTΔ-Cas1/Cas2 (fig. S1C). Despite differences in the numbers of noncoded nucleotides, the spacers acquired by both proteins were uniformly distributed throughout the gene from which they were derived (fig. S1D). Considered together, these findings suggested that addition of deoxynucleotides to the 3′ end of RNA fragment protospacers by RT-Cas1/Cas2 terminal transferase activity might be required for RNA protospacer acquisition.

### Addition of small numbers of deoxynucleotides to the 3′ end of RNA protospacers is necessary and sufficient for RNA spacer acquisition

**Fig. 3.**
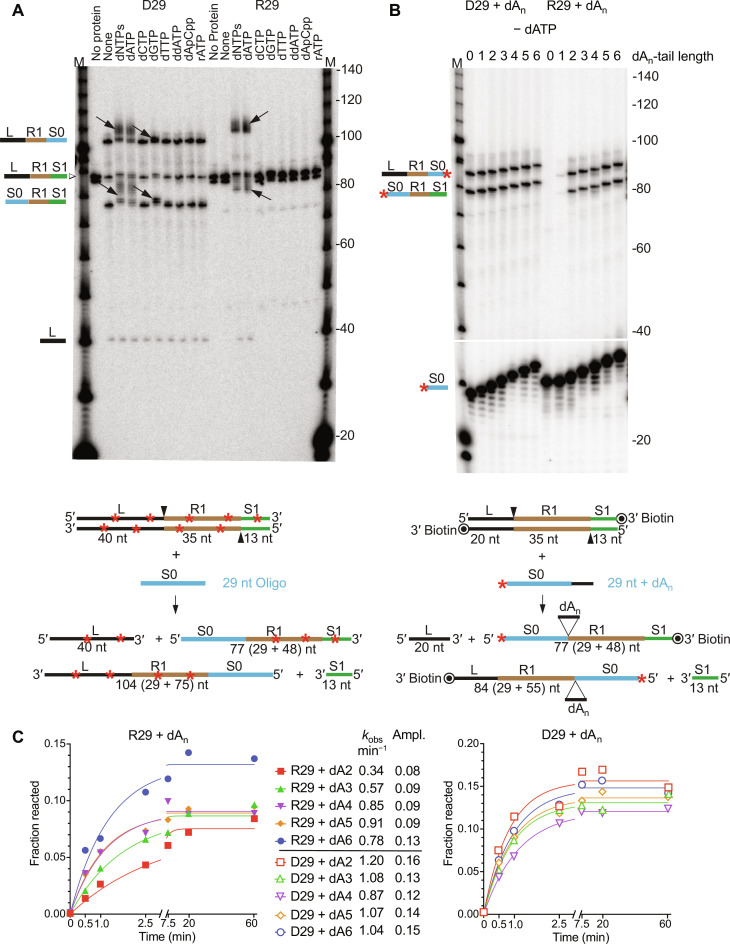
RNA protospacer integration into a CRISPR array by Mm RT-Cas1/Cas2 requires 3′ deoxynucleotides. (**A**) Mm WT RT-Cas1/Cas2 was incubated with 29-nt DNA or RNA protospacers having the same sequence (D29 and R29, respectively, S0) and an 88-bp internally ^32^P-labeled (red stars) CRISPR DNA in the presence or absence (None) of the indicated dNTPs for 1 hour at 37°C. The products were analyzed in a 6% polyacrylamide/urea gel, which was dried and scanned with a phosphorimager. Lanes: No protein, labeled CRISPR DNA incubated without RT-Cas1/Cas2 or dNTPs; M, 5′-labeled 10-nt DNA ladder; arrows, integration products whose lengths increased in the presence of dNTPs. The labeled CRISPR DNA strands ran as closely spaced doublets. A repeat of the experiment using a different CRISPR DNA gave similar results (fig. S9). (**B**) Mm WT RT-Cas1/Cas2 was incubated for 1 hour at 37°C with 5′ ^32^P-labeled (red asterisk) D29 and R29 oligonucleotide protospacers without or with different length 3′-dA tails and a 3′-blocked unlabeled 68-bp CRISPR DNA. Products were analyzed in a 6% polyacrylamide/urea gel. The gap in the phosphorimager scan demarcates a lighter exposure of the bottom of the gel. (**C**) Time courses for integration of DNA and RNA protospacers with different length 3′-dA tails (denoted dA_n_). Reactions were done with 5′-labeled protospacers as in (B) for times up to 1 hour. The plots show the fraction of labeled oligonucleotide inserted into the top and bottom strands of the CRISPR DNA as a function of time fit to a single exponential equation to obtain values for *k*_obs_ and amplitude. *R*^2^ values for curve fits are listed in table S2. The gels for the time courses in (C) and a full repeat of the experiment are shown in fig. S2. Gels with time courses for dA1 protospacers are shown in fig. S10.

To investigate whether addition of 3′-dN tails is required for RNA spacer acquisition, we performed spacer acquisition assays with Mm WT RT-Cas1/Cas2 in the presence or absence of dNTPs. In an initial assay, we incubated Mm RT-Cas1/Cas2 with an internally ^32^P-labeled double-stranded CRISPR DNA and 29-nt single-stranded DNA (ssDNA) (D29) or RNA (R29) protospacers having the same nucleotide sequence in the presence or absence of each of the four dNTPs, dideoxyadenosine triphosphate (ddATP), a nonhydrolyzable dATP analog (dApCpp), or ATP. The reactions were done in the absence of Mn^2+^ to limit terminal transferase addition of noncoded nucleotides to the 3′ ends of the labeled CRISPR DNA, and the products were analyzed in a denaturing 6% polyacrylamide gel. Spacer ligation to the 5′ end of the first repeat (R1) on opposite strands was expected to occur via transesterification reactions that yield labeled top-strand products corresponding to the cleaved leader (L, 40 nt) and the 29-nt protospacer (S0) linked to the 5′ end of R1 (S0 + R1 + S1, 77 nt) and labeled bottom-strand products corresponding to L + R1 + S0 (104 nt) plus unlabeled S1 (13 nt, run off the gel; schematic Fig. 3A).

The results showed that Mm RT-Cas1/Cas2 ligates DNA protospacers to the 5′ end of R1 on both strands in the presence or absence of added dNTPs, as expected, while efficient RNA protospacer ligation occurred only in the presence of dNTPs or dATP (Fig. 3A), the nucleotide used most efficiently by Mm RT-Cas1/Cas2 terminal transferase activity in the absence of Mn^2+^ (Fig. 2). The DNA protospacers integrated in the presence of dNTPs, dATP, or dGTP (deoxyguanosine triphosphate) and the RNA protospacers integrated in the presence of dNTPs or dATP were slightly longer than the initial protospacers as expected for terminal transferase addition of deoxynucleotide tails before integration (Fig. 3A, arrows in gel). The finding that dATP was by itself sufficient for RNA protospacer integration in the absence of other dNTPs indicates that RNA protospacer acquisition required only the addition of a 3′-dA tail to an RNA protospacer and not cDNA synthesis to generate a complementary cDNA strand, in agreement with a previous finding (*12*).

To investigate how many noncoded 3′ deoxynucleotides are required for RNA spacer acquisition, we performed spacer acquisition assays with the same 29-nt DNA and RNA oligonucleotides without or with one to six dA residues added to their 3′ ends (Fig. 3B). The oligonucleotides were 5′-^32^P–labeled (denoted by red *) and used to assay spacer ligation into an unlabeled CRISPR DNA in the absence of added dNTPs or dATP. The oligonucleotides comprising the CRISPR array had a 3′ biotin-blocking group to prevent terminal transferase addition of dNTPs to the 3′ DNA ends.

As expected, the 5′-labeled ssDNA oligonucleotides with or without a 3′-dA tail were efficiently ligated into the CRISPR DNA and produced the two expected ligation products (S0 + R1 + L, 77 nt and L + R1 + S0, 84 nt), whose lengths increased progressively with increasing length of the dA tail (Fig. 3B, left side). By contrast, the 29-nt RNA oligonucleotide without a 3′-dA residue was not integrated into the CRISPR DNA and the RNA oligonucleotide with a single 3′-dA residue was integrated inefficiently, whereas RNA oligonucleotides with two or more dA residues at their 3′ ends were used more efficiently as protospacers (Fig. 3B, right side).

Time courses comparing rates of integration (*k*_obs_) of the 5′-labeled 29-nt DNA or RNA protospacers with dA tails ranging in length from 2 to 6 nt showed relatively small differences for DNA protospacers but greater dependence on dA-tail length for RNA protospacers, with RNA protospacers having 4- to 6-nt dA tails integrating at rates close to those for DNA protospacers of the same length (Fig. 3C and fig. S2, A and B). Addition of dATP to integration reactions with the 5′-labeled R29 and D29 oligonucleotides that had different length 3′-dA tails showed that optimal integration of shorter RNA but not DNA protospacers required extension to 33 nt (fig. S2C), matching the length distribution of spacers acquired by Mm WT RT-Cas1/Cas2 in vivo (Fig. 1D). Collectively, these findings showed that addition of short 3′-dN tails by Mm RT-Cas1/Cas2 terminal transferase activity enabled relatively efficient integration of RNA protospacers into a CRISPR array without a requirement for synthesis of a cDNA.

### RT-Cas1/Cas2 synthesizes near full-length DNA copies of 50-nt RNA or DNA templates without an added primer

**Fig. 4.**
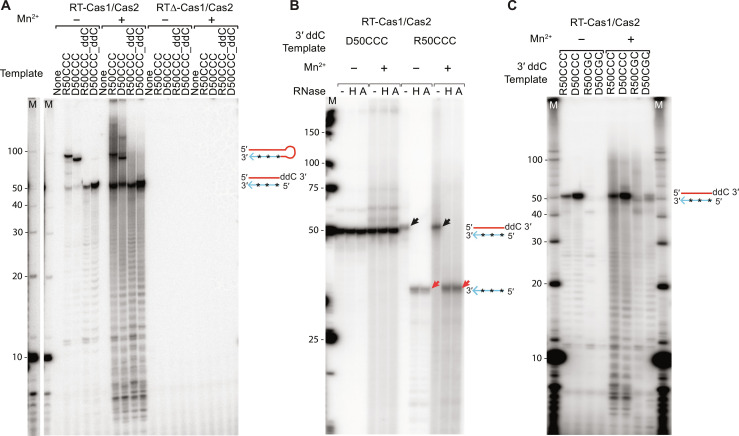
RT-Cas1/Cas2 synthesizes near full-length copies of RNA and DNA templates by initiating at 3′-proximal sites without an added primer. (**A**) DNA synthesis reactions were done by incubating WT RT-Cas1/Cas2 (left) or RT∆-Cas1/Cas2 (right) with 50-nt RNA or DNA oligonucleotide templates (250 nM) containing a 3′-proximal CCC sequence (R50CCC and D50CCC, respectively) without or with a 3′-ddC blocking group and ^32^P-labeled dNTPs (20 μM [α-^32^P]-dCTP + 500 μM dATP, dGTP, and dTTP) in reaction medium containing 10 mM MgCl_2_ ± 1 mM MnCl_2_ for 1 hour at 25°C. After phenol extraction, the samples were analyzed in a denaturing 20% polyacrylamide gel, which was dried and scanned with a phosphorimager. Lane M, 5′-labeled 10-nt ssDNA ladder size markers in a parallel lane, with a darker exposure of that lane appended on the left. (**B**) RT-Cas1/Cas2 cDNA synthesis reactions done as in (A) with 3′-blocked 50-nt DNA and RNA templates containing a 3′ proximal CCC sequence. After cleanup with a Zymo Oligo Clean and Concentrator kit, the products were incubated for 20 min at 37°C in the absence or presence of RNase H or RNase A, followed by protease K digestion and analysis in a nondenaturing 15% polyacrylamide gel. Lane M, 5′-labeled low molecular DNA ladder size markers (New England Biolabs). (**C**) RT-Cas1/Cas2 DNA synthesis reactions with 3′-blocked DNA and RNA templates containing a 3′ proximal CCC or CGC sequence. Reactions were done and analyzed in a denaturing 20% polyacrylamide gel as in (A). The schematics to the right of the gels in (A) to (C) depict the labeled cDNAs (blue line with stars) synthesized from RNA or DNA templates (red). All experiments shown in the figure were repeated with similar results (fig. S11).

**Fig. 5.**
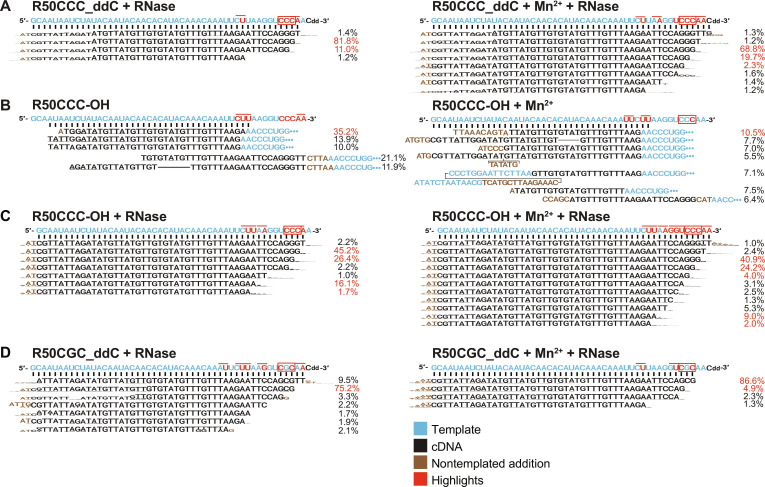
TGIRT-seq of cDNAs synthesized by Mm RT-Cas1/Cas2 from RNA templates containing a 3′-proximal CCC or CGC sequence. (**A**) R50CCC_ddC. (**B** and **C**) R50CCC-OH. (**D**) R50CGC_ddC. Reverse transcription was done by incubating Mm RT-Cas1/Cas2 (500 nM) with RNA template (250 nM) and an equimolar mix of 500 μM dATP, dCTP, dGTP, and dTTP in reaction medium containing 10 mM MgCl_2_ without (left) or with (right) 1 mM MnCl_2_ for 1 hour at 25°C. After stopping the reactions by adding 25 mM EDTA, products were incubated without or with RNase A, as indicated in the figure, followed by protease K digestion, cleanup, and construction of TGIRT-seq libraries, as described in Materials and Methods. The libraries were sequenced on an Illumina NextSeq 550 to obtain ~1 million reads for each sample. For each sample, the RNA template sequence (blue letters with red highlights) is shown above with prevalent reads (>1% for cDNAs synthesized from 3′-blocked RNA templates and >5% for RNA templates with a 3′ OH) shown below in sequence logo format with the height of the letter corresponding to the proportion of the nucleotide at that position. Noncoded nucleotides are colored brown, and 3′-RNA template sequences used to prime snap-back cDNA synthesis are colored blue, with blue dots indicating an extended not shown RNA template sequence. Nucleotides in the RNA template corresponding to first nucleotide of a cDNA product are highlighted in red, with a thin red line above the template extending over the first two nucleotides of the synthesized cDNA. The 3′-proximal CCC and mutant CGC sequence in the template are highlighted in red. cDNA start sites identified by sequencing were consistent with gel analysis of ^32^P-labeled cDNAs.

To investigate how Mm RT-Cas1/Cas2 synthesizes DNA copies of RNAs or ssDNAs, we began by using 50-nt RNA or DNA oligonucleotide templates of the same sequence that fortuitously included a 3′-proximal CCC sequence that turned out to be a preferred cDNA initiation site for Mm RT-Cas1/Cas2 (templates denoted R50CCC and D50CCC, respectively; Figs. 4 and 5). The RNA and DNA templates were tested without or with a 3′-ddC blocking group, which prevents 3′-deoxynucleotide addition by RT-Cas1 terminal transferase activity as well as “snap-back DNA synthesis,” a reaction in which the 3′ end of a DNA or RNA template folds back to prime DNA synthesis by base pairing to short complementary sequences upstream in the same template (*46*). For initial experiments, DNA synthesis reactions were done by incubating the R50CCC and D50CCC templates with Mm WT RT-Cas1/Cas2 and ^32^P-dNTPs [a mixture of 20 μM [α-^32^P]-dCTP (deoxycytidine triphosphate) + 500 μM dATP, dGTP, and dTTP (deoxythymidine triphosphate)] in reaction medium containing 10 mM Mg^2+^ in the absence or presence of 1 mM Mn^2+^, and the products were analyzed in a denaturing 20% polyacrylamide gel.

In the absence of a 3′-blocking group, RT-Cas1/Cas2 gave two major products with both the RNA and DNA template, one of ~100 nt, the size expected for a snap-back DNA synthesis product, and the other of ~50 nt, whose synthesis was stimulated by Mn^2+^ and corresponded to a near full-length DNA copy of the RNA or DNA template beginning near its 3′ end (Fig. 4A). As expected, the ~50-nt DNA product but not the snap-back DNA synthesis product was seen with the 3′-blocked R50CCC_ddC and D50CCC_ddC templates, and neither product was seen with Mm RTΔ-Cas1/Cas2, which lacks RT and DNA polymerase activity (Fig. 4A). Analysis of the products on a nondenaturing polyacrylamide gel showed that incubation with ribonuclease (RNase) H or RNase A (lanes labeled H and A, respectively) had no effect on the electrophoretic mobility of the dsDNA resulting from copying of the DNA template, but increased the mobility of the product synthesized from the RNA template, indicating that it was a stable RNA-cDNA heteroduplex (Fig. 4B).

To identify cDNA start sites, we sequenced the cDNAs synthesized from the 50-nt RNA template with or without a 3′-blocking group by using a thermostable group II intron reverse transcriptase (TGIRT)–based DNA sequencing method (Fig. 5). The cDNA synthesis reactions were done with 500 μM of all four unlabeled dNTPs in the absence or presence of Mn^2+^. The sequences showed that >90% of the products synthesized from the 3′-blocked R50CCC_ddC template in the absence or presence of Mn^2+^ were near full-length cDNAs that began at the CCC sequence near the 3′ end of the RNA (Fig. 5A; cDNA initiation sites highlighted in red letters; 3′ CCC sequence highlighted in a red box). The remaining products began at 3′-proximal A or U residues, with the number of initiation sites increasing in the presence of Mn^2+^ (Fig. 5A). Most but not all of the cDNAs extended to the 5′ end of the 50-nt RNA template (indicated by proportionately smaller letters in WebLogo format) and ended with noncoded TA residues. The latter reflect nontemplated nucleotide addition by RT-Cas1/Cas2 upon reaching the 3′ end of the template, an activity found for group II intron RTs, as well as other RTs and DNA polymerases (*47*, *48*).

To test whether the lack of a 3′ hydroxyl (OH) in a 3′-blocked RNA template affects the use of cDNA initiation sites, we carried out DNA synthesis reactions with Mm RT-Cas1/Cas2 and the R50CCC template without a 3′-blocking group (R50CCC_OH) and sequenced the products without or with treatment with RNase A to degrade 3′ segments of the RNA template used to prime snap-back DNA synthesis (Fig. 5, B and C, respectively). Without RNase treatment, the major snap-back DNA synthesis initiation site identified as beginning with a sequence corresponding to the 3′ end of the RNA template (blue) was a 3′-proximal UU complementary to the 3′ terminal AA of the RNA template (Fig. 5B). Unexpectedly, snap-back DNA synthesis initiation sites also included the 3′-terminal AA of the RNA template, with cDNAs initiated at that site preceded by four or five noncoded nucleotides (brown), likely added by RT-Cas1 terminal transferase activity to give the 3′ end of the RNA template sufficient flexibility to snap-back and anneal to the complementary nucleotides at the 3′ end of the template (Fig. 5B, left). Most of the snap-back products with attached RNA sequences terminated before reaching the 5′ end of the RNA template, possibly reflecting limited processivity of RT-Cas1/Cas2 (Fig. 5B). Sequencing of the products from the same reactions with the R50CCC-OH template after RNase treatment showed that most (~70%) of the cDNAs began at the same 3′-proximal CCC sequence found for the 3′-blocked RNA template (Fig. 5C). The remainder of the RNase-treated products began at 3′-proximal A or U residues, including a substantial proportion at the major UU snap-back DNA synthesis site upstream of the 3′ CCC (see above) with most extending to the 5′ end of the template followed by noncoded TA residues (Fig. 5C). These findings indicated that aside from enabling snap-back DNA synthesis, a 3′ OH instead of a 3′-ddC blocking group did not appreciably affect Mm RT-Cas1/Cas2 cDNA initiation sites. The ability of RT-Cas1/Cas2 to initiate at 3′-proximal sites irrespective of the 3′ moiety was further supported by gel analysis of cDNAs synthesized from the same RNA template with a 3′-phosphate or inverted dT residue 3′-blocking group (fig. S3A), a desirable characteristic enabling synthesis of near full-length cDNAs from RNA fragments generated by cellular RNases that leave different 3′ moieties.

Last, to test the requirement for a dinucleotide CC sequence, which was suggested to be a highly preferred initiation site for a Mn^2+^-dependent primase activity of Mm RT-Cas1/Cas2 (*41*), we compared the efficiency of cDNA synthesis from the 3′-blocked RNA and DNA oligonucleotide templates in which 3′-proximal CCC initiation site was changed to CGC. Gel electrophoresis of labeled cDNAs synthesized from these templates with ^32^P-labeled dNTPs (20 μM [α-^32^P]-dCTP + 500 μM dATP, dGTP, and dTTP) showed that this single-nucleotide mutation strongly decreased production of near full-length cDNAs initiated at the trinucleotide site in the absence or presence of Mn^2+^ (Fig. 4C). However, sequencing of the products synthesized from the same RNA templates with 500 μM of each unlabeled dNTP showed that the CGC sequence remained a favored cDNA initiation site (79 to 92% of sequenced products) in the absence or presence of Mn^2+^ (Fig. 5D). These findings suggested that efficient initiation of cDNA synthesis at the CGC sequence might be dependent on dCTP concentration. Confirming this dependence, gel analysis of ^32^P-labeled cDNAs synthesized from templates having different 3′-proximal trinucleotide sequences in place of CCC showed more efficient initiation at CGC as well as at GGG at the higher dCTP concentration (fig. S3B). These experiments showed that Mm RT-Cas1/Cas2 could initiate cDNA synthesis at different 3′-proximal sites in the absence or presence of Mn^2+^ without an added primer or a strict requirement for a dinucleotide CC initiation site.

### RT-Cas1/Cas2 initiates cDNA synthesis at 3′-proximal sites by protein priming with different initiating dNTPs

The finding that Mm RT-Cas1 and a variety of other RTs could synthesize cDNAs that retained a 5′-triphosphate, a key indicator of de novo initiation, with a strong preference for initiation at CC sequences was based largely on experiments using short (7 to 20 nt) RNA substrates with limited sequence diversity (*41*). De novo initiation of cDNA synthesis at the CC of a 3′-tRNA-like structure was shown previously for a mitochondrial retroplasmid RT (*49*). However, the more diverse sequences of spacers acquired by Mm RT-Cas1/Cas2 in vivo led us to consider an alternate mechanism based on findings that bacterial AbiK and Abi-P2 RTs function in abortive phage infection (abi) by using nontemplated protein priming to synthesize long “random” sequence ssDNAs that contribute to altruistic cell death (*18*, *20*). Protein priming of cDNA synthesis using an OH group of a tyrosine, threonine, or serine residue resulting in covalent attachment of labeled nucleotides to the protein is a well-characterized mechanism for the initiation of cDNA synthesis by a number of viral and cellular RTs (*50*–*52*).

**Fig. 6.**
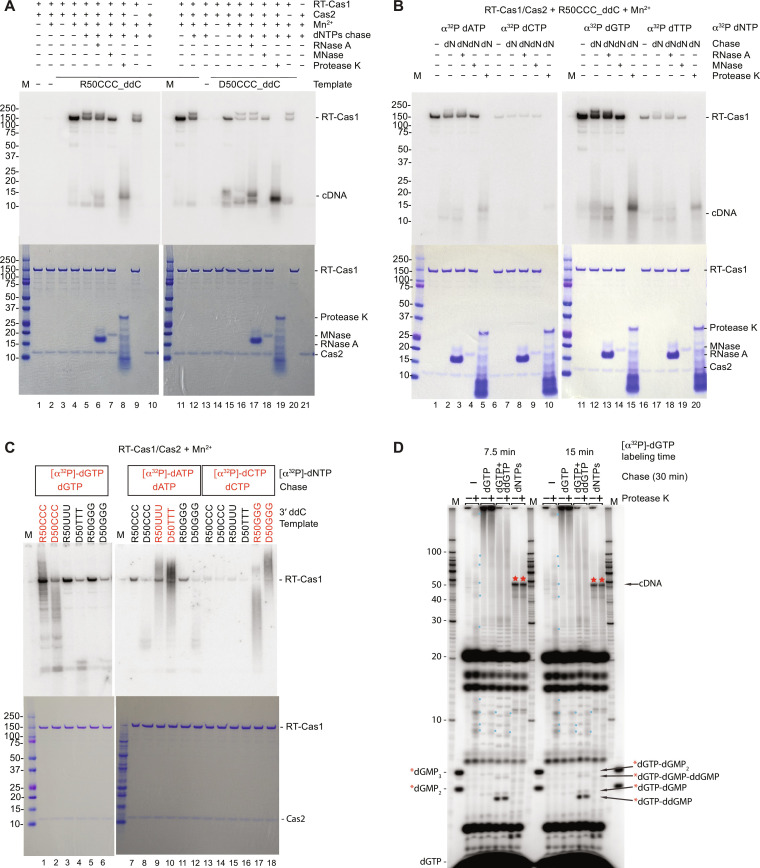
Mm RT-Cas1/Cas2 can initiate DNA synthesis at 3′-proximal sites in RNA and DNA templates by protein priming with any dNTP. (**A**) Mm RT-Cas1/Cas2, RT-Cas1, or Cas2 (500 nM) was incubated with [α-^32^P]-dGTP (5 μCi; 83 nM) without (−) or with R50CCC_ddC and D50CCC_ddC templates (250 nM) in reaction medium containing 10 mM MgCl_2_ ± 1 mM MnCl_2_ for 15 min at 25°C. Reactions were chased with unlabeled dNTPs (500 μM equimolar mix) for 30 min at 25°C and then incubated with RNase A, MNase, or protease K (15 min at 25°C), as indicated. Reactions with RNA (left) and DNA (right) templates were done and analyzed in parallel on SDS-polyacrylamide gradient gels (autoradiograms, top; Coomassie blue–stained, bottom). M, Precision Plus (Bio-Rad) size markers. (**B**) Reactions done as in (A) by incubating RT-Cas1/Cas2 with R50CCC_ddC template and different [α-^32^P]-dNTPs with 10 mM MgCl_2_ and 1 mM MnCl_2_. (**C**) Reactions done as in (B) by incubating RT-Cas1/Cas2 with 3′-blocked RNA or DNA templates with different 3′-proximal trinucleotides and [α-^32^P]-dNTPs for 15 min followed by a 30-min chase with the same dNTP (500 μM). (**D**) Reactions done as in (B) by incubating RT-Cas1/Cas2 with R50CCC_ddC template and [α-^32^P]-dGTP for 7.5 min (left) or 15 min (right), followed by a 30-min chase without (−) or with dGTP (500 μM), dGTP + ddGTP (500 and 1000 μM, respectively), or an equimolar mix of dNTPs (500 μM each). After stopping the reactions with EDTA, products were incubated for 15 min ± protease K (0.32 U) and analyzed in a 20% polyacrylamide/urea gel. M, 5′-labeled 10-nt ssDNA ladder (left) and 5′-labeled dGMP_2_ and dGMP_3_ (right). Experiments in (A), (B), and (D) were repeated with similar results (fig. S12). Relative efficiencies of protein labeling by different dNTPs in (C) were the same as in (B).

To investigate whether protein priming could be used to initiate cDNA synthesis at the 3′-proximal CCC initiation site, we incubated Mm WT RT-Cas1/Cas2 without or with the 3′-blocked R50CCC_ddC template and [α-^32^P]-dGTP in the presence or absence of Mn^2+^. After the incubation, we analyzed the protein by SDS–polyacrylamide gel electrophoresis (SDS-PAGE) and autoradiography to detect covalently bound ^32^P-dGTP as expected for protein priming. As shown in Fig. 6A, incubation of Mm RT-Cas1/Cas2 with [α-^32^P]-dGTP for 15 min in the presence but not absence of the RNA template resulted in strong Mn^2+^-dependent labeling of RT-Cas1 as well as more weakly labeled low molecular weight bands that results below suggested were ^32^P-dG oligomers (Fig. 6A, lanes 1 to 4). Time courses showed that labeling of Mm RT-Cas1 by [α-^32^P]-dGTP increased progressively for times up to 60 min (fig. S4A). Adding an equimolar mixture of all four unlabeled dNTPs as a chase after the initial 15-min labeling period with [α-^32^P]-dGTP resulted in the appearance of a higher molecular weight labeled band that migrated above the major Coomassie blue–stained protein band, as well as increased intensity of the lower molecular weight bands (Fig. 6A, lane 5). These additional bands were insensitive to digestion with RNase A but sensitive to digestion with micrococcal nuclease (MNase), indicating that they were labeled cDNA products (lanes 6 and 7). Digestion with protease K shifted the major ^32^P-labeled protein band to a lower molecular weight and also resulted in disappearance of the labeled higher molecular weight MNase-sensitive band, suggesting that it was an extended cDNA associated with a fraction of the protein that was below the limit of detection by Coomassie blue staining (Fig. 6A, lane 8). The labeling of RT-Cas1 was not dependent on the presence of Cas2, and incubating Cas2 by itself under the same conditions did not result in labeled protein (Fig. 6A, lanes 9 and 10). The same experiment with a 3′-blocked 50-nt D50CCC_ddC template of the same sequence gave similar results, but with somewhat lower protein labeling intensity compared to that with the R50CCC_ddC template assayed in parallel (Fig. 6A, lanes 11 to 21). Collectively, these findings suggested that Mm RT-Cas1/Cas2 could initiate DNA synthesis on RNA or DNA templates via Mn^2+^-stimulated protein priming.

To test whether protein priming by RT-Cas1/Cas2 was restricted to using dGTP as the initiating nucleotide, we carried out a similar experiment in which RT-Cas1/Cas2 was incubated with the R50CCC_ddC template in the presence of each of the four [α-^32^P]-dNTPs (Fig. 6B). Analysis of the products on an SDS-PAGE gel showed that RT-Cas1 was labeled by all four [α-^32^P]-dNTPs with relative efficiency dGTP > dATP > dTTP > dCTP, and in each case, chasing with an equimolar mixture of unlabeled dNTPs (dN) resulted in higher and lower molecular weight bands corresponding to labeled DNA products that were degraded by MNase but not RNase A (Fig. 6B). These findings indicated that RT-Cas1/Cas2 could initiate DNA synthesis by protein priming with any dNTP, but with dGTP favored over dATP and purines favored over pyrimidines for initiation of DNA synthesis on the 3′-blocked R50CCC template.

To determine whether a 3′-proximal CCC sequence is essential for protein priming, we compared protein labeling using 3′-blocked 50-nt RNA or DNA templates of otherwise identical sequence in which the 3′-proximal CCC was changed to UUU/TTT or GGG (Fig. 6C). As in the previous experiment, RT-Cas1 was labeled by each of these dNTPs with efficiency dGTP > dATP > dCTP (Fig. 6C). However, chasing with the same unlabeled dNTP resulted in a smear of dissociated ^32^P-labeled DNA products extending up the gel lanes for RNA or DNA templates that had a complementary 3′-trinucleotide sequence but not for those that had a noncomplementary trinucleotide, as expected for reiterative copying of the trinucleotide in a sequence-dependent manner (Fig. 6C and see below).

To confirm that protein priming could give rise to free cDNA products, we incubated Mm RT-Cas1/Cas2 with [α-^32^P]-dGTP in reaction medium containing 10 mM Mg^2+^ and 1 mM Mn^2+^ for 7.5 or 15 min and then chased the reactions with higher concentrations of unlabeled dGTP or an equimolar mix of all four dNTPs for 30 min. The products were then analyzed on a denaturing 20% polyacrylamide gel before or after digestion with protease K. Phosphorimager scans of the gel showed that [α-^32^P]-dGTP covalently bound to RT-Cas1 (protease K–sensitive label in well) was chased into larger dissociated DNA products, near full-length cDNAs (marked by red stars in gel lanes) by higher concentrations of all four dNTPs, and progressively longer dG oligomers extending up the gel lane by higher concentrations of dGTP in the absence but not presence of ddGTP (Fig. 6D). The autoradiograms also showed a series of labeled bands (marked by small blue dots) that appeared after protease K digestion of products from the initial labeling period, but were not visible in the dNTP-chase lanes, suggesting that they corresponded to ^32^P-dGTP or short ^32^P-dG oligomers that remained covalently bound to RT-Cas1 at early time points but were chased into larger cDNA products that dissociated from the protein at later time points (Fig. 6D; see also fig. S4B).

Unexpectedly, the autoradiogram also showed a series of intensely ^32^P-labeled protease-insensitive lower molecular weight bands (up to ~20 nt) that were not appreciably chased into larger products by higher concentrations of dGTP or dNTPs. Further experiments showed that the intensely labeled bands were non–protein-associated ^32^P-dG oligomers as short as dinucleotides that accumulated over time in the absence but not appreciably in the presence of higher concentrations of dGTP or dNTPs at the beginning of the time course (fig. S4B). Instead, higher concentrations of dGTP at the beginning of the time course led to the synthesis of longer dG oligomers by reiterative copying of the CCC sequence, while higher concentrations of dNTPs at the beginning of the labeling period led to the synthesis of near full-length cDNAs of the RNA template (fig. S4B). These findings suggest that short DNA oligomers synthesized de novo or by rapid release after protein priming at early time points could be used to prime synthesis of longer DNA products.

Collectively, the above findings indicated that Mm RT-Cas1/Cas2 could use protein priming to initiate cDNA synthesis at 3′-proximal sites with different trinucleotide sequences, either reiteratively copying those sequences to generate DNA oligomers in the presence of a single complementary dNTP or synthesizing near full-length cDNAs initiated at the trinucleotide in the presence of all four dNTPs. Protein priming enabled initiation of cDNA synthesis with different efficiencies by any dNTP, including with dGTP at the 3′-proximal CCC containing a CC dinucleotide that is a preferred site for de novo initiation (*41*). These experiments also showed that Mm RT-Cas1/Cas2 synthesizes non–protein-associated DNA oligomers, either de novo or by rapid release after protein priming, with short DNA oligomers synthesized at early time points potentially used as primers for synthesis of longer cDNA products. Although our results do not preclude use of a primase mechanism for initiation of cDNA synthesis at some sites, time courses comparing rates of cDNA synthesis from the 3′-blocked R50CCC template with 500 μM dNTPs in the absence or presence of 20 μM dG_2_ primer showed a lag for initiation of DNA synthesis in the absence of the dG_2_ primer, most likely reflecting the time needed to synthesize a dG oligomer primer before beginning processive cDNA synthesis (fig. S4C).

### Mm RT-Cas1/Cas2 can initiate cDNA synthesis at complementary 3′-proximal sites by using 2-nt DNA oligonucleotide primers

**Fig. 7.**
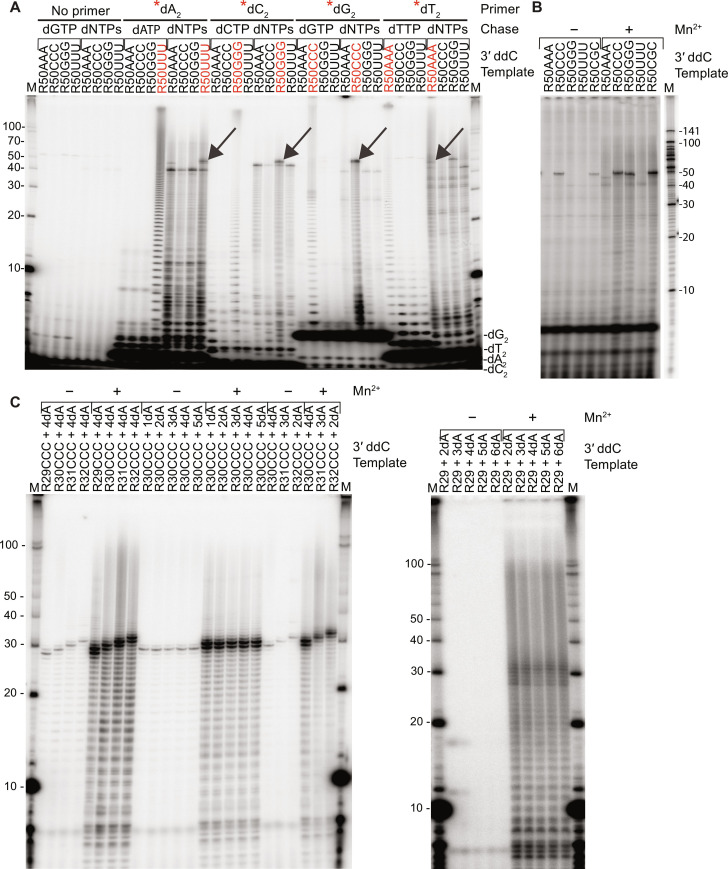
Mm RT-Cas1/Cas2 synthesizes near full-length cDNAs from different RNA templates by using multiple mechanisms to initiate cDNA synthesis at different 3′ proximal sites. (**A**) Gel analysis of cDNAs synthesized by Mm WT RT-Cas1/Cas2 using exogenous dinucleotide primers. Reactions were done by incubating RT-Cas1/Cas2 (500 nM) with 250 nM 3′-blocked RNA templates with 3′-proximal AAA, CCC, GGG, UUU, or CGC without or with 20 μM 5′-^32^P-labeled (*) dA_2_, dC_2_, dG_2_, and dT_2_ dinucleotide primers in reaction medium containing 10 mM MgCl_2_ without MnCl_2_. After incubating for 30 min at 25°C, the reactions were chased for 30 min with 500 μM of the dNTP matching the dinucleotide primer or an equimolar mix of all four dNTPs (500 μM each). (**B**) Gel analysis of cDNAs synthesized from the same templates as in (A) without an added primer. RT-Cas1/Cas2 was incubated with RNA templates (250 nM) and ^32^P-labeled dNTPs ([α-^32^P]-dCTP, dATP, dGTP, and dTTP, 500 μM each) for 1 hour at 25°C. (**C**) Gel analysis of cDNAs synthesized from 3′-blocked templates with varying length RNA segments and 3′-dA tails. Reactions were done with 500 nM RT-Cas1/Cas2, 250 nM template, and ^32^P-labeled dNTPs (20 μM [α-^32^P]-dCTP plus 500 μM dATP, dGTP, and dTTP) in reaction medium containing 10 mM MgCl_2_ ± 1 mM MnCl_2_ for 1 hour at 25°C. ^32^P-labeled cDNAs were analyzed in a denaturing 20% polyacrylamide gel against a 5′-labeled 10-nt ssDNA ladder (lane M). A full repeat was done for all samples in (B) and most samples in (A) and (C) with similar results (fig. S13). Gel analysis for all samples was consistent with cDNA start sites identified by TGIRT-seq (Figs. 5 and 8).

To test systematically whether Mm RT-Cas1/Cas2 could use short DNA oligomer primers to initiate DNA synthesis at complementary sites in an RNA template, we incubated Mm WT RT-Cas1/Cas2 with 3′-blocked RNA templates (R50NNN_ddC) with different 3′-proximal trinucleotide sequences (AAA, CCC, GGG, or UUU) and 5′-labeled dA_2_, dC_2_, dG_2_, or dT_2_ dinucleotide primers. The reactions were done in the presence of high concentrations of the unlabeled dNTP matching the dinucleotide (e.g., dATP for the dA_2_ primer) or an equimolar mixture of all four unlabeled dNTPs in the absence of Mn^2+^ to minimize terminal transferase addition to the dN_2_ primer (visible as short DNA ladders in some gel lanes; Fig. 7A). Analysis of the products in a denaturing 20% polyacrylamide gel showed that when incubated with high concentrations of the same unlabeled dNTP, RT-Cas1/Cas2 reiteratively copied the complementary trinucleotide in each template, generating a ladder of DNA homopolymers extending up the gel lane for those RNA templates that contained the complementary trinucleotide but not for the other templates (Fig. 7A). When the reactions were done with an equimolar mixture of all four unlabeled dNTPs, RT-Cas1/Cas2 switched from reiterative copying of the complementary 3′ trinucleotide to synthesis of prominent longer cDNAs extending up to ~50-nt cDNAs only for those templates that contained the complementary 3′ trinucleotide (including both R50AAA and R50GGG for the dT_2_ primer; Fig. 7A).

**Fig. 8.**
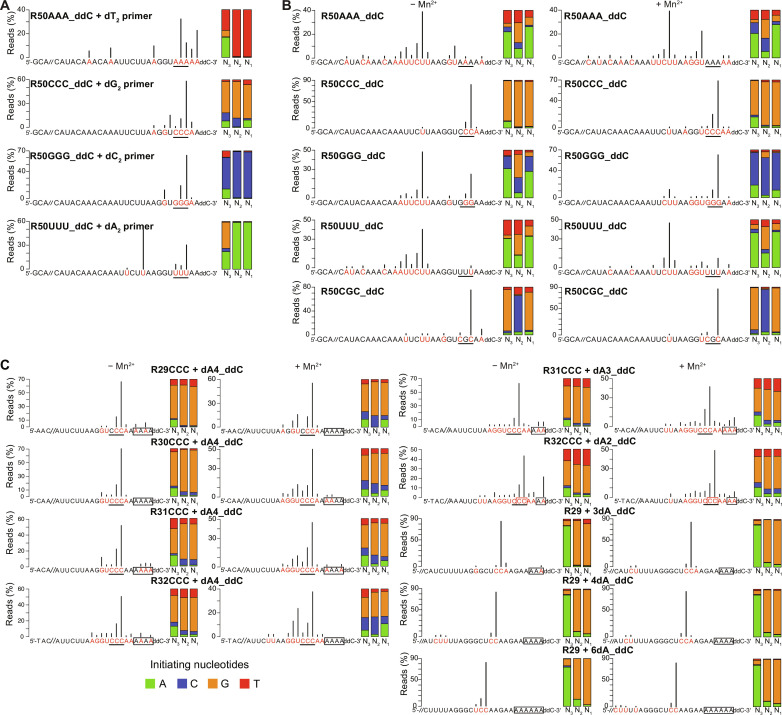
TGIRT-seq of cDNAs synthesized by Mm RT-Cas1/Cas2 from different RNA templates in the presence or absence of added dinucleotide primers. (**A**) TGIRT-seq of cDNAs from reverse transcription reactions using the same 3′-blocked RNA templates as in Fig. 7A with unlabeled dinucleotides and an equimolar mix of all four unlabeled dNTPs (500 μM each). Reverse transcription reactions were done for 1 hour at 25°C in reaction medium containing 10 mM MgCl_2_, and cDNAs were analyzed by TGIRT-seq, as described in Materials and Methods. Nucleotides in the RNA template that correspond to the first nucleotide of a cDNA product are highlighted (red) with black vertical lines indicating the percentage of reads beginning at that nucleotide. The 3′-proximal trinucleotide sequences in templates are underlined. The stacked bar graphs (right) show the proportions of different nucleotides at positions N_1_ to N_3_ of the cDNAs [color code shown below (C) at bottom left]. (**B**) TGIRT-seq analysis of cDNAs synthesized from the same 3′-blocked templates as in Fig. 7B in the absence of an added primer. Reverse transcription reactions were done with an equimolar mix of all four dNTPs (500 μM each) in reaction medium containing 10 mM MgCl_2_ ± 1 mM MnCl_2_ for 1 hour at 25°C. The datasets for the R50CCC_ddC and R50CGC_ddC templates were the same as those in Fig. 5. (**C**) TGIRT-seq of cDNAs synthesized from 3′-blocked RNA templates with different length RNA and dA-tail segments. The RNA templates were the same as those in Fig. 7C with reverse transcription reactions done with an equimolar mix of all four dNTPs (500 μM each) in reaction medium containing 10 mM MgCl_2_ ± 1 mM MnCl_2_ for 1 hour at 25°C. cDNA start sites for all templates were consistent with gel analysis of labeled cDNAs in Figs. 4 and 7.

Sequencing of the cDNAs synthesized after the dNTP chase in parallel reactions with unlabeled dN_2_ primers confirmed that the initiation sites were complementary di- or trinucleotide sequences, with the complementary 3′-proximal trinucleotide sequence a major initiation site in all cases (Fig. 8A). The dG_2_ primer also showed a low background of cDNA start sites at U, A, or G residues found by sequencing to reflect a background of de novo or protein-primed initiations rather than non–Watson-Crick pairings of the dG_2_ primer (Fig. 8A) (*53*). The ability to use very short DNA primers to faithfully initiate cDNA synthesis at complementary sites is enabled by the strong strand-annealing activity of group II intron-like RTs (*22*).

### Mm RT-Cas1/Cas2 initiation sites in the absence of an added primer are dependent on the stability of base-pairing interactions over several nucleotides

To investigate factors that affect the choice of cDNA initiation sites in the absence of an added primer, we incubated Mm WT RT-Cas1/Cas2 with the same 3′-blocked R50NNN templates with different 3′-proximal trinucleotide sequences and an equimolar mix of 500 μM [α-^32^P]-dCTP, dATP, dGTP, and dTTP in the absence or presence of Mn^2+^. Analysis of the products in a denaturing 20% polyacrylamide gel showed prominent labeled cDNAs of ~50 nt suggestive of initiation of cDNA synthesis at the 3′-proximal trinucleotide sequence for the 3′-blocked R50CCC template in the absence or presence of Mn^2+^, as well as for the mutant R50CGC and R50GGG templates in the presence of Mn^2+^ (Fig. 7B). By contrast, the most prominent labeled bands for the R50UUU and R50AAA templates in the absence or presence of Mn^2+^ were a series of shorter cDNAs ranging in size from 30 to 40 nt, with little if any indication of initiation at the 3′-proximal UUU or AAA sequences (Fig. 7B).

To identify putative cDNA initiation sites, we sequenced the cDNAs synthesized from the different RNA templates in parallel reactions with unlabeled dNTPs in the presence or absence of Mn^2+^ (Fig. 8B). The sequencing confirmed that most (75 to 92%) of the cDNAs synthesized from the R50CCC_ddC and R50CGC_ddC templates in the presence or absence of Mn^2+^ began at the 3′-proximal CCC and CGC trinucleotide sites, with <15% beginning opposite A or U residues (Fig. 8B, rows 2 and 5). The first nucleotide of most of the cDNAs initiated on the CCC and CGC templates corresponded to a G residue, while the second nucleotide corresponded to a G residue for the CCC template and a C residue for the CGC template, as expected for de novo initiation (*41*) or protein priming at the 3′-proximal C residue of the trinucleotide in both templates (Fig. 8B). None of the cDNAs synthesized from the CGC template began at the middle G residues, reflecting that dCTP is used less efficiently for de novo or protein-primed initiation of cDNA synthesis than dGTP (Fig. 8B).

Last, switching the 3′-proximal trinucleotide to GGG also resulted in increased use of initiation sites within the nearby upstream AAUUCUU sequence but with a high proportion of cDNAs initiated at the 3′-proximal GGG sequence in the absence of Mn^2+^ and with the 3′-proximal GGG becoming the predominant initiation site in the presence Mn^2+^ (Fig. 8B, row 3). In this case, the major, almost exclusively used initiating nucleotide in the presence of Mn^2+^ was dCTP, which can form a stable base pair with G but is the least favored dNTP for protein priming and likely de novo initiation by RT-Cas1/Cas2 (Fig. 6B). Collectively, these findings indicate that in addition to proximity to the 3′ end of the RNA, the stability of base-pairing interactions over several nucleotides can override specific sequences for the choice of cDNA initiation sites on RNA templates, as expected for initiation of cDNA synthesis by annealing of short newly synthesized DNA oligomer primers.

### Mm RT-Cas1/Cas2 initiates at 3′-proximal sites in the RNA segment of 3′-dA–tailed RNAs

To investigate how cDNA synthesis is initiated on RNA protospacers with 3′-DNA tails, we tested the effect of varying the lengths of the RNA and 3′-DNA-tail in two different sets of 3′-blocked RNA protospacers, one set (R29-32CCC + dA_n_) corresponding to the 3′ end of the R50CCC RNA template used above to identify cDNA initiation sites (Figs. 5 and 8), and the other set (R29 + dA_n_) corresponding to the R29 RNA used to analyze RNA protospacer integration with or without a 3′-dA tail (Fig. 3). For both sets of templates, we found that preferred initiation sites in the presence or absence of Mn^2+^ were located toward the 3′ end of the RNA segment, with few initiations occurring within the 3′-dA tail (gels shown in Fig. 7C, sequencing results shown in Fig. 8C). The sequences showed that the major initiation sites for the R29-32CCC + dA_n_ templates were clustered at or near the CCC trinucleotide, the favored initiation site for the R50CCC template, but with higher proportions of initiations outside the CCC sequence, while the major initiation sites for the R29 + dA_n_ templates were at a 3′-proximal CC dinucleotide sequence with minor initiation sites elsewhere in the RNA segment and few or none in the 3′-dA tail (Fig. 8C). The initiation of cDNA synthesis at 3′-proximal sites in the RNA segment upstream of the 3′-dA tail yields RNA/DNA duplexes with a single-stranded 3′-DNA overhang, which is favored for integration dsDNA protospacers into CRISPR arrays by Cas1/Cas2 (*54*).

### Kinetic analysis of RNA and DNA protospacer integration suggests preferred mechanisms

**Fig. 9.**
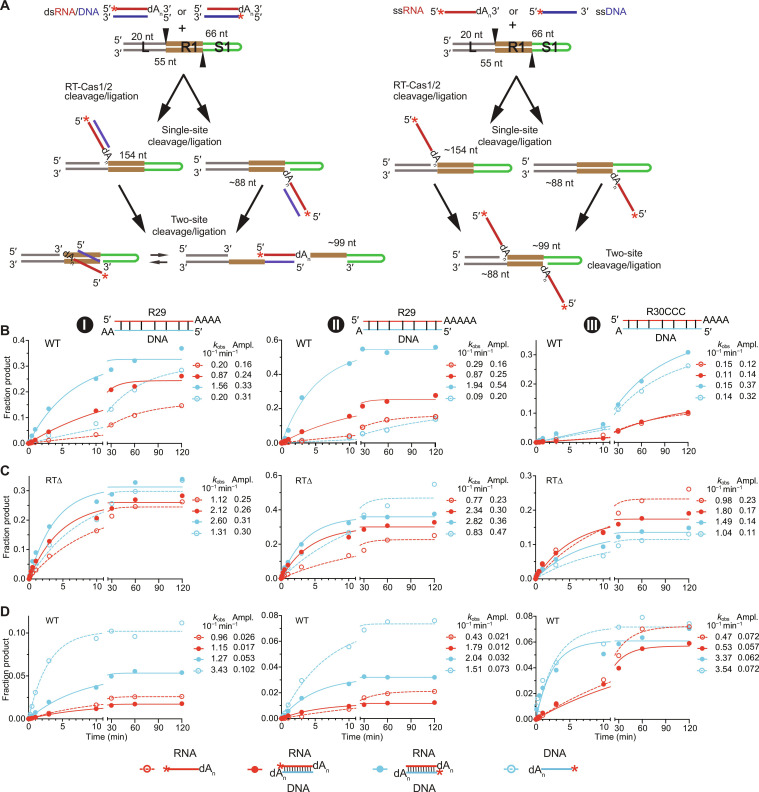
Time courses comparing rates of integration of 3′-dA–tailed RNA, ssDNA, and RNA-dA/DNA duplex protospacers into a CRISPR DNA. (**A**) Schematic of spacer integration assays using a CRISPR DNA with a 5′ leader (L, gray), repeat (R1, brown), and hairpin segment corresponding to the first spacer (S1, green). Spacer integration reactions were done by incubating 5′-labeled (*) 3′-dA–tailed RNAs (*RNA-dA, red) or ssDNAs (*DNA, blue) with or without a cDNA strand (blue) that leaves different length 3′-dA overhangs on both strands. For two-site cleavage ligation of duplex protospacers, only the insertion of the RNA into the top strand is shown. (**B**) Time courses for production of the labeled 99-nt band resulting from cleavage/ligation reactions at the 5′ end of R1 on both strands for three sets of protospacers (I, II, and III) composed of 3′-dA–tailed R29 or R30CCC RNAs without or with a cDNA strand. Reactions were done by incubating WT RT-Cas1/Cas2 (500 nM) and CRISPR hairpin DNA (100 nM) with a 5′ ^32^P-labeled (*) protospacer (5 nM) for up to 2 hours with products analyzed in an 8% polyacrylamide/urea gel (phosphorimager scans are shown in fig. S14). Plots for *RNA-dA, *RNA-dA/DNA, RNA-dA/*DNA, and *DNA protospacers are color-coded as shown at the bottom of the figure. The data were fit to a single exponential equation to calculate rates (*k*_obs_) and amplitudes (Ampl.). *R*^2^ values are listed in table S2. (**C**) Spacer integration by RTΔ-Cas1/Cas2 for the same sets of protospacers. (**D**) Spacer integration by WT RT-Cas1/Cas2 for the same sets of protospacers at 250 nM protospacer concentration. Time courses in (B) to (D) were done once in parallel for each set of protospacers with conclusions based on consistent trends for RNA-dA, ssDNA, or RNA-dA/DNA duplex protospacers that differed in sequence and length of 3′-DNA overhangs.

Last, to investigate the mechanism by which 3′-dN tailed RNA protospacers are integrated into CRISPR arrays, we carried out kinetic assays of spacer integration. For these assays, we used a CRISPR hairpin DNA substrate in which the top and bottom strands were connected by a 5-nt linker, making it possible to identify products resulting from coupled cleavage ligation of 5′-labeled protospacers into either or both strands by the length of labeled DNA fragments (Fig. 9A). The reactions were done in the absence of Mn^2+^ or dNTPs with three sets of R29-dA_n_ or R30CCC-dA_n_ oligonucleotide protospacers (denoted I, II, and III) without or with a cDNA strand that leaves different length 3′-dA overhangs on both strands of duplex protospacers (Fig. 9, B to D). For each set, we compared the rates of integration by WT RT-Cas1/Cas2 or RT∆-Cas1/Cas2 in parallel assays with four different 5′-labeled (denoted by *) protospacers: a 3′-dA–tailed RNA (*RNA-dA_n_), a single-strand *DNA, an *RNA-dA_n_/DNA duplex with the RNA strand labeled, and an identical RNA-dA_n_/*DNA duplex with the DNA strand labeled, making it possible to separately quantitate the integration of each strand (plots shown in Fig. 9, B to D; gels shown in fig. S14, A to C). The integration of a stable *RNA-dA_2_/cDNA duplex in a similar spacer integration assay was confirmed by gel electrophoresis of labeled integration products before and after RNase H digestion (fig. S5).

For Mm WT RT-Cas1/Cas2 at a relatively low protospacer concentration (5 nM), the rates and amplitudes for appearance of the 5′-labeled 99-nt cleavage-ligation product resulting from spacer integration into both strands were with one exception higher for the *RNA-dA_n_/DNA or RNA-dA_n_/*DNA duplexes (closed red and blue circles) than for their single-stranded *RNA-dA_n_ or *ssDNA counterparts (open red and blue circles), as expected for duplexes enabling more rapid sequential ligation into the top- and bottom-strands (Fig. 9B; time courses for all labeled cleavage products shown in fig. S6A). The amplitudes determined from the curve fits for integration of *RNA-dA_n_ or the *RNA-dA_n_/DNA duplex (red open and closed circles, respectively) were lower than those for their *ssDNA or RNA-dA_n_/*DNA counterparts (blue open and closed circles, respectively; Fig. 9B), indicating less efficient integration of the RNA strand by WT RT-Cas1/Cas2 under these conditions.

When the same reactions were done with RTΔ-Cas1/Cas2, the rates for integration increased for all substrates tested (Fig. 9C). However, the increases in rates and amplitudes were larger for the *RNA-dA_n_ and *RNA-dA_n_/DNA duplexes (red open and closed circles, respectively) than for their *DNA counterparts (blue open and closed circles), resulting in more similar and in one case higher rates and amplitudes for the *RNA-dA_n_ and *RNA-dA_n_/DNA protospacers than for their similarly configured *DNA counterparts (Fig. 9C). These findings likely reflect that the RT domain impedes RNA-dN spacer integration when not directly coupled to cDNA synthesis and suggest that there may be relatively little inherent difference in the efficiency of integration of dN-tailed RNA and DNA protospacers.

Notably, when the spacer integration reactions with WT RT-Cas1/Cas2 were done at higher protospacer concentrations (250 nM), both the single-stranded *RNA-dA_n_ and *ssDNA protospacers (red and blue open circles, respectively) had higher rates and amplitudes than did their labeled duplex counterparts (red and blue closed circles, respectively), with the higher efficiency for ssDNA protospacers particularly notable for two of the three protospacer configurations tested (Fig. 9D). These findings suggested that integration of single-strand dN-tailed RNA and ssDNA protospacers might be favored in vivo for abundant pathogen RNAs and ssDNA fragments generated by RecBCD or other phage defense nucleases (*55*–*59*). Parallel assays comparing the integration efficiencies of protospacers with an RNA-dA_n_ strand to all-DNA versions of the same protospacer suggested that, in a number of cases, differences in rates and amplitudes between each set of protospacers largely reflected the sequence of the protospacer rather than whether it was an RNA or DNA (fig. S7 compare to Fig. 9 and fig. S6).

## DISCUSSION

**Fig. 10.**
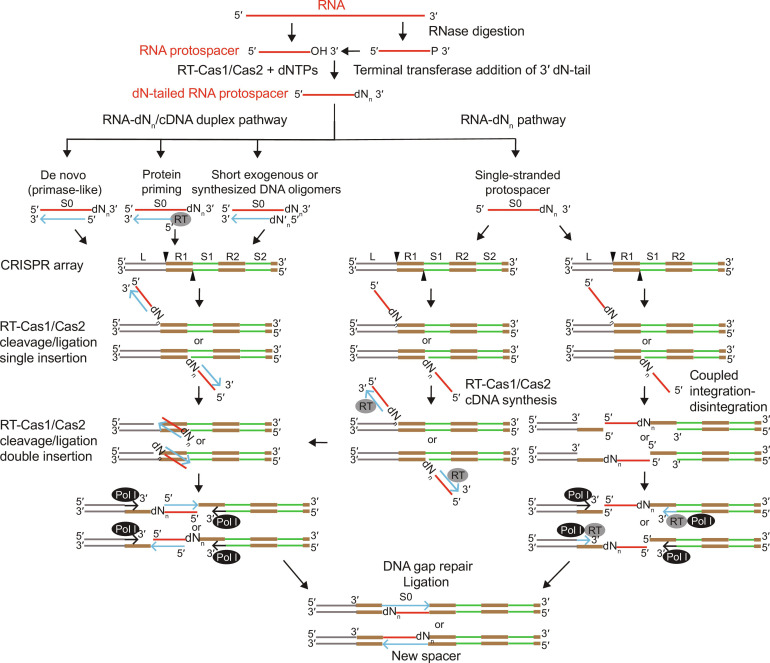
Possible pathways and mechanisms used for RNA spacer acquisition by RT-Cas1/Cas2 proteins. All pathways start with RT-Cas1/Cas2 terminal transferase activity adding short 3′-DNA tails (dN_n_) to host cellular or pathogen RNA fragments resulting from cleavage by RNases that leave either a 3′ OH or a 3′ phosphate that can be removed enzymatically to leave a 3′ OH. On the left are pathways in which RT-Cas1/Cas2 uses different mechanism (de novo initiation, protein priming, or exogenous or synthesized short DNA oligomer primers) to synthesize cDNAs that remain annealed to the RNA-dN template in an RNA-dN/cDNA duplex. The duplex protospacers are then integrated into opposite strands of the CRISPR array by RT-Cas1/Cas2 via a mechanism analogous to that used by conventional Cas1/Cas2 proteins to integrate duplex DNA protospacers. On the right are alternative pathways in which 3′-dN–tailed RNA protospacers are integrated into the CRISPR array before cDNA synthesis. The integrated RNA-dN protospacers could then be used as a template for cDNA synthesis by RT-Cas1/Cas2, resulting in an RNA-dN/cDNA duplex that becomes fully integrated into the CRISPR array by a mechanism analogous to the second step for integration of DNA duplex protospacers (horizontal arrow pointing left). Alternatively, a single-stranded RNA-dN protospacer integrated at one site could in principle be integrated into the opposite strand by a coupled integration/disintegration reaction, resulting in DNA gaps that are filled in by RT-Cas1/Cas2 or a cellular DNA polymerase, e.g.*,* Pol I, which has innate ability to copy RNA into DNA (*60*, *68*). In all pathways, protospacer integration leaves DNA segments with single-stranded gaps held together by bound RT-Cas1/Cas2 and possibly other proteins until they are displaced by transcription-coupled DNA repair, enabling complete integration of the spacers without introducing deleterious double-stranded breaks (*39*).

Here, we elucidated biochemical mechanisms underlying pathways that could be used by Mm RT-Cas1/Cas2 for cDNA synthesis and site-specific integration of RNA protospacers into CRISPR arrays (Fig. 10). All pathways begin with RT-Cas1/Cas2 using its terminal transferase activity to add short DNA tails to the 3′ ends of RNA fragment protospacers generated by RNases in vivo. In one set of pathways (Fig. 10, left), RT-Cas1/Cas2 synthesizes near full-length cDNAs of 3′-dN–tailed RNAs by using multiple mechanisms to initiate cDNA synthesis at 3′-proximal sites of diverse RNAs, including de novo initiation, protein priming, and annealing of short exogenous or synthesized DNA oligomer primers. The resulting 3′-dN–tailed RNA/DNA duplexes with deoxynucleotides at the 3′ ends of both strands are then ligated into the CRISPR array by a mechanism analogous to that used by conventional Cas1/Cas2 proteins to integrate dsDNA protospacers. In a second set of pathways favored at higher protospacer concentrations (Fig. 10, right), 3′-dN–tailed RNAs are integrated directly into the CRISPR array before cDNA synthesis. Reverse transcription of the 3′-dN–tailed RNA by RT-Cas1/Cas2 could then occur after integration (left) or after linked integration/disintegration reactions at CRISPR insertion sites on opposite strands (right) with single-stranded gaps filled in by RT-Cas1/Cas2 or host DNA polymerase I (Pol I) (*60*). The latter mechanisms could also be used for the integration of suitably sized ssDNA protospacers generated by RecBCD or other phage defense nucleases (*39*, *57*, *60*). In all pathways, the resulting gapped DNA after protospacer integration is held together by RT-Cas1/Cas2 possibly assisted by cellular DNA damage control proteins until their dissociation by transcription-coupled DNA repair that fully integrates the newly acquired spacer into the CRISPR array (*39*). Our analysis of these mechanisms explains known features of RNA spacer acquisition by Mm RT-Cas1/Cas2 in vivo and revealed two novel biochemical activities with potential biotechnological applications: the ability of an RT to use multiple mechanisms to synthesize near full-length cDNAs of diverse RNA templates without an added primer or fixed sequence requirements, and the ability of a DNA integrase to site-specifically integrate RNAs into DNA genomes by adding deoxynucleotides at crucial locations.

The requirement for adding short 3′-dN tails to enable direct integration of RNA protospacers into a CRISPR array was suggested by the finding that spacers acquired by RT-Cas1/Cas2 proteins in vivo have larger numbers of noncoded nucleotides at spacer-repeat junctions than do those acquired by Mm RTΔ-Cas1/Cas2 or conventional Cas1/Cas2 proteins that acquire spacers from DNA (Fig. 1B). Biochemical assays supported this mechanism by showing that the noncoded nucleotides at spacer-repeat junctions of spacers acquired by Mm RT-Cas1/Cas2 in vivo matched the nucleotide preferences of Mm RT-Cas1/Cas2 terminal transferase in vitro (Figs. 1C and 2) and that Mm RT-Cas1/Cas2 could directly integrate single-stranded 3′-dN–tailed RNA protospacers into a CRISPR DNA both in coupled terminal transferase/spacer-ligation reactions in the presence of a single nucleotide (dATP) and in uncoupled spacer ligation reactions using synthetic RNA oligonucleotides with different length 3′-dA tails (Fig. 3). The latter assays showed that addition of a single 3′-dA residue enabled detectable RNA protospacer integration and addition of two to six 3′-dA residues increased rates of RNA protospacer integration to levels comparable to those of ssDNA protospacers with the same nucleotide sequence (Fig. 3).

RNA protospacers with a 3′ OH needed for terminal transferase addition of DNA tails could be generated by cellular RNases, such as RNase III, which cleaves structured RNAs and directly generates RNA fragments with a 3′ OH (*61*); by phosphatase removal of 3′ phosphates left by other RNases; or by an inherent RT-Cas1/Cas2 endonuclease activity that cleaves upstream of a 3′-blocked RNA, an activity reported for telomerase RT (*62*). Unlike type I and II CRISPR systems, whose surveillance complexes recognize target DNAs by binding a protospacer adjacent motif (PAM) and then checking for complementarity to a seed region corresponding to the first ~5 nt of the guide RNA, the seed regions of type III CRISPR systems surveillance complexes are internal and thus unlikely to be affected by noncoding nucleotides at the 5′ or 3′ end of the protospacer (*63*). This difference likely accounts for why RTs are associated with type III CRISPR systems but not CRISPR systems that use a PAM (*64*, *65*).

As desired for an enzyme whose biological function is to acquire RNA-derived spacers that defend against diverse pathogens, we found that Mm RT-Cas1/Cas2 could use multiple mechanisms to initiate cDNA synthesis on RNA protospacers at different 3′-proximal sites with no fixed sequence requirements. In addition to recently reported de novo initiation with a strong preference for initiating at CC sequences (*41*), we found that Mm RT-Cas1/Cas2 can use protein priming to initiate at multiple 3′-proximal sites with any dNTP, greatly expanding the number of suitable cDNA initiation sites (Figs. 6 to 8). We also found that Mm RT-Cas1/Cas2, like other group II intron-related RTs (*22*), has a strong strand-annealing activity that enables it to initiate site-specific cDNA synthesis by using primers as short as dinucleotides (Fig. 7A). Short DNA oligomer primers could be generated in vivo by cellular enzymes, including RecBCD, which selectively degrades pathogen DNAs not only into ssDNA fragments long enough to be acquired as spacers but also into ssDNA fragments as short as 3 to 6 nt that could be used to preferentially prime DNA synthesis on pathogen nucleic acids (*59*, *66*).

Our findings suggest that Mm RT-Cas1/Cas2 can also use de novo initiation or protein priming to synthesize its own short DNA oligomer primers that initiate DNA synthesis at complementary sequences on RNA or DNA templates (Figs. 6 to 8). Supporting this mechanism, we found that the stability of base-pairing interactions over several nucleotides plays a substantial role in the selection of cDNA initiation sites (Figs. 7 and 8). Most compelling was the finding that both CCC and GGG at the same 3′-proximal location in otherwise identical RNA templates were both preferred initiation sites for cDNA synthesis (Fig. 8B), although dCTP is the least favored dNTP for protein priming (Fig. 6B) and likely also for de novo initiation. Initiation at G residues with dCTP at the 3′-proximal GGG sequence trumped initiation at sites with C, U, or A residues that were favored for cDNA initiation in otherwise identical templates that lacked a 3′ GGG (Fig. 8B). Although our results do not exclude a primase-like mechanism, time courses for initiation of cDNA synthesis at a 3′-proximal CCC sequence with 500 μM dNTPs showed a pronounce lag for initiation of cDNA synthesis compared to a parallel assay in the presence of 20 μM dinucleotide dG_2_ primer, most likely reflecting the time needed for RT-Cas1/Cas2 to synthesize a short DNA primer (fig. S4C).

Unlike AbiK and Abi-P2 RTs, which synthesize random sequence DNA oligomers in the absence of an RNA or DNA template (*20*), the synthesis of short DNA oligomers by Mm RT-Cas1/Cas2 required the presence of an RNA template (Figs. 4A and 6A). AbiK and Abi-P2 RTs have distinctive structural features that prevent binding of a template but leave the RT active site accessible to dNTPs (*20*), whereas group II intron RT apoenzymes have a tendency to fold into an inactive structure in which the RT active site is blocked until activated by template binding (*67*). At this stage, we cannot distinguish whether the synthesis of short DNA oligomer primers by Mm RT-Cas1/Cas2 occurs by a de novo or protein-primed, nontemplated mechanism like that used by the Abi RTs (*20*) or by transient association with an RNA template until the primer has sufficient length and base-pairing stability to initiate processive DNA synthesis. The finding that group II intron, human LINE-1, telomerase, and retroviral RTs are capable of Mn^2+^-dependent de novo initiation of cDNA synthesis (*41*) raises the possibility that de novo synthesis of short DNA oligomer primers may be an inherent activity of many if not all RTs.

The ability of RT-Cas1/Cas2 to integrate RNA-dN/cDNA duplexes with overhanging ssDNA tails into a CRISPR array is consistent with and in retrospect might have been predicted from the structures of dsDNA integration complexes of Cas1/Cas2 from CRISPR systems that lack an associated RT (*25*, *34*, *54*). These structures showed that the Cas1/Cas2 proteins form a hexameric complex with two Cas1 homodimers on either side separated by a Cas2 homodimer. The latter forms a platform for binding a DNA duplex across the length of the complex via non–sequence-specific base and phosphate-backbone interactions with splayed or overhanging single-stranded 3′-DNA ends of the two DNA strands inserted into Cas1 active sites on opposite sides of the complex. The findings that Mm RT-Cas1/Cas2 initiates cDNA synthesis on dA-tailed RNAs at 3′-proximal sites within the RNA segment (Fig. 8C) and adds small numbers of noncoded nucleotides to the 3′ ends of completed cDNAs (Fig. 5) have the desirable effect of generating RNA/cDNA duplexes with single-strand DNA overhangs on both strands, potentially making them favorable substrates for integration by Cas1/Cas2. The inability of Mm RT-Cas1/Cas2 proteins to integrate RNA spacers without short DNA tails may reflect steric hindrance by 2′ OH groups that impede binding of 3′-terminal nucleotides at the Cas1 active sites, with this hindrance possibly less for the *Thiomicrospira* RT-Cas1/Cas2, which was reported to inefficiently but detectably integrate RNAs lacking a 3′-dN tail (*14*, *25*).

Previous findings showed that the Mm and *Thiomicrospira* RT-Cas1/Cas2 differ from conventional Cas1/Cas2 proteins in being able to efficiently integrate ssDNA as well as dsDNA protospacers into CRISPR arrays, likely reflecting structural differences in the RT-Cas1/Cas2 integrase that relax strict requirements for protospacer binding (*12*, *25*). Here, we extended these findings by showing that Mm RT-Cas1 could efficiently integrate 3′-dN RNAs into CRISPR arrays, with integration of duplex protospacers favored at lower protospacer concentrations and single-stranded protospacers favored at higher protospacer concentrations. The favored integration of duplex protospacers at lower protospacer concentrations likely reflects that they are more stably bound than single-stranded protospacers by phosphate-backbone contacts to both strands and/or rapid sequential binding of the two 3′ ends to Cas1 active sites on opposite sides of the complex. The steeper concentration dependence for integration of a single-stranded protospacer may reflect that its less constrained binding to one active site is rate-limiting at lower protospacer concentrations but more readily overcome at higher protospacer concentrations. This concentration dependence may be a built-in mechanism that favors protospacer acquisition from abundant RNAs produced by invading pathogens as well as ssDNA fragments generated by degradation of invading pathogen DNAs by RecBCD or other phage defense nucleases (*42*, *57*). Also noteworthy was that deletion of the RT domain in Mm RT∆-Cas1/Cas2 increased the rate and amplitudes of protospacer integration for RNA-dN and RNA-dN/DNA-dN duplexes to a greater extent than similarly configured DNA-dN protospacers (Fig. 9 and figs. S6 and S7). This finding likely reflects that the RT domain impedes RNA spacer integration when not directly coupled to cDNA synthesis and suggests that there may be little inherent difference in the integration efficiencies of dN-tailed RNA and DNA protospacers by the Cas1/Cas2 integrase domain.

Other possible mechanism for RNA protospacer acquisition include (i) snap-back cDNA synthesis followed by RNase digestion of the single-strand RNA loop leaving an RNA/cDNA heteroduplex and (ii) RNase H digestion of the RNA strand of the initial RNA-dA/cDNA heteroduplex followed by second-strand DNA synthesis by RT-Cas1 or a host DNA polymerase to generate a dsDNA protospacer. While neither mechanism can be completely excluded, snap-back DNA synthesis would not generate an RNA-DNA duplex with a DNA-tail at the 3′ end of the RNA strand, while RNase H digestion of the RNA strand of an initial RNA/cDNA or RNA-dN/cDNA duplex followed by second-strand DNA synthesis would result in dsDNA protospacers lacking the distinctive ≥2-nt noncoded nucleotide tails added to and required for integration of RNA protospacers by Mm RT-Cas1/Cas2 (Figs. 1C and 3).

The spacers acquired by Mm WT RT-Cas1/Cas2 in its native host were positively correlated with gene expression levels and integrated in either orientation into the CRISPR array with no specific sequence requirements or enrichment for specific regions of the RNA [(*12*) and fig. S1D]. The finding that deletion of the RT domain abolished the correlation between spacer acquisition and gene expression levels (*12*) together with the characteristics of the integrated spacers suggest that spacer acquisition by WT RT-Cas1/Cas2 is dependent primarily on RNA abundance, with little or no distinction between host and pathogen RNAs. The integration of protospacers in either orientation by Cas1/Cas2 reflects that type III CRISPR systems have no requirement for a PAM, enabling double-stranded protospacers to bind the two Cas1 active sites in either orientation and for single-stranded protospacers to bind to either Cas1 active site for integration into the top or bottom strand. Ma *et al.* (*60*) suggested that dsDNA protospacers with one 3′ end inserted into either strand of the CRISPR array could become fully integrated simply by coupled integration/disintegration reactions followed by transcription-coupled DNA repair. Our finding that single-stranded 3′-dN RNAs can be site-specifically integrated into a CRISPR array raises the intriguing possibility that only the terminal transferase activity RT-Cas1/Cas2 proteins might be required for RNA protospacer integration in bacteria containing an enzyme that could copy a short RNA sequence into DNA [e.g., another RT or a host DNA polymerase such as polymerase I that has an innate ability to copy RNA (*68*); Fig. 10, right]. The acquisition of RNA-derived spacers in *M. mediterranea* required overexpression of RT-Cas1/Cas2 and was not seen with endogenously expressed proteins (*12*). These findings may reflect that RT-Cas1/Cas2 overexpression is activated as a last resort when pathogen RNAs reach high copy number by evading other cellular defense mechanisms. Under those circumstances, the overexpressed RT-Cas1/Cas2 has no compunction about integrating host-derived RNA protospacers, potentially leading to autoimmunity and altruistic cell death. Although our study revealed mechanisms used by an RT-Cas1/Cas2 protein for reverse transcription of RNA-derived spacers into a CRISPR array, further studies are required to elucidate the structural basis for these mechanisms and to assess the contribution of different mechanisms to spacer acquisition in vivo.

Phylogenetic analyses indicated that CRISPR-associated RTs evolved in at least three different occasions, with the largest and most ancient clade having an RT domain closely related to that of group II introns RTs, and the smaller more recently evolved clades having an RT domain more closely related to those of Abi-P2 or retron RTs (*69*), both of which likely evolved earlier from a group II intron RT. RT-Cas1 fusion proteins comprise a single evolutionary branch of CRISPR-associated RTs, while freestanding CRISPR-associated RTs in other branches are juxtaposed and transcribed in the same direction as genes encoding Cas6 and Cas1/Cas2 proteins, suggesting that a functional link between these proteins preceded the fusions (*13*, *14*). The phylogenetic branching patterns also suggested that fusion of RT to Cas1 occurred first, followed by more recent fusion to Cas6, and that both fusions have been horizontally transferred to different type III and a few type VI CRISPR systems, suggesting an inherent ability to function together with other Cas1/Cas2 proteins (*14*, *65*). Frequent horizontal transfer and rapid sequence divergence make it difficult to distinguish whether RT-Cas1 fusion occurred once or independently on multiple occasions, which would further support a selective advantage for coordinating linked functions (*69*).

Mobile group II intron-encoded RTs, the likely ancestors of CRISPR-associated RTs, have throughout evolution exhibited remarkable flexibility to modulate their biochemical activities and acquire additional domains that optimize their biological functions. Bacterial examples include diversity generating retroelement RTs, which preferentially misincorporate specific dNTPs into protein-coding regions to enable host-phage tropism switching (*70*, *71*); retron RTs and abi RTs, which use different mechanisms to synthesize ssDNAs thought to trigger phage defense mechanisms (*18*, *20*); and group II intron-like 4 (G2L4) RTs, which evolved to function in double-strand break repair via microhomology-mediated end joining by optimizing the strong strand annealing activity of group II intron RTs (*22*). Further examples include human LINE-1 and other eukaryotic non-LTR retrotransposon RTs, which evolved variations of their reverse transcription and DNA integration mechanisms adapted to eukaryotic genomes and nuclear-cytoplasmic compartmentalization, and the core spliceosomal protein PRP8, which evolved from a group II intron-like RT and promotes RNA splicing by binding small nuclear RNAs derived from group II intron RNA domains (*72*).

The flexibility and advantageous biochemical activities of group II intron-related RTs may also be useful for biotechnological applications. In addition to transcriptional recording devices (*23*), group II intron and other non-LTR retroelement RTs that use multiple cDNA initiation mechanisms analogous to those found here for RT-Cas1 may be advantageous for genome engineering methods, such as prime editing (*73*–*76*). Such methods could potentially benefit from using RTs that synthesize full-length cDNAs without an added primer, as well as from the high fidelity, processivity, and strand-displacement activity that are enabled by or could be engineered into the distinctive conserved structural features of these RTs (*40*). Further, the finding that the addition of short DNA segments enables Mm RT-Cas1/Cas2 to site-specifically integrate RNA into a DNA genome suggests a general method for integration of RNA into genomes by other DNA integrases and recombinases.

## MATERIALS AND METHODS

### Bioinformatic analysis of spacers acquired in vivo

Spacer sequences were obtained from the Sequence Read Archive depository: Mm WT and RTΔ-Cas1/Cas2: SRP066108; Fs: SRR8102160, 163, 166-8, 170, 172-7, 194, 207; Vv: SRR8962131-134,137,138; Tt: SRR11818505-8, SRR12227011-4; Se: SRR16249482-3; St: SRR1595171-3, 176,187,198, 209. Raw reads were downloaded and merged if the dataset was composed of pair-end reads by using fastq-join (v1.3.1, 12/15/2015, https://github.com/brwnj/fastq-join). Spacers were identified as sequences between two repeats. Candidate spacers were mapped to the genome of the bacterial strain from which they were obtained [Mm: MMB-1 NC_015276.1; Fs: *E. coli* BL21 (DE3) NC_012947.1; Vv: *E. coli* HMS174 (DE3) NZ_LM993812.1; Tt: Tt phage phiFa/KO MH673671.2 and MH673672.2; Se: *Staphylococcus aureus* RN4220 NZ_CP076105.1; St: St JIM8232 FR875178.1] by using Bowtie2 (v2.2.5, 03/09/2015, https://github.com/BenLangmead/bowtie2) with the following parameters: --very-sensitive-local. After mapping, duplicate spacer sequences were removed by samtools (v1.12, 03/17/2021, https://github.com/samtools/samtools) using the parameter markdup -r. Spacers with unique sequences were then analyzed by using customized bash and R scripts to identify nonencoded nucleotides and nucleotide frequencies at different positions. Density plots for spacer length distribution were plotted in R (v4.3.1, 06/16/2023, https://r-project.org/).

The numbers of spacers having noncoded A, C, G, and T residues at positions N_1_ to N_5_ from the assumed 3′ end of the encoded spacer sequence (Fig. 1C) and dinucleotide frequencies at spacer-repeat junctions (fig. S1C) were calculated for acquired spacers with noncoded nucleotide at one end only. For fig. S1A, spacer sequences on opposite strands were rearranged so that all matched the sense-strand RNA sequence of the protein-coding or noncoding RNA gene to which they mapped, and the number of spacers with A, C, G, or T residues at the first five noncoded nucleotide positions was calculated and plotted as bar graphs in R. The distribution of spacer sequences across each gene was calculated as the percentile of the midpoint of each mapped spacer in the gene from which it originated (protein-coding or noncoding; host or viral; fig. S1D). Spacers that mapped between annotated genes were not included in this analysis.

### Bacterial strains

*E. coli* DH5α [F^−^ Φ80*lac*ZΔM15 Δ(*lac*ZYA-*arg*F) U169 *rec*A1 *end*A1 *hsd*R17 (r_K_^−^, m_K_^+^) *pho*A *sup*E44 λ^−^ *thi*-1 *gyr*A96 *rel*A1] was used for cloning, and *E. coli* Rosetta2 (DE3) [F^−^ *ompT hsdS*_B_(r_B_^−^ m_B_^−^) *gal dcm* (DE3) pRARE2 (Cam^R^), Novagen] was used for protein expression.

### DNA and RNA constructs used for protein expression and spacer acquisition assays

Mm WT RT-Cas1 and RT∆-Cas1 were expressed from plasmids pMalRF-RT-Cas1-cHis and pMalRF-RT∆-Cas1-cHis, respectively (*12*, *14*). The expressed proteins have a maltose-binding protein (MBP) tag fused to their N terminus via a noncleavable rigid linker to increase their solubility and stability in the absence of bound nucleic acids (*77*) and a C-terminal 8× His tag. The amino acid at position 2 of Cas6-RT-Cas1 was changed from leucine to valine to accommodate the MBP fusion.

pET9Cas2, used to express Cas2 protein without an added tag, was constructed by polymerase chain reaction (PCR) amplifying the Cas2 coding region using primers Cas2_pet_5 and Cas2_pet_3 from the plasmid pCassetteAv2_pBAD, which contains the *M. mediterranea* CRISPR03 array, RT-Cas1, and Cas2 cloned into the pBAD/Myc–His B backbone (Life Technologies) (*12*). The PCR product was then cloned between the Nde I and Bam HI sites of pET9a (EMD Millipore). The cloned PCR-amplified DNA sequences were confirmed by Sanger sequencing.

Plasmid CassetteAv2_pBAD (see above) was used to prepare an 88–base pair (bp) CRISPR DNA substrate (40-bp leader, 35-bp repeat 1, and 13 bp of spacer 1) by PCR with primers MMB1Lead40-5 and MMB1crisp3-r1 using Phusion High-Fidelity DNA polymerase according to the suppliers’ protocol (New England Biolabs or Thermo Fisher Scientific). The substrate was labeled by PCR with 20 μCi of [α-^32^P]-dCTP (3000 Ci/mmol, Revvity), 120 μM dCTP, and 600 μM of the other three dNTPs and purified by electrophoresis in a 6% polyacrylamide/8 M urea gel, cutting out the labeled band, and electro-eluting the DNA using midi D-Tube dialyzer cartridges (Novagen). The eluted DNA was concentrated by butanol extraction, purified by using a Zymo Oligo Clean and Concentrator kit, and quantitated against a [α-^32^P]-dCTP standard curve using a Beckman LS6500 liquid scintillation counter.

A shorter 68-bp CRISPR DNA was prepared by annealing complementary oligonucleotides L20R1top and L20R1bot, which have biotin moieties at their 3′ ends. Each oligonucleotide (10 μM) was mixed, heated to 82°C for 2 min, and slowly cooled to room temperature. The 68-bp CRISPR DNA contains a 20-bp leader, 35-bp repeat, and 13 bp of spacer 1. A hairpin variant (HP-3) of the 68-nt CRISPR DNA was made by connecting the 3′ end of the top strand to the 5′ end of the bottom strand by a 5-nt linker (5′-TACAT) and omitting the 3′ biotin blocking group. The sequences of all oligonucleotides used in this study are shown in table S1.

### Preparation of single-stranded and duplex protospacers used in spacer integration assays

Synthetic oligonucleotide protospacers were 5′-labeled in 50-μl reactions containing 1 nmol oligonucleotide, 600 μCi of [γ-^32^P]-ATP (6000 Ci/mmol; Revvity), 1 nmol ATP, and 40 U of T4 polynucleotide kinase (New England Biolabs) incubated for 45 min at 37°C in the manufacturer’s buffer and purified in a 10% polyacrylamide/8 M urea gel. The gel area containing the oligonucleotide was cut out, crushed, and incubated in 1 ml of TE [10 mM tris-HCl (pH 7.5) and 1 mM EDTA] per ~1.5 cm of gel slice at 4°C overnight on a rotator. The supernatant was removed, and the gel pieces were washed with an equal volume of TE. The combined supernatants were extracted repeatedly with butanol until the volume was ≤200 μl. After cleanup with a Zymo Oligo Clean and Concentrator kit, the labeled oligonucleotide was eluted in 40 to 60 μl of distilled H_2_O and quantitated against a [γ-^32^P]-ATP standard curve using an LS 6500 scintillation counter (Beckman-Coulter).

Duplex protospacers consisted of synthetic 5′-labeled RNA or DNA oligonucleotides having complementary 29- or 30-nucleotide sequences with dA tails that leave different length single-stranded 3′-dA overhangs on both strands. The oligonucleotides were annealed at 2.5 μM concentration each in 20 μl of 25 mM tris-HCl (pH 7.5), 20 mM NaCl by heating to 94°C for 30 s and slowly cooling (0.1°C/s) to 25°C followed by further incubation for 5 min at 25°C and then placed on ice before use. Analysis of the annealed protospacers on a nondenaturing 15% polyacrylamide gel indicated that >95% were double-stranded.

### Protein expression and purification

Protein expression plasmids were transformed into *E. coli* Rosetta2 (EMD Millipore) and plated on LB plates [lysogeny broth; 10 g of tryptone, 5 g of yeast extract, 10 g of NaCl, 10 mM tris-HCl (pH 7.5), and 20 g of agar (DIFCO) per liter of dH_2_O] containing ampicillin (100 μg/ml) and chloramphenicol (25 μg/ml). Single transformed colonies were inoculated into 100-ml LB medium supplemented with the same concentrations of ampicillin and chloramphenicol in a 250-ml Erlenmeyer flask and incubated overnight with shaking at 37°C. For preparation of Mm RT-Cas1 proteins, six 4-liter flasks with 1-liter LB broth were each inoculated with 10 ml of the overnight culture and grown at 37°C with shaking to log phase [optical density at 600 nm (OD_600_), 0.6 to 0.8]. IPTG (isopropyl β-d-1-thiogalactopyranoside) was then added to a final concentration of 1 mM, and the cultures were incubated at 19°C for 20 to 24 hours. Cells were harvested by centrifugation (Beckman-Coulter JLA-8.1 rotor, 20 min, 5000*g*), and the pelleted cells were resuspended in 10 ml/g cells of A1 buffer [25 mM tris-HCl (pH 7.5), 500 mM NaCl, 10% glycerol, and 10 mM ß-mercaptoethanol (BME)] on ice. The cells were then incubated with lysozyme (1 mg/ml, 30 min, 4°C) and sonicated (Branson Sonifier 450; three bursts of 15 s each with 15 s between each burst) in an ice water bath. The lysate was cleared by centrifugation (Beckman-Coulter JA-14 rotor; 29,400*g*, 25 min, 4°C), and polyethyleneimine was added to the supernatant with stirring on ice in six steps to a final concentration of 0.4%. After 10 min, precipitated nucleic acids were removed by centrifugation (JA-14 rotor; 29,400*g*, 25 min, 4°C), and proteins were precipitated from the supernatant by adding ammonium sulfate to 60% saturation and incubating on ice for 30 min. Proteins were collected by centrifugation (Beckman-Coulter JA-14 rotor; 29,400*g*, 25 min, 4°C), dissolved in 20 ml of A1 buffer, and filtered through a 0.45-μm polyethersulfone membrane (Whatman Puradisc).

Protein purifications were done by using an ÄKTA start system (Cytiva). RT-Cas1 proteins were purified by loading the filtered crude protein onto an amylose column (5 ml; MBPTrap HP, Cytiva), washing with 50 ml of A1 buffer, followed sequentially by 30 ml of A1 plus 1.5 M NaCl and 30 ml of A1 buffer. Bound proteins were eluted with 50 ml of 10 mM maltose in A1 buffer. Fractions containing RT-Cas1 were identified by SDS-PAGE and pooled. The protein was then loaded onto a Nickel column (5 ml, HisTrap FF column, Cytiva) and eluted with a 100-ml 25 to 100 mM imidazole gradient. Peak fractions were identified by SDS-PAGE, pooled, and dialyzed into A1 buffer (10-kDa molecular weight cutoff; SnakeSkin Dialysis Tubing; Thermo Fisher Scientific). The dialyzed protein was concentrated to >10 μM by using a 30K Pall concentrator and stored at −80°C in small (25 to 50 μl) aliquots.

The initial steps in the preparation of Cas2 expressed from pET9-Cas2 were similar to those for RT-Cas1, except that the cell paste was resuspended in H1 buffer [25 mM tris-HCl (pH 7.5), 100 mM KCl, 10% glycerol, and 10 mM dithiothreitol (DTT)] and precipitated by adding ammonium sulfate to 40% saturation. The Cas2-containing supernatant after ammonium sulfate precipitation was loaded directly onto a heparin-Sepharose column (5 ml; HiTrap Heparin HP column, Cytiva) and eluted with a linear 100 mM to 1 M KCl gradient. Cas2 peak fractions (~800 mM KCl) were identified by SDS-PAGE and frozen in elution buffer for storage overnight at −80°C. The peak fractions were thawed, diluted to ~125 mM KCl, and loaded onto a second 5-ml heparin-Sepharose column. Cas2 was eluted with a 100 to 400 mM KCl gradient, followed sequentially by extended washes with 400 mM KCl and an elution gradient from 400 mM to 1 M KCl to separate Cas2 from contaminating nucleases. Peak fractions were identified by SDS-PAGE, dialyzed against H1 buffer, and loaded onto an SP column (5 ml; HiTrap SP HP column; Cytiva) from which proteins were eluted with a 100 mM to 1 M KCl linear gradient. Peak fractions were identified by SDS-PAGE and stored at −80°C.

Protein concentrations were measured by using a Qubit Protein assay kit (Life Technologies) according to the manufacturer’s protocol. Nucleic acid contamination was assessed by using Qubit ssDNA, dsDNA, and ssRNA assay kits. ssDNA was below 1 ng/ml in purified protein preparations, and RNAs and dsDNA were undetectable. All proteins were >90% pure as assayed by SDS-PAGE.

### Terminal transferase assays

Purified RT-Cas1-8xHis protein (2 μM) was mixed with purified Cas2 (2 μM) in reaction medium containing 100 mM KCl, 100 mM NaCl, 25 mM tris-HCl (pH 7.5), 10 mM MgCl_2_, 1 mM DTT, 1 mM BME, and 2% glycerol and incubated at room temperature for 5 min before placing on ice before use. Assays were done in 20 μl of 25 mM tris-HCl (pH 7.5), 10 mM MgCl_2_, 5′-labeled DNA or RNA oligonucleotides (100 nM), and 1 mM of a single dNTP in equimolar MgCl_2_, and 1 mM MnCl_2_ was added where indicated. Reactions were initiated by adding 5 μl of protein complex for a final concentration of 500 nM. For time courses, the reaction volume was scaled up 10-fold and 20 μl of samples was taken at each time point (up to 2 hours). Terminal transferase assays with unlabeled oligonucleotide substrates were done as above in 20 μl of reaction medium containing 83 nM [α-^32^P]-dNTP (3000 Ci/mmol, Revvity) and 250 μM of unlabeled dNTP in equimolar MgCl_2_. Reactions were incubated at 37°C for 1 hour and stopped by adding phenol–chloroform–isoamyl alcohol (phenol-CIA, 25:24:1). The supernatant was mixed at a 2:1 ratio with loading dye [90% formamide, 20 mM EDTA, bromophenol blue (0.25 mg/ml), and xylene cyanole], and nucleic acids were analyzed on an 8% polyacrylamide/8 M urea gel, which was dried and scanned with a phosphorimager (Typhoon, Cytiva). Molecular weight markers were a 5′-labeled 10-nt ssDNA ladder (Invitrogen) run in a parallel lane.

### Spacer integration assays

Purified MBP-Cas6-RT-Cas1-8× His (2 μM) protein was mixed with purified Cas2 (2 μM) in reaction medium containing 100 mM KCl, 100 mM NaCl, 25 mM tris-HCl (pH 7.5), 10 mM MgCl_2_, 1 mM DTT, 1 mM BME, and 2% glycerol, incubated at room temperature for 5 min, and then placed on ice before use. Spacer integration reactions were done with RT-Cas1/Cas2 complex (500 nM), CRISPR DNA (5 to 100 nM), and single-strand or duplex DNA or RNA oligonucleotides (5 or 250 nM) in 50 mM KCl, 50 mM NaCl, 20 mM tris-HCl (pH 7.5), 10 mM MgCl_2_, and dNTPs (0.5 to 1 mM in equimolar MgCl_2_), and MnCl_2_ (1 mM) was added as indicated for individual experiments. The reaction volumes for the time courses were either 250 μl when taking 20-μl samples at each time point, 120 μl when taking 10-μl samples, or 20 μl for individual samples. Up to eight time courses were run in parallel in a PCR apparatus. Reactions were initiated by adding the RT-Cas1/Cas2 complex that had been preincubated with CRISPR DNA, incubating at 37°C for times up to 2 hours, and stopping the reaction by adding phenol-CIA (experiments in Fig. 2, A and B) or EDTA (25 mM) and 1.8 U of proteinase K and incubating at 25°C for 15 min (all other experiments). The supernatant was mixed at a 2:1 ratio with loading dye [90% formamide, 20 mM EDTA, bromophenol blue (0.25 mg/ml), and xylene cyanole], and nucleic acids were analyzed on a 6% polyacrylamide/8 M urea gel. Gels were dried and scanned with a phosphorimager. Molecular weight markers were a 5′-labeled 10-nt ssDNA ladder (Invitrogen) run in a parallel lane.

Time course gels for spacer acquisition assays using the hairpin CRISPR DNA were done at 30°C and quantitated by exposing the autoradiogram for times at which none of the bands were saturated after scanning. Gels were analyzed in ImageQuant (Cytiva) by boxing labeled bands along with a background control above or below the labeled band. The fraction of product was determined from the counts in the labeled band minus the background relative to the total counts of all labeled products in the lane. The data for the largest band (154 nt), which is an intermediate that has a protospacer inserted into the top strand, were fit to the equation *Y* = [(a*k1)/(*k*2 − *k*1)]*[exp(−*k*1*t) − exp(−k2*t)], where *t* = time and *k*1 and *k*2 are the rate constants for production and further reaction of the intermediate. The other two bands were fit to a single exponential equation. *R*^2^ values for all curve fits are shown in table S2.

### Reverse transcription assays

Reactions were done with Mm WT RT-Cas1/Cas2 protein (500 nM) and DNA or RNA template oligonucleotides (250 nM) in 10 or 20 μl of reaction medium [50 mM KCl, 50 mM NaCl, 25 mM tris-HCl (pH 7.5), and 10 mM MgCl_2_, with dNTPs added at 500 μM or 1 mM in equimolar MgCl_2_]. For the reverse transcription assays in Fig. 7A, dinucleotide primers were 5′-labeled using T4 polynucleotide kinase (New England Biolabs) and added at 20 μM final concentration without preannealing to the template. cDNA synthesis assays with unlabeled primers or without primers were done with a dNTP mix containing 83 nM [α-^32^P]-dNTP (3000 Ci/mmol, Revvity) and 20 μM of the same unlabeled nucleotide plus 500 μM of the other three dNTPs or 83 nM [α-^32^P]-dNTP (3000 Ci/mmol) plus 500 μM of each dNTP, as specified for individual experiments. MnCl_2_ was added where indicated at 1 mM final concentration. Reactions were incubated at 25°C for 1 hour and stopped by adding EDTA (25 mM) plus 1.8 U of protease K (New England Biolabs) and incubating at 25°C for 15 or 30 min. The samples were mixed at a 2:1 ratio with loading dye [90% formamide, 20 mM EDTA, bromophenol blue (0.25 mg/ml), and xylene cyanole] and analyzed in a 20% polyacrylamide/8 M urea gel, which was dried and scanned with a phosphorimager. Molecular weight markers were a 5′-labeled 10-nt ssDNA ladder (Invitrogen) run in a parallel lane. Time courses were done as above for times up to 90 min while scaling up the sample volume 10-fold and taking 10 or 20 μl of samples at each time point.

To determine whether RT-Cas1/Cas2 produces a stable RNA/cDNA duplex, cDNA synthesis reactions in the absence of primer were done as described above using the R50CCC-ddC or D50CCC_ddC templates. The reactions were scaled up to 40 μl and stopped by adding EDTA (25 mM). The cDNA products were then purified by using a Zymo Oligo Clean and Concentrator kit and incubated for 20 min at 37°C in the presence or absence of RNase H (5 U, New England Biolabs) or RNase A (2 μg, New England Biolabs) according to the manufacturer’s protocol followed by protease K digestion as above. Samples were analyzed in a native 15% polyacrylamide gel, which was dried and analyzed with a phosphorimager.

### Protein-priming assays

Purified RT-Cas1 protein (2 μM) was mixed with purified Cas2 (2 μM) in reaction medium containing 100 mM KCl, 100 mM NaCl, 25 mM tris-HCl (pH 7.5), 10 mM MgCl_2_, 1 mM DTT, 1 mM BME, and 2% glycerol and incubated at room temperature for 5 min before placing on ice until ready for use. Protein-priming assays were done with 500 nM RT-Cas1/Cas2 in the absence or presence of 250 nM DNA or RNA oligonucleotide templates in reaction medium containing 25 mM tris-HCl (pH 7.5) and 10 mM MgCl_2_, with 1 mM MnCl_2_ added where indicated. Reactions were initiated by adding 83 nM [α-^32^P]-dNTP (3000 Ci/mmol; Revvity) in 25 mM tris-HCl (pH 7.5) and 10 mM MgCl_2_ and incubated at 25°C for 15 min. Some reactions were chased by adding 500 μM of a single dNTP or all four dNTPs in 25 mM tris-HCl (pH 7.5), 10 mM MgCl_2_ buffer, or 25 mM tris-HCl (pH 7.5) and 10 mM MgCl_2_ buffer alone as a control followed by further incubation at 25°C for 30 min. Some samples were treated with protease K (1.8 U; New England Biolabs), MNase (4 × 10^5^ U in 1 × MNase buffer, New England Biolabs), or RNase A (2 μg; Monarch RNase A, New England Biolabs) and incubated for another 15 min at 25°C. The reactions were stopped by adding 4× NuPAGE Sample Buffer (Invitrogen) or 4× Laemmli Buffer (Bio-Rad) to a final 1× concentration and heating to 90°C for 3 min. Samples were analyzed on a 4 to 15% polyacrylamide NuPAGE bis-tris gel (Thermo Fisher Scientific) in 1× morpholineethanesulfonic acid/SDS running buffer (Thermo Fisher Scientific) or on a 4 to 20% polyacrylamide Criterion gel (Bio-Rad) in 1× tris/glycine/SDS buffer (Bio-Rad) with Precision Plus Protein standards (Bio-Rad) as size markers. Protein gels were stained with Coomassie blue as described (*78*), dried, and scanned with a phosphorimager.

### Coupled reverse transcription–spacer acquisition assays for RNase H digestion of duplex spacers

cDNAs were synthesized by incubating 5′-labeled R29 + dA2 or R29 + dA2_ddC protospacers (250 nM) with RT-Cas1/Cas2 (500 nM) and dNTPs (100 μM) in 40 μl of 50 mM KCl, 50 mM NaCl, 25 mM tris-HCl (pH 7.5), and 10 mM MgCl_2_ in the presence or absence of MnCl_2_ (30 μM) and a dinucleotide dG_2_ or dT_2_ primer (20 μM) for 1 hour at 25°C. Relatively low concentrations of MnCl_2_ and dNTPs were used to limit the RT-Cas1 terminal transferase activity. The samples were cleaned up with a Zymo Oligo Clean and Concentrator kit and eluted in 40 μl of H_2_O. Spacer acquisition assays were done as above by incubating 5 μl of the purified protospacer with Mm RT-Cas1/Cas2 (500 nM) and CRISPR hairpin DNA substrate (100 nM) for 1 hour at 25°C. After adding EDTA to 25 mM, the reaction products were treated with protease K (0.32 U, 15 min, 37°C) and cleaned up with a Zymo Oligo Clean and Concentration kit. The products were then eluted in 25 μl of H_2_O, and 10 μl was incubated with or without RNase H (12.5 U; New England Biolabs) according to the manufacturer’s protocol for 15 min at 37°C. Samples were mixed at a 2:1 ratio with loading dye and analyzed on an 8% polyacrylamide/8 M urea gel against a 5′-labeled New England Biolabs low–molecular weight DNA ladder run in a parallel lane.

### TGIRT-seq of cDNAs synthesized by Mm RT-Cas1/Cas2

Mm RT-Cas1/Cas2 reverse transcription reactions used to synthesize cDNAs for sequencing were done as above with 500 nM RT-Cas1/Cas2, 250 nM RNA template, and 500 μM dNTPs without or with 1 mM MnCl_2_ or 20 μM dinucleotide DNA primers in 160 μl of reaction medium (see above) for 1 hour at 25°C. The reaction was stopped by adding 20 μl of stop solution [20 mM tris-HCl (pH 7.5) and 200 mM EDTA] with or without RNase A (0.8 μg/μl) as indicated and incubated for 15 min at 37°C followed by digestion with protease K (20 μl, 0.4 U/μl) for 15 min at 37°C. The products were cleaned up with a Zymo Oligo Clean & Concentrator Kit. Nucleic acid concentrations were measured with a Qubit ssDNA Assay kit (Invitrogen) according to the manufacturer’s protocols, and 10 to 20 ng of cDNA product from each reaction were used for TGIRT sequencing (TGIRT-seq) library preparation.

For construction of TGIRT-seq libraries for identification of cDNA start sites, first-strand DNA synthesis was done by TGIRT template switching using a 34-nt RNA containing an Illumina R2 adapter sequence annealed to a complementary 35-nt R2 (R2R) DNA leaving a single-nucleotide 3′ DNA overhang (an equimolar mixture of A, T, G, and C) that promotes template switching by base pairing to the 3′ nucleotide of an acceptor nucleic acid, in this case, a cDNA synthesized by RT-Cas1/Cas2 (*79*, *80*). The reaction was stopped by adding protease K (1 U; New England Biolabs) and 25 mM EDTA followed by clean-up with a Monarch PCR & DNA Cleanup Kit (New England Biolabs). Second-strand DNA synthesis was done by ligating 10 μM of a blunt-end duplex composed of a 32-bp Illumina R1-3′ SpC3 and 5′-phosphorylated R1R-3′SpC3 DNA (preannealed by incubating at 95°C for 3 min and slowly cooling to 25°C) using a Quick ligase kit (New England Biolabs) according to the manufacturer’s protocol followed by clean-up as described above. The ligated dsDNA products were amplified by PCR using Phusion High-Fidelity PCR Master Mix with HF Buffer (New England Biolabs) with 200 nM of Illumina multiplex and index barcode primers (98°C for 10 s before denaturation followed by 15 cycles of 98°C for 5 s, 60°C 10 s, 72°C 15 s). The resulting cDNA libraries were cleaned up by using 1.4× AMPure XP beads (Beckman Coulter) and eluted in 25-μl H_2_O. One microliter of the library was analyzed on a 2100 Bioanalyzer using a High Sensitivity DNA chip (Agilent) to assess product profiles and concentrations, and the remainder was sequenced on an Illumina Nextseq 550 to obtain ~1 million 2 × 75–nt paired-end reads per sample at the Genome Sequencing and Analysis Facility (GSAF) at the University of Texas at Austin.

For read mapping, Illumina TruSeq adapters and PCR primer sequences were trimmed from the reads with Cutadapt (v3.5, 09/29/2021, https://github.com/marcelm/cutadapt) sequencing quality score cutoff at 20; *P* < 0.01), and reads <15 nt after trimming were discarded. Pair-ended reads were merged by using fastq-join. The merged reads were then mapped to the reverse complement of the corresponding template sequences using Bowtie2 with the parameters --very-sensitive-local -L 5. Mapped reads with five or fewer soft-clipped nucleotides were retrieved and realigned to the reverse complement of the template sequence using MAFFT (multiple alignment using fast Fourier transform, v7.520, 22 March 2023, https://github.com/GSLBiotech/mafft) with the following settings: --auto --addfragments --reorder --keeplength --preservecase. Mapping of cDNA start sites onto the template sequence, stacked bar graphs, and sequence logos of reads comprising ≥1% of all reads were made using R.

For analysis of snap-back DNA synthesis products, reads containing the full-length template sequence linked to the 5′ end of a cDNA were collected and RNA template sequences were trimmed by using Cutadapt v3.5 with default settings. Trimmed reads were then mapped to the reverse complement of the corresponding template sequences using Bowtie2 with the parameters --very-sensitive-local -L 5. Mapped reads were retrieved and realigned to the reverse complement of the template sequence by using MAFFT with the following settings: --auto --addfragments --reorder --keeplength --preservecase. Reads for snap-back DNA synthesis products comprising ≥5% of all mapped reads were plotted against the corresponding template sequences using Illustrator (Adobe). All sequencing datasets are listed in table S3.

### Quantitation and statistical analysis

Products of terminal transferase, spacer acquisition, and cDNA synthesis assays were quantitated using ImageQuant TL version 8.1 (Cytiva). Excel version 16 (Microsoft) was used to determine mean, median, and SD values. Prism 10.0 (GraphPad Software) was used for curve fitting of quantitated assays to determine *k*_obs_, amplitude, and *R*^2^ values for the curve fits. R (v4.0.3) package ggplot2 was used to generate sequence logos from high-throughput sequencing data.
