# Supplementary material for: Mechanisms used for cDNA synthesis and site-specific integration of RNA into DNA genomes by a reverse transcriptase–Cas1 fusion protein
Source: Sci Adv. 2024 Apr 12;10(15):eadk8791. doi: 10.1126/sciadv.adk8791 (PMC11014452; doi:10.1126/sciadv.adk8791)
Supplement: Supplementary file 1 — Figs. S1 to S17 Tables S1 to S4 [file sciadv.adk8791_sm.pdf]

Science Advances
AAAS

Supplementary Materials for

Mechanisms used for cDNA synthesis and site-specific integration of RNA into DNA genomes by a reverse transcriptase–Cas1 fusion protein

Georg Mohr *et al.*

Corresponding author: Alan M. Lambowitz, lambowitz@austin.utexas.edu

Sci. Adv. **10**, eadk8791 (2024)
DOI: 10.1126/sciadv.adk8791

This PDF file includes:

Figs. S1 to S17
Tables S1 to S4

Science Advances
AAAS

Supplementary Materials for

Mechanisms used for cDNA synthesis and site-specific integration of RNA into DNA genomes by a reverse transcriptase-Cas1 fusion protein

Georg Mohr *et al.*

*Corresponding author. Email: lambowitz@austin.utexas.edu

This PDF file includes:

Fig. S1 to S17
Table S1 to S4

Fig. S1.

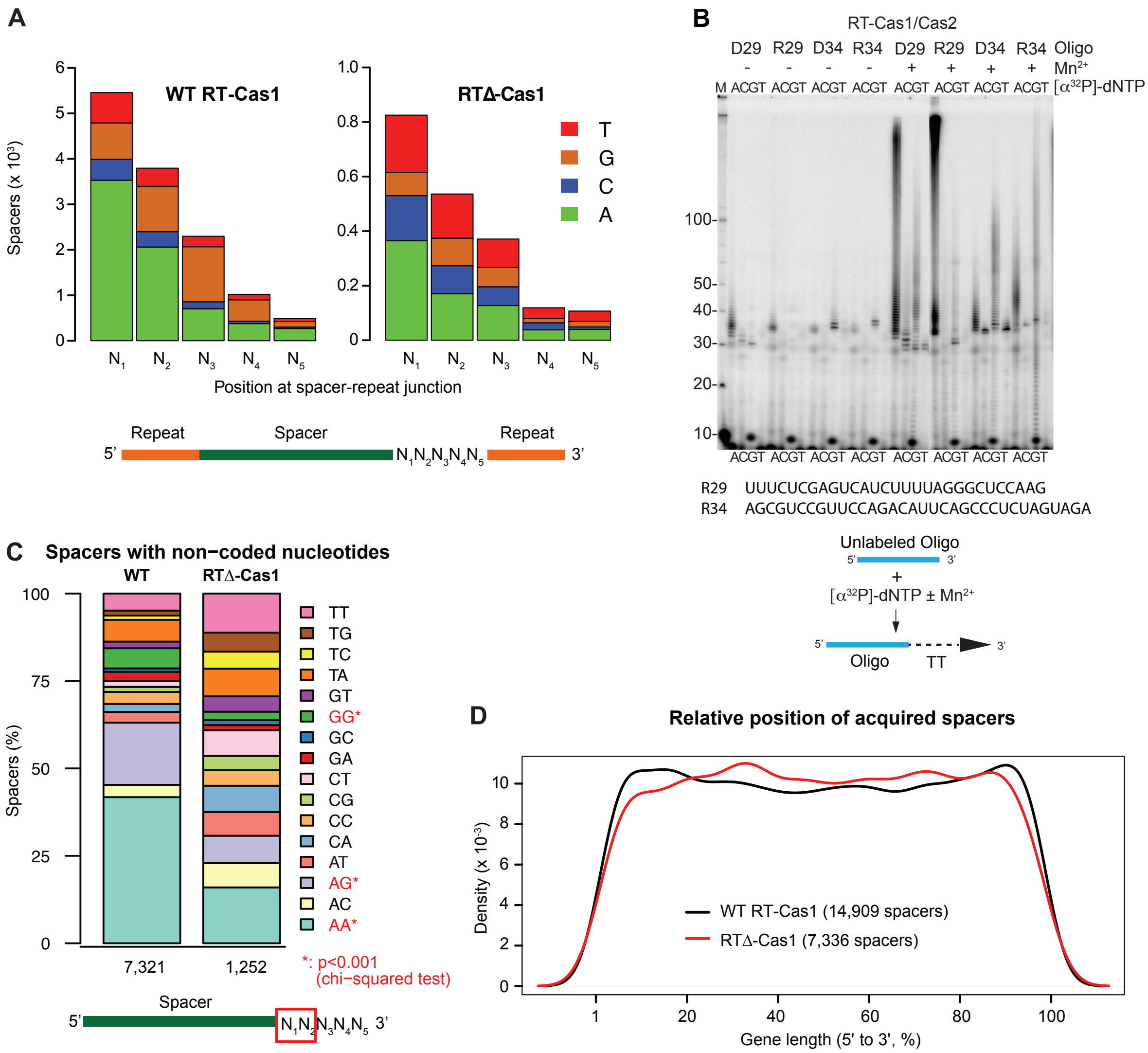
A
WT RT-Cas1
RTΔ-Cas1
Spacers (x 10³)
T
G
C
A
N1 N2 N3 N4 N5
Position at spacer-repeat junction
Repeat
Spacer
Repeat
5'
N1N2N3N4N5
3'
B
RT-Cas1/Cas2
D29 R29 D34 R34 D29 R29 D34 R34 Oligo
- - - - + + + + Mn2+
M ACGT ACGT ACGT ACGT ACGT ACGT ACGT ACGT [α32P]-dNTP
100-
50-
40-
30-
20-
10-
R29 UUUCUCGAGUCAUCUUUUAGGGCUCCAAG
R34 AGCGUCCGUUCCAGACAUUCAGCCCUCUAGUAGA
Unlabeled Oligo
+
[α32P]-dNTP ± Mn2+
Oligo
TT
C
Spacers with non−coded nucleotides
WT
RTΔ-Cas1
Spacers (%)
TT
TG
TC
TA
GT
GG*
GC
GA
CT
CG
CC
CA
AT
AG*
AC
AA*
7,321
1,252
*: p<0.001
(chi−squared test)
Spacer
D
Relative position of acquired spacers
Density (x 10⁻³)
WT RT-Cas1 (14,909 spacers)
RTΔ-Cas1 (7,336 spacers)
Gene length (5' to 3', %)

Fig. S1. Characteristics of spacers integrated by Mm WT RT-Cas1/Cas2 and RTΔ-Cas1/Cas2 *in vivo* and additional RT-Cas1/Cas2 terminal transferase assays. (**A**) Analysis of non-coded nucleotides at the 3' end of the RNA sense strand of spacers inserted into the top or bottom strand of the CRISPR array by Mm WT RT-Cas1/Cas2 or RTΔ-Cas1/Cas2. The stacked bar graphs show the number of spacers acquired by Mm WT RT-Cas1/Cas2 and RTΔ-Cas1/Cas2 with non-coded A, C, G, or T residues at positions N_1 to N_5 at the 3' end of the RNA sequence. (**B**) Terminal transferase assays done as in Fig. 2 for 1 h at 37˚C, but with unlabeled DNA or RNA oligonucleotide substrates and each of the 4 possible [α-^{32}P]-dNTPs. Quantitation of the gel is shown in fig. S15. D29 and R29 are the same 29-nt DNA and RNA oligonucleotides used for terminal transferase assays in Fig. 2, and D34 and R34 are 34-nt RNA and DNA oligonucleotides whose sequence corresponds to a randomly chosen spacer acquired by Mm RT-Cas1/Cas2 *in vivo*. The sequences of R29 and R34 and a schematic of the terminal transferase (TT) assay are shown below the gel. A repeat of the experiment using 5' labeled oligonucleotides and unlabeled dNTPs and incubating for 1 h at 37˚C gave similar results (fig. S8). (**C**) Percentages of different non-coded dinucleotides at positions N_1 and N_2 at spacer-repeat junctions for spacers with non-coded nucleotides at only one end, assumed to be the RNA 3' end. Those dinucleotides whose percentages at positions N_1 and N_2 were significantly higher for Mm WT RT-Cas1/Cas2 than RTΔ-Cas1/Cas2 by chi-squared test ($p<0.01$) are highlighted in red and marked with an asterisk (*). (**D**) Distribution of spacer sequences acquired by WT RT-Cas1/Cas2 and RTΔ-Cas1/Cas2 within the genes from which they originated. The analysis was done using the same spacer datasets as in Fig. 1B with spacers mapped to the *M. mediterranea* genome. The locations of the spacer sequences were calculated as the percentile of the mid-point of the acquired sequence. Spacers derived from both coding and non-coding RNAs were used for this analysis. The plot shows a probability density function (y-axis) plotted for the relative position of each spacer within the gene to which it was mapped (x axis), with 1 and 100% corresponding to the 5' and 3' ends of the sense-strand RNA sequence, respectively.

Fig. S2.

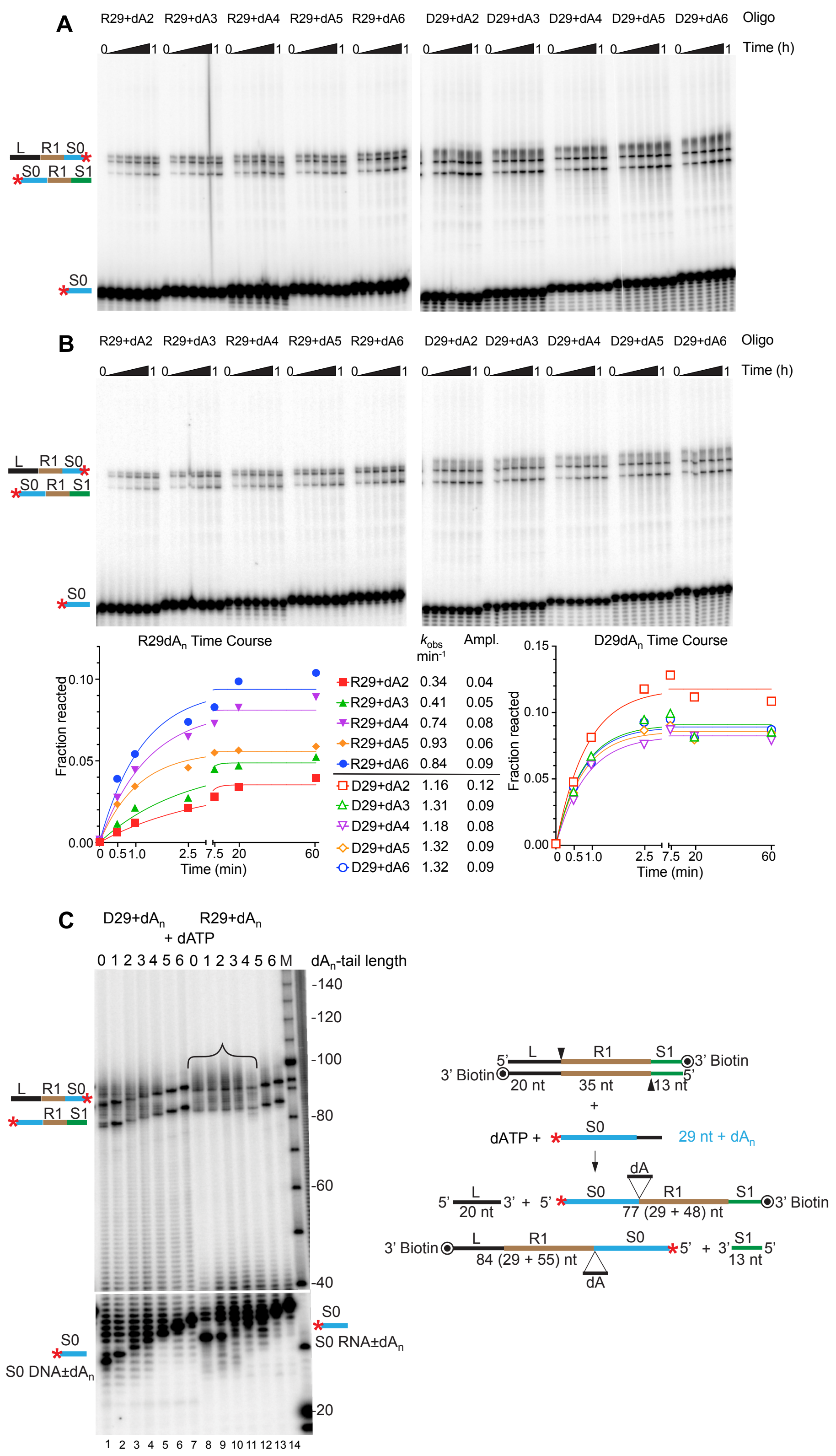

A
R29+dA2 R29+dA3 R29+dA4 R29+dA5 R29+dA6 D29+dA2 D29+dA3 D29+dA4 D29+dA5 D29+dA6 Oligo
Time (h)
L R1 S0
S0 R1 S1
S0
B
R29+dA2 R29+dA3 R29+dA4 R29+dA5 R29+dA6 D29+dA2 D29+dA3 D29+dA4 D29+dA5 D29+dA6 Oligo
Time (h)
R29dAn Time Course
D29dAn Time Course
Fraction reacted
Time (min)
kobs min-1 Ampl.
R29+dA2 0.34 0.04
R29+dA3 0.41 0.05
R29+dA4 0.74 0.08
R29+dA5 0.93 0.06
R29+dA6 0.84 0.09
D29+dA2 1.16 0.12
D29+dA3 1.31 0.09
D29+dA4 1.18 0.08
D29+dA5 1.32 0.09
D29+dA6 1.32 0.09
C
D29+dAn R29+dAn
+ dATP
0 1 2 3 4 5 6 0 1 2 3 4 5 6 M
dAn-tail length
-140 -120 -100 -80 -60 -40 -20
S0 DNA±dAn
S0 RNA±dAn
1 2 3 4 5 6 7 8 9 10 11 12 13 14
5' L R1 S1 3' Biotin
3' Biotin 20 nt 35 nt 13 nt 5'
dATP + S0 29 nt + dAn
5' L 3' + 5' S0 dA R1 S1 3' Biotin
20 nt 77 (29 + 48) nt
3' Biotin L R1 S0 5' + 3' S1 5'
84 (29 + 55) nt dA 13 nt

Fig. S2. Additional data for spacer acquisition assays with RNA and DNA oligonucleotides having different length 3'-dA tails. (**A** and **B**) Phosphorimager scans of gels for which time courses are shown in Fig. 3C (panel A) and phosphorimager scans and plots for a repeat of those time courses (panel B). The phosphorimager scans were analyzed with ImageQuant TL software, and the data were fit to a single-exponential equation using Prism to calculate k_{obs} and Amplitude (Ampl.) for the sum of the 2 indicated discrete bands corresponding to ligated protospacers. R^2 values for the plots are listed in table S2. Plots including the heterogenous label above the 2 discrete bands in some gels gave similar curves with higher rates and amplitudes (fig. S10). (**C**) Spacer acquisition assays for the same D29 and R29 oligonucleotides with different length dA tails analyzed in Fig. 3B in the presence of 1 mM dATP to allow further extension of dA tails. A schematic of the assay is shown to the right. Optimal integration of shorter RNA but not DNA protospacers into the CRISPR array required extension to 33 nt by Mm RT-Cas1/Cas2 terminal transferase addition of more 3'-dA residues (bracket in gel). Reactions were done in parallel with and analyzed on the same gel as those without added dATP in Fig. 3B. Full sets of gels including time courses for R29+A1 and D29+A1 consistent with results for a fixed time point in Fig. 3B (barely detectable integration for R29+A1 and efficient integration for D29+A1) are shown in fig. S10.

Fig. S3.

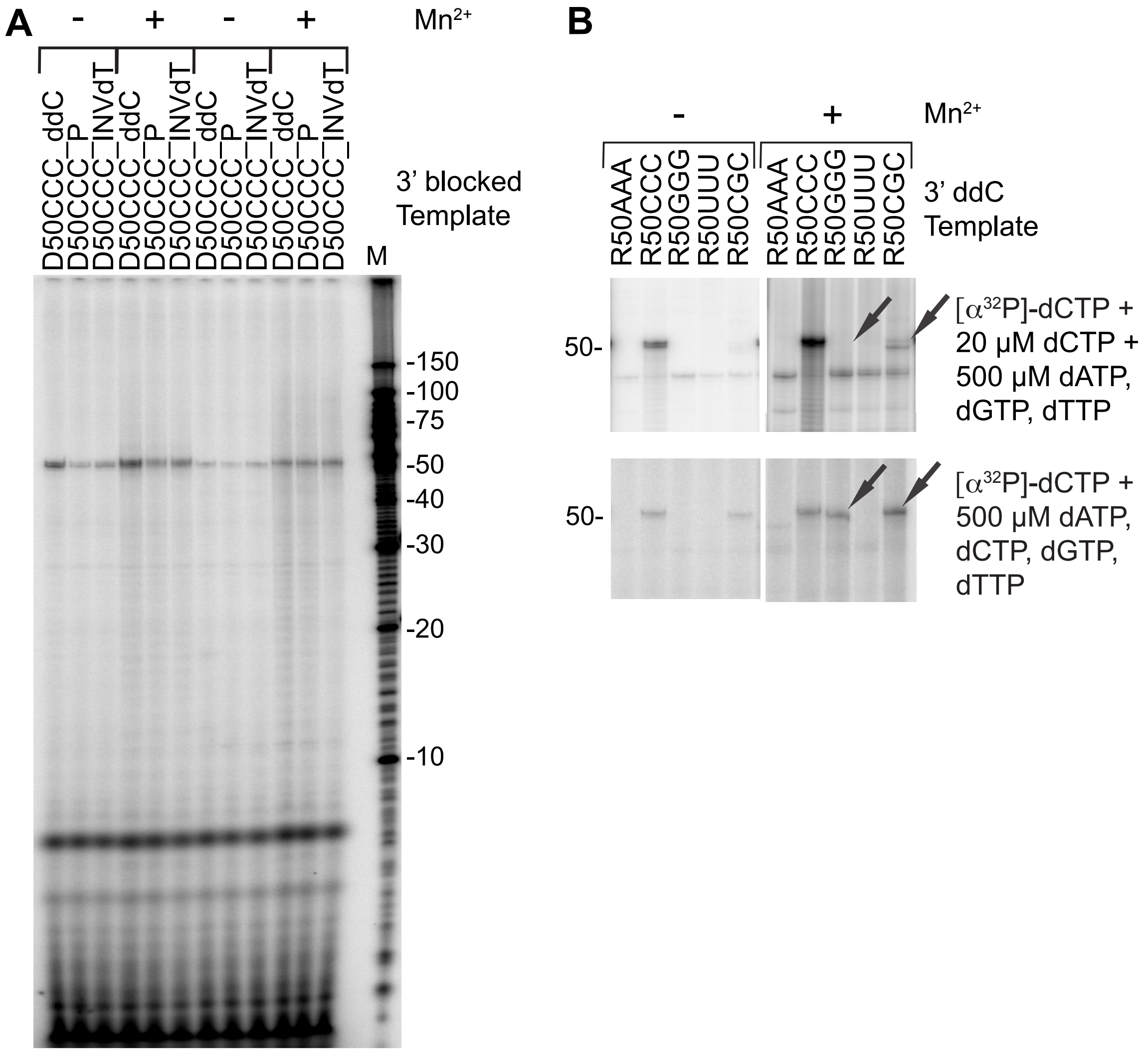
A
- + - + Mn2+
D50CCC_ddC
D50CCC_P
D50CCC_INVdT
3' blocked Template
M
-150
-100
-75
-50
-40
-30
-20
-10
B
- + Mn2+
R50AAA
R50CCC
R50GGG
R50UUU
R50CGC
3' ddC Template
50-
[α32P]-dCTP + 20 µM dCTP + 500 µM dATP, dGTP, dTTP
[α32P]-dCTP + 500 µM dATP, dCTP, dGTP, dTTP

Fig. S3. Gel analysis of labeled cDNAs synthesized by Mm RT-Cas1/Cas2 from different templates without an added primer. (**A**) cDNA synthesis from D50CCC and R50CCC templates with different 3'-blocking groups. Reactions were done by incubating Mm WT RT-Cas1/Cas2 (500 nM) with D50CCC or R50CCC templates (250 nM) having different 3'-blocking groups (dideoxy C (ddC), phosphate (P) or an Inverted dT (INVdT)) and ^{32}P-labeled dNTPs (83 nM [α-^{32}P]-dCTP plus an equimolar mix of 500 μM dATP, dCTP, dGTP, and dTTP) in reaction medium containing 10 mM $MgCl_2$ ± 1 mM $MnCl_2$ for 1 h at 25˚C. The products were analyzed in a denaturing 20% polyacrylamide gel against a homemade ladder of 5'-labeled oligonucleotides in a parallel lane (M). The uniform size of the products synthesized from RNA and DNA templates with a 3'-phosphate or other 3'-blocking groups indicates that initiation of cDNA synthesis at 3'-proximal sites is not dependent upon a 3' OH. A comparison in parallel for all 3'-blocking groups was done once with similar results for all 3'-blocking groups. (**B**) Gel analysis of cDNAs synthesized from R50NNN templates containing a 3'-proximal trinucleotide sequence with lower or higher dCTP concentrations in the ^{32}P-dNTP mix. Reactions were done as described in Materials & Methods by incubating RT-Cas1/Cas2 (500 nM) with R50NNN_3'-ddC templates (250 nM) having AAA, CCC, GGG, UUU or CGC at the 3'-proximal trinucleotide site (nucleotides 46 to 48) in reaction medium containing 10 mM $MgCl_2$ ± 1 mM $MnCl_2$ for 1 h at 25˚C. In the top panels, reactions were done with trace (83 nM) [α-^{32}P]-dCTP plus 20 μM dCTP and 500 μM dATP, dGTP, and dTTP, and in the bottom panels, reactions were done with trace (83 nM) [α-^{32}P]-dCTP plus 500 μM dATP, dCTP, dGTP, and dTTP. The products were analyzed in a 20% polyacrylamide/8 M urea gel against a 5' labeled 10-nt ssDNA ladder. Black arrows indicate cDNAs initiated at the GGG and CGC sequences in the R50GGG and R50CGC templates at high dCTP concentration (bottom panel) but barely or not detectably at low dCTP concentration (top panel). The products were analyzed on two gels with lanes in the Figure rearranged to facilitate comparisons. The full gel for the top panels is shown in fig. S16, and the full gel for the bottom panels with all size markers is shown in Fig. 7B. cDNA initiation sites in the trinucleotide sequence were confirmed by TGIRT sequencing (Fig. 8B).

Fig. S4.

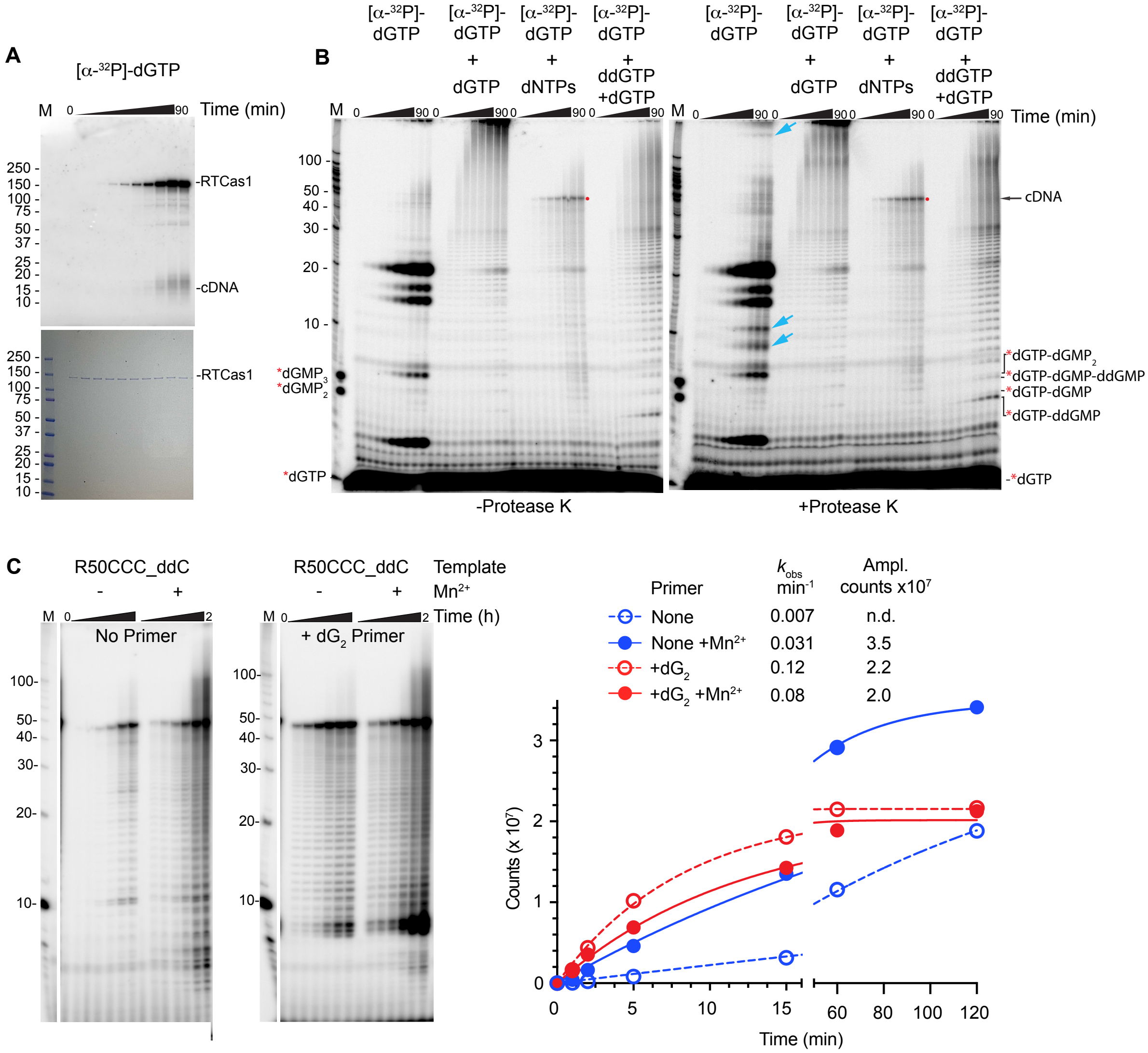
A
[α-32P]-dGTP
M
0
90
Time (min)
250 -
150 -
100 -
75 -
50 -
37 -
25 -
20 -
15 -
10 -
-RTCas1
-cDNA
-RTCas1
B
[α-32P]-dGTP
[α-32P]-dGTP + dGTP
[α-32P]-dGTP + dNTPs
[α-32P]-dGTP + ddGTP +dGTP
100 -
50 -
40 -
30 -
20 -
10 -
*dGMP3
*dGMP2
*dGTP
-Protease K
+Protease K
cDNA
*dGTP-dGMP2
*dGTP-dGMP-ddGMP
*dGTP-dGMP
*dGTP-ddGMP
*dGTP
C
R50CCC_ddC
Template
Mn2+
Time (h)
No Primer
+ dG2 Primer
100-
50-
40-
30-
20-
10-
Primer
kobs min-1
Ampl. counts x107
None
0.007
n.d.
None +Mn2+
0.031
3.5
+dG2
0.12
2.2
+dG2 +Mn2+
0.08
2.0
Counts (x 107)
Time (min)
0
5
10
15
60
80
100
120
1
2
3

Fig. S4. Time courses for protein priming and synthesis of labeled cDNAs and short DNA oligomers synthesized by Mm RT-Cas1/Cas2 from R50CCC_ddC template in the absence of an added primer and comparison of rates of cDNA synthesis from the same template in the absence or presence of a dinucleotide dG_2 primer. (**A**) Time course for labeling of RT-Cas1 with [α-^{32}P]-dGTP. Reactions were done as in Fig. 6 with Mm WT RT-Cas1/Cas2 (500 nM), R50CCC_ddC template (250 nM), and [α-^{32}P]-dGTP (83 nM) in reaction medium containing 10 mM $MgCl_2$ and 1 mM $MnCl_2$ at 25˚C. Samples were taken from 0 to 90 min, and the reactions stopped with EDTA (25 mM). An aliquot of each sample was analyzed on an SDS 4-20% polyacrylamide gradient gel against Precision Plus Marker (BioRad; lane M). A phosphorimager scan of the gel is shown above, and the Coomassie blue-stained gel is shown below. Cas2 is present in too low concentrations to be visible in the Coomassie blue-stained gel. (**B**) Time courses for labeling of RT-Cas1 and synthesis of DNA products. Four different reactions were done as in Fig. 6 with Mm WT RT-Cas1/Cas2 (500 nM) and R50CCC_ddC template (250 nM) in reaction medium containing 10 mM $MgCl_2$ and 1 mM $MnCl_2$ at 25˚C. The 4 reactions contained [α-^{32}P]-dGTP (83 nM), [α-^{32}P]-dGTP (83 nM) + 500 µM dGTP, [α-^{32}P]-dGTP (83 nM) + all 4 dNTPs (500 µM each), or [α-^{32}P]-dGTP (83 nM) + 500 µM dGTP and 1 mM ddGTP. Aliquots of the samples were incubated with or without Protease K and analyzed on a 20% polyacrylamide/8 M urea gel. Size markers were a homemade mixture of ^{32}P-labeled oligonucleotides and $dGMP_2$ and $dGMP_3$ in parallel lanes (M). Labeled RT-Cas1 remained in the wells unless samples were digested with Protease K to yield smaller labeled protein fragments (blue arrows) or chased with a higher concentration of dNTPs. (**C**) Rates of cDNA synthesis from the R50CCC_ddC template with or without added dinucleotide (dG_2) primer. RT-Cas1/Cas2 (500 nM) was incubated with 3'-blocked R50CCC template (100 nM) in the presence of 20 µM [α-^{32}P]-dCTP plus 500 µM dATP, dGTP, and dTTP for the indicated times at 25˚C in the presence or absence of 20 µM dG_2 primer in reaction medium containing 10 mM $MgCl_2$ ± 1 mM $MnCl_2$. The products were analyzed in a 20% polyacrylamide/8 M urea gel (left). The plots to the right show the rate of synthesis of the ~50-nt cDNA product quantitated with ImageQuant and fit to a single exponential rate equation using Prism 10. Rates (k_{obs}) and amplitudes (Ampl.) calculated from the curve fits are shown above the plots. R^2 values for the plots are listed in table S2. The full-time course in panels A-C were done once to confirm progressive labeling and to determine quantitative rates for multiple experiments done at fixed time points (*e.g.,* Fig. 4A, Fig. 6 A and D, and Fig. 7A).

Fig. S5.

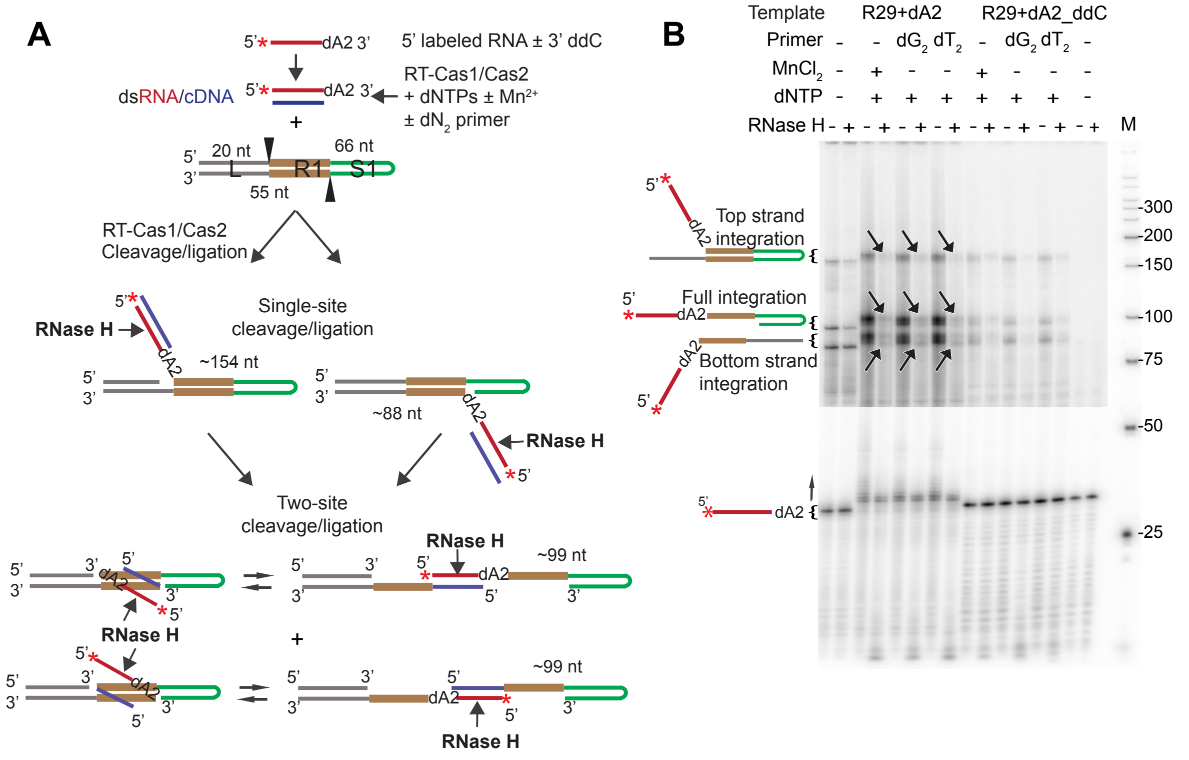
A
5' labeled RNA ± 3' ddC
dsRNA/cDNA
RT-Cas1/Cas2
+ dNTPs ± Mn2+
± dN2 primer
20 nt
66 nt
55 nt
L
R1
S1
RT-Cas1/Cas2
Cleavage/ligation
Single-site
cleavage/ligation
RNase H
~154 nt
~88 nt
Two-site
cleavage/ligation
~99 nt
B
Template
R29+dA2
R29+dA2_ddC
Primer
MnCl2
dNTP
RNase H
M
Top strand
integration
Full integration
Bottom strand
integration
-300
-200
-150
-100
-75
-50
-25

Fig. S5. RNase H control confirming integration of a stable RNA-dA/cDNA duplex into a CRISPR array. (**A**) Schematic of the experiment. cDNAs were synthesized by incubating 5'-labeled *R29+dA_2 or *R29+dA_2_ddC protospacers (250 nM) with RT-Cas1/Cas2 (500 nM) and dNTPs (100 µM) in reaction medium containing 10 mM $MgCl_2$ in the presence or absence of 1 mM $MnCl_2$ (30 µM) and a dinucleotide dG_2 or dT_2 primer (20 µM) for 1 h at 25˚C. After clean-up with a Zymo Oligo Clean and Concentrator kit, the products were incubated with Mm RT-Cas1/Cas2 (500 nM) and CRISPR hairpin DNA substrate (100 nM) for 1 h at 25˚C. EDTA was added to a final concentration of 25 mM, and the reaction products were treated with Protease K (0.32 units, 15 min, 37˚C), cleaned up with a Zymo Oligo Clean and Concentration kit, incubated with or without RNase H (12.5 units; New England Biolabs) for 15 min at 37˚C, and analyzed in an 8% polyacrylamide/8 M urea gel against a 5'-labeled New England Biolabs low molecular weight DNA ladder run in a parallel lane (M). (**B**) Phosphorimager scans of the dried gel with different exposures for the top and bottom of the gel to better visualize the residual 5'-labeled RNA template/protospacer that was not digested by RNase H. Arrows point to reaction products that showed substantially lower labeling after RNase H treatment, indicating that they were RNA-dN/cDNA duplexes that had stably integrated into the CRISPR array as shown in the schematic. The lighter product bands for the 3'-blocked R29+dA2_ddC RNA template/ protospacer, which should be unable to integrate into the CRISPR DNA, were not seen in the absence of dNTPs, suggesting that they reflect addition of 3'-dA tails required for integration to a fraction of the RNA protospacers that lacked or lost the 3'-ddC blocking group. The experiment was repeated with similar results (fig. S17).

Fig. S6.

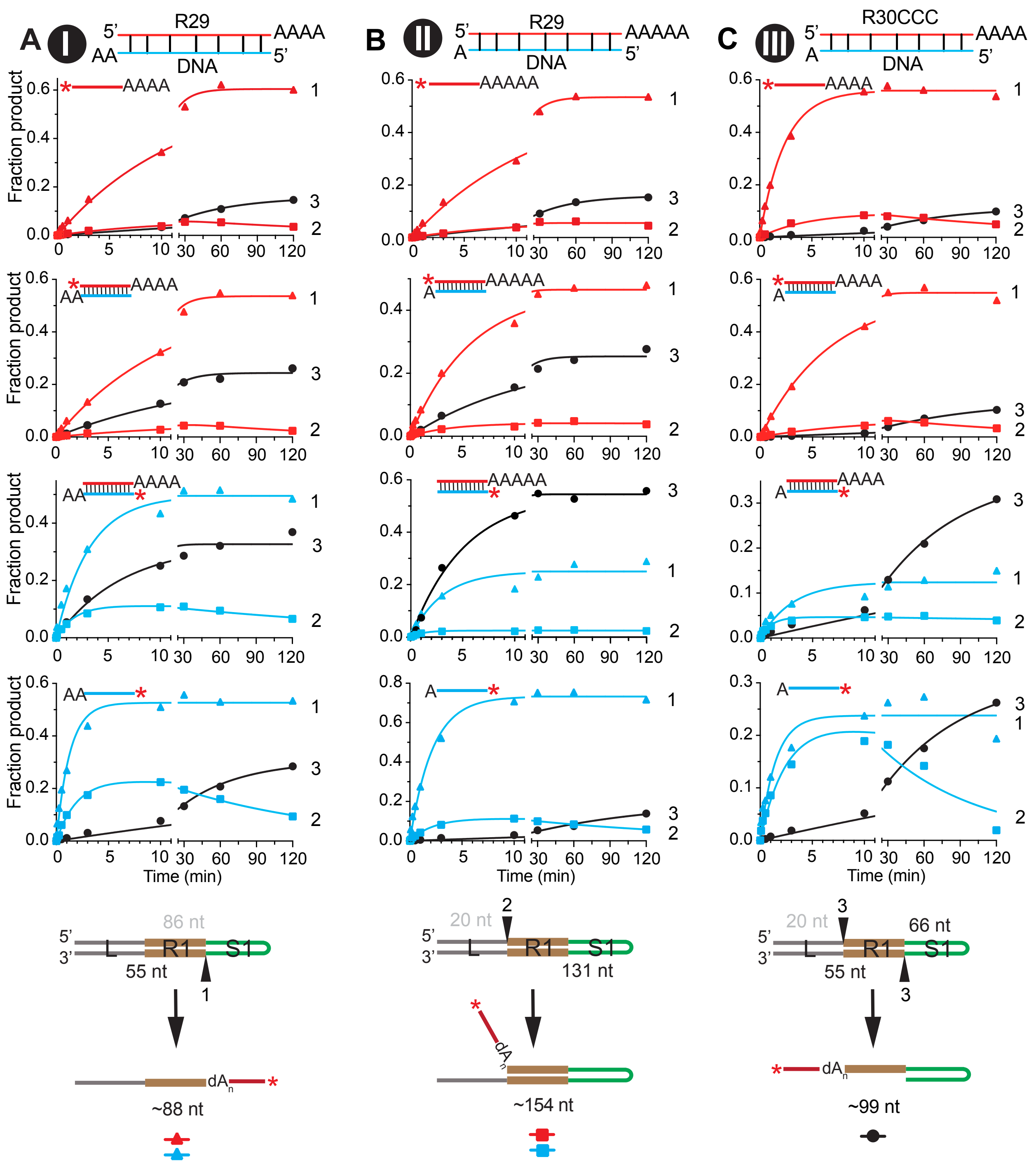

A
B
C
R29
R30CCC
DNA
Fraction product
Time (min)
86 nt
55 nt
20 nt
131 nt
66 nt
~88 nt
~154 nt
~99 nt

Fig. S6. Time courses for all ^{32}P-labeled products resulting from cleavage-ligation reactions at one site or both sites on the top and bottom strands of the CRISPR DNA in the protospacer integration assays of Fig. 9B. (**A-C**) The three different sets (I, II, and III) of ^{32}P-labeled (*) protospacers used in the experiment are shown schematically at the top of each panel. For each set of protospacer, assays were done with a single-stranded *RNA-dA_n, an *RNA-dA_n/DNA duplex, an RNA-dA_n/*DNA duplex, and a single-stranded *DNA. The plots for each product are symbol and color coded as shown at the bottom of the Figure, with numbers 1, 2, and 3 corresponding to the sites in the schematics (numbered black arrowheads) at which cleavage occurred to generate the product. Gray numbers in the schematics indicate the lengths of fragments that were unlabeled and thus were not visible in autoradiograms of the gels (fig. S14). In all cases, cleavage-ligation at the bottom-strand site (#1, triangles) occurred more rapidly than cleavage-ligation at the top-strand site (#2, squares). For the protospacers in panels A and B, cleavage-ligation reactions at both sites (#3, black circles) occurred faster for the duplex protospacers than for the single-stranded protospacers, indicating that the duplex configuration enabled more rapid sequential ligation into both strands of the CRISPR array.

Fig. S7.

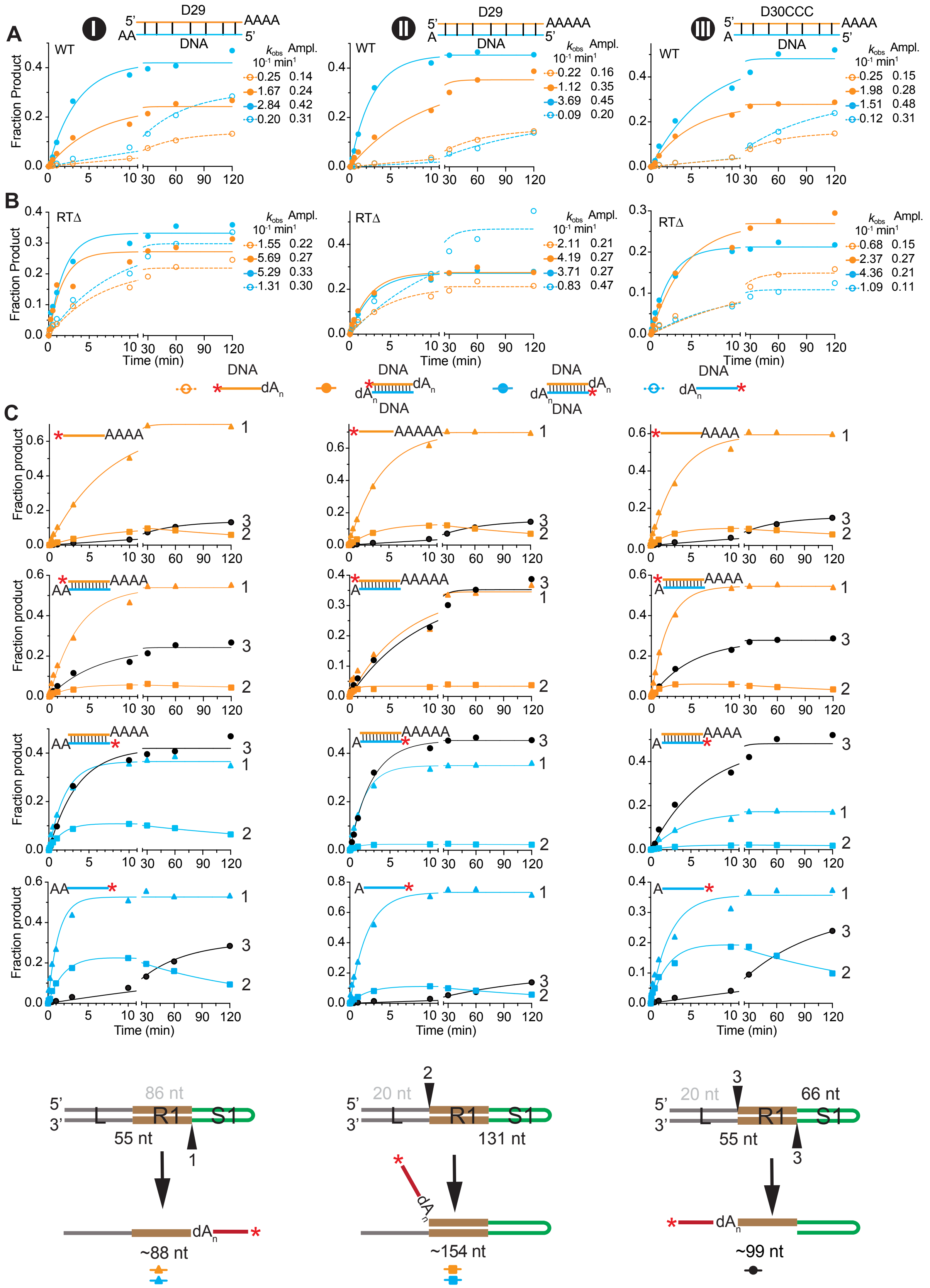

A
B
C
I
II
III
D29
D30CCC
5'
AA
AAAA
AAAAA
DNA
WT
RTΔ
Fraction Product
Fraction product
Time (min)
k_{obs} Ampl.
10^{-1} min^{-1}
0.25 0.14
1.67 0.24
2.84 0.42
0.20 0.31
0.22 0.16
1.12 0.35
3.69 0.45
0.09 0.20
0.25 0.15
1.98 0.28
1.51 0.48
0.12 0.31
1.55 0.22
5.69 0.27
5.29 0.33
1.31 0.30
2.11 0.21
4.19 0.27
3.71 0.27
0.83 0.47
0.68 0.15
2.37 0.27
4.36 0.21
1.09 0.11
dAn
86 nt
55 nt
20 nt
131 nt
66 nt
L
R1
S1
3'
~88 nt
~154 nt
~99 nt

Fig. S7. Time courses showing rates of integration of single-stranded and duplex DNA protospacers having the same sequences and overhang configurations as those for 3'-dA-tailed RNA and RNA/DNA duplex protospacers in Fig. 9, B and C. Spacer-integration reactions by Mm WT-RT-Cas1/Cas2 (panel A) and RTΔ-Cas1/Cas2 for 3 sets of all DNA protospacers (I, II, and III) with the same sequences and overhang configurations as RNA-dA and RNA-dA duplex protospacers in Fig. 9. The plots show time courses for production of the labeled 99-nt product resulting from cleavage-ligation reactions at the 5' end of R1 on both strands and are symbol and color coded as shown below panel B. The data were fit to a single exponential equation to calculate rates (k_{obs}) and amplitudes (Ampl.). * indicates the 5'-labeled strand. R^2 values for curve fits are shown in table S2. (**C**) Time courses for production of all ^{32}P-labeled products resulting from cleavage-ligation reactions at single or both sites on the top and bottom strands for the same reactions as panel A. The plots for each product are symbol and color coded as shown at the bottom of the Figure, with numbers 1, 2, and 3 corresponding to the sites in the schematics (numbered black arrowheads) at which cleavage occurred to generate the product. Gray numbers in the schematics indicate the lengths of fragments that were unlabeled and thus not visible in autoradiograms of the gels (fig. S14). The time courses were done once in parallel with those for the three similarly configured RNA-dA_n, ssDNA, and RNA-dA_n/DNA duplex protospacers in Fig. 9, B and C, with conclusions based on trends for RNA-dA_n versus all DNA protospacers.

Fig. S8. Repeat experiments for Fig. 2 and fig. S1B.

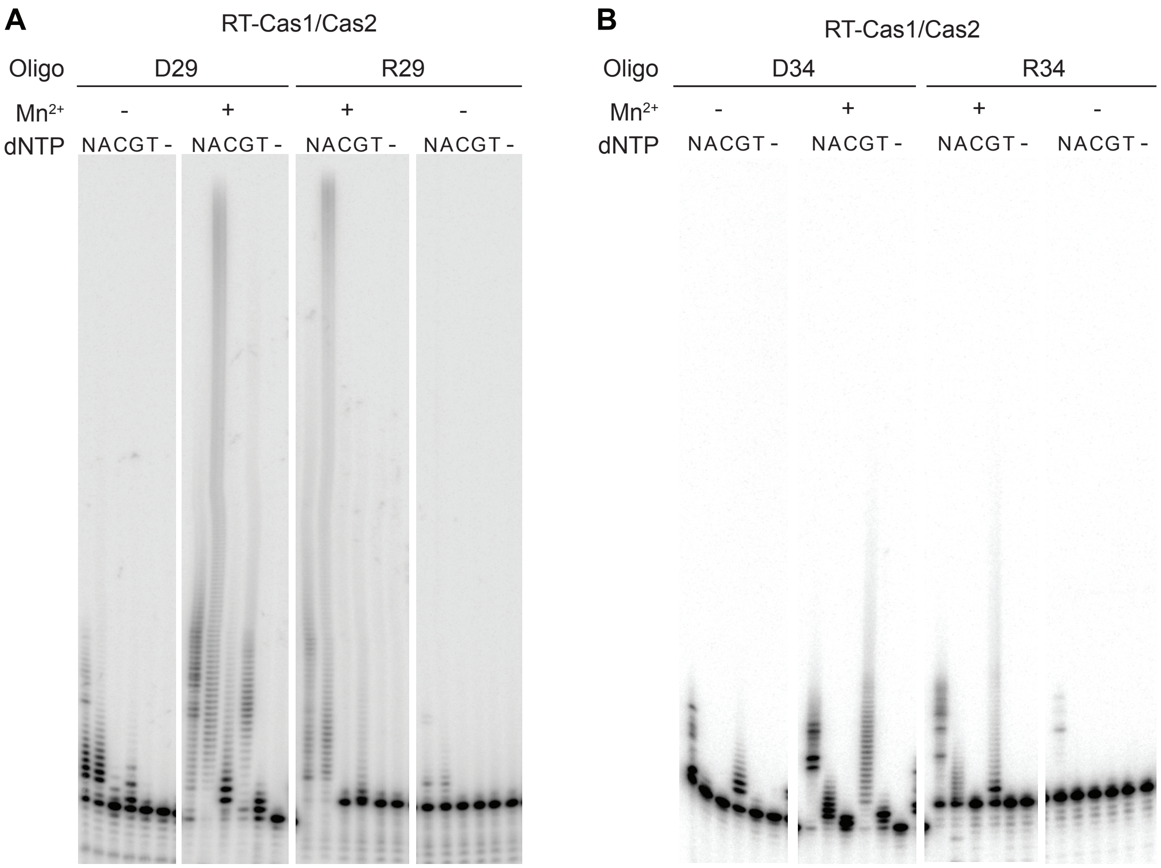

Fig. S9. Repeat experiments for Fig. 3A using a different CRISPR DNA.

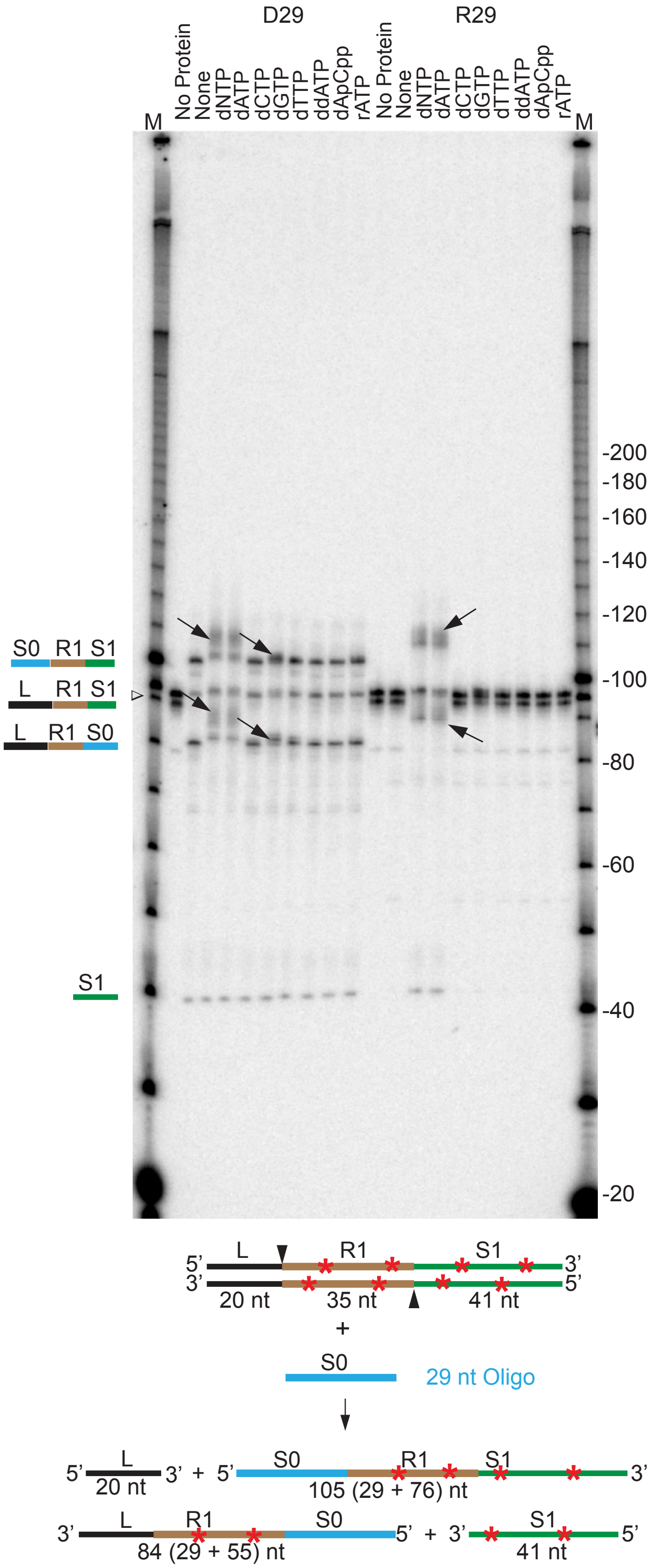

Fig. S10. Full gels for Fig. 3, fig. S2 A and B and alternative quantitation using all labeled product bands. n.d., not determined.

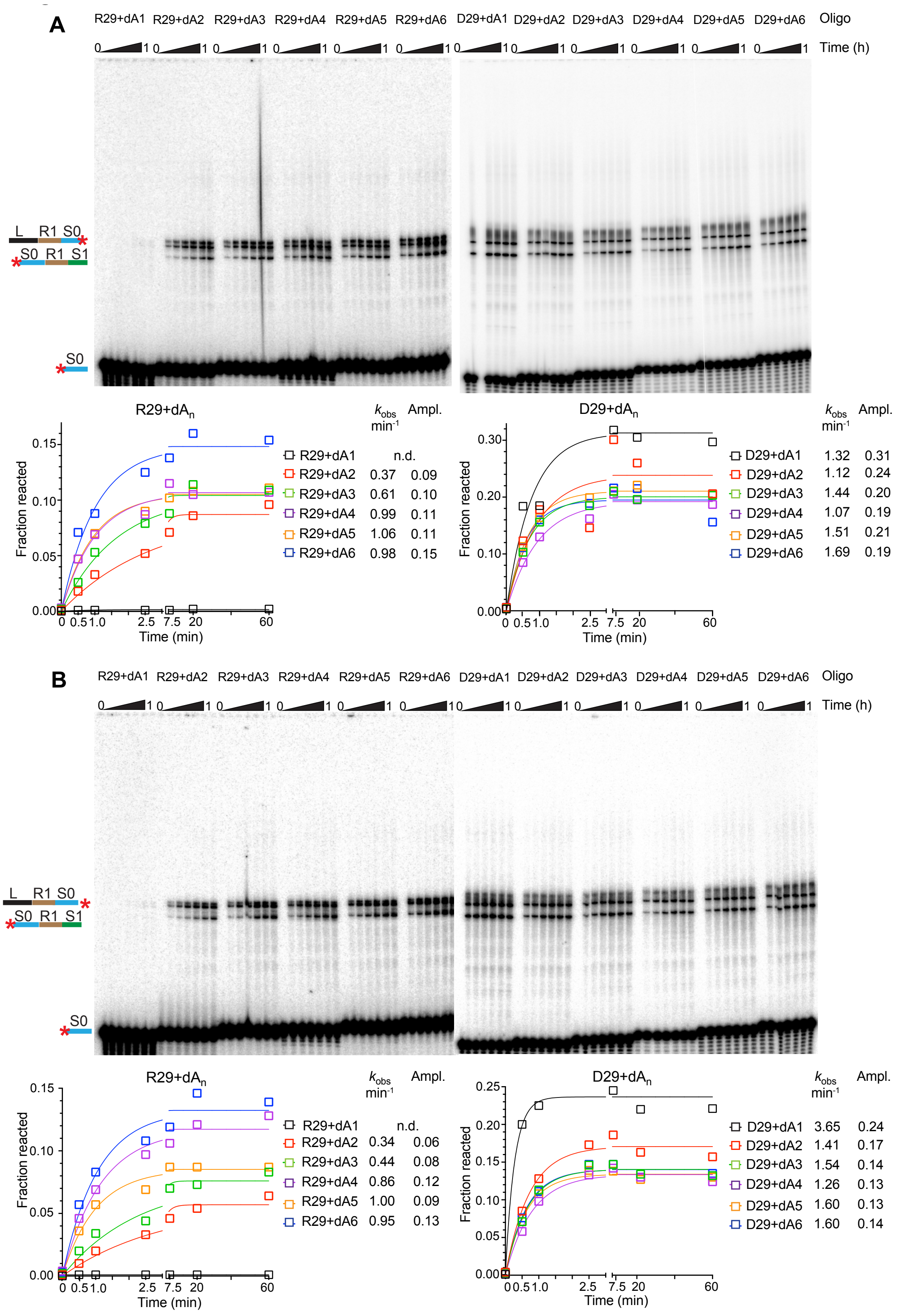

Fig. S11. Repeat experiments for Fig. 4.

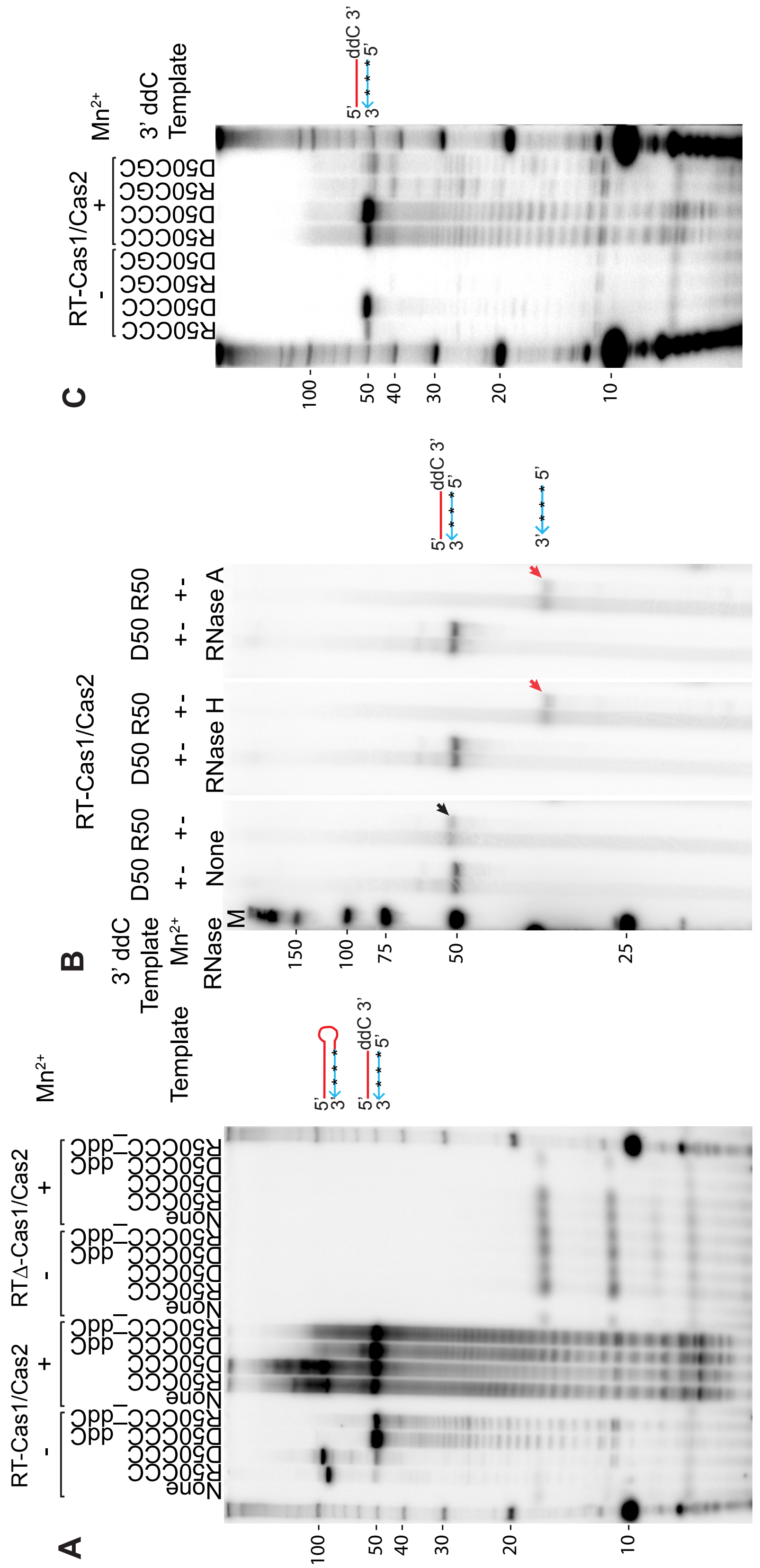

Fig. S12. Repeat experiments for Fig. 6. (**A**) Repeat of Fig. 6A, (**B**) Repeat of Fig. 6B, (**C**) Repeat of Fig. 6D.

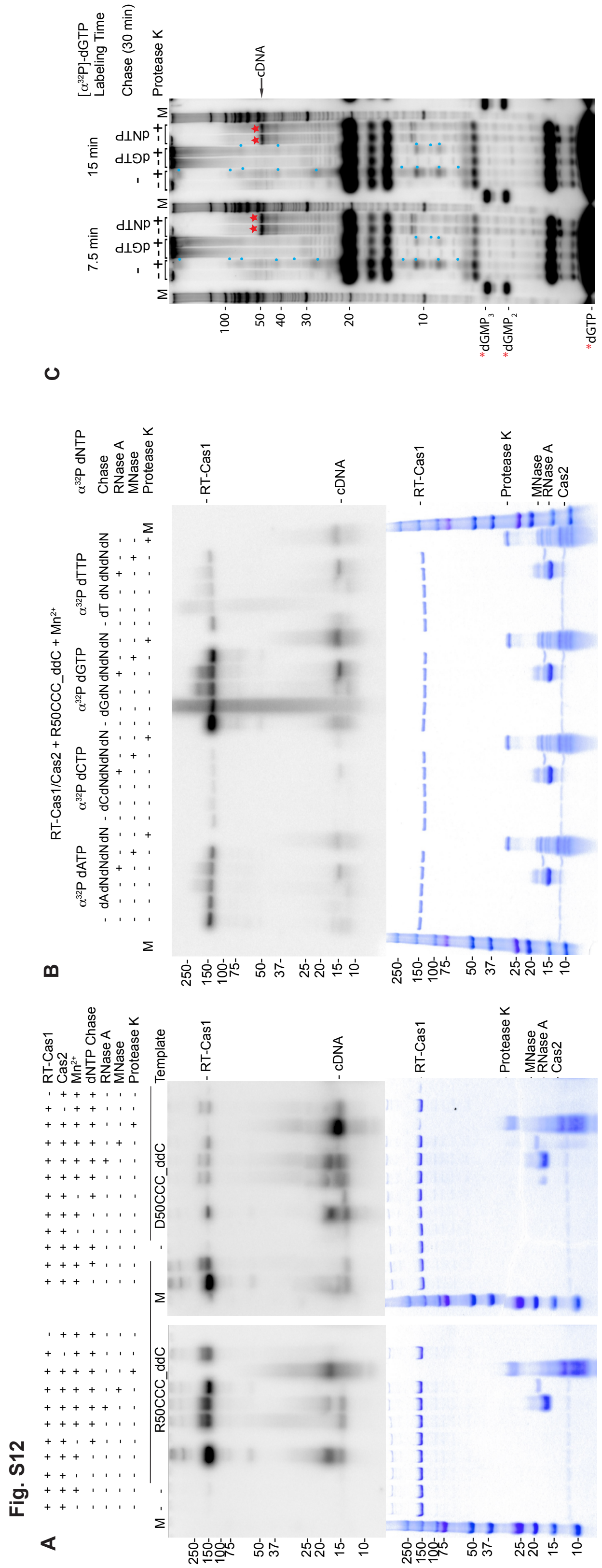

Fig. S13. Repeat experiments for Fig. 7.

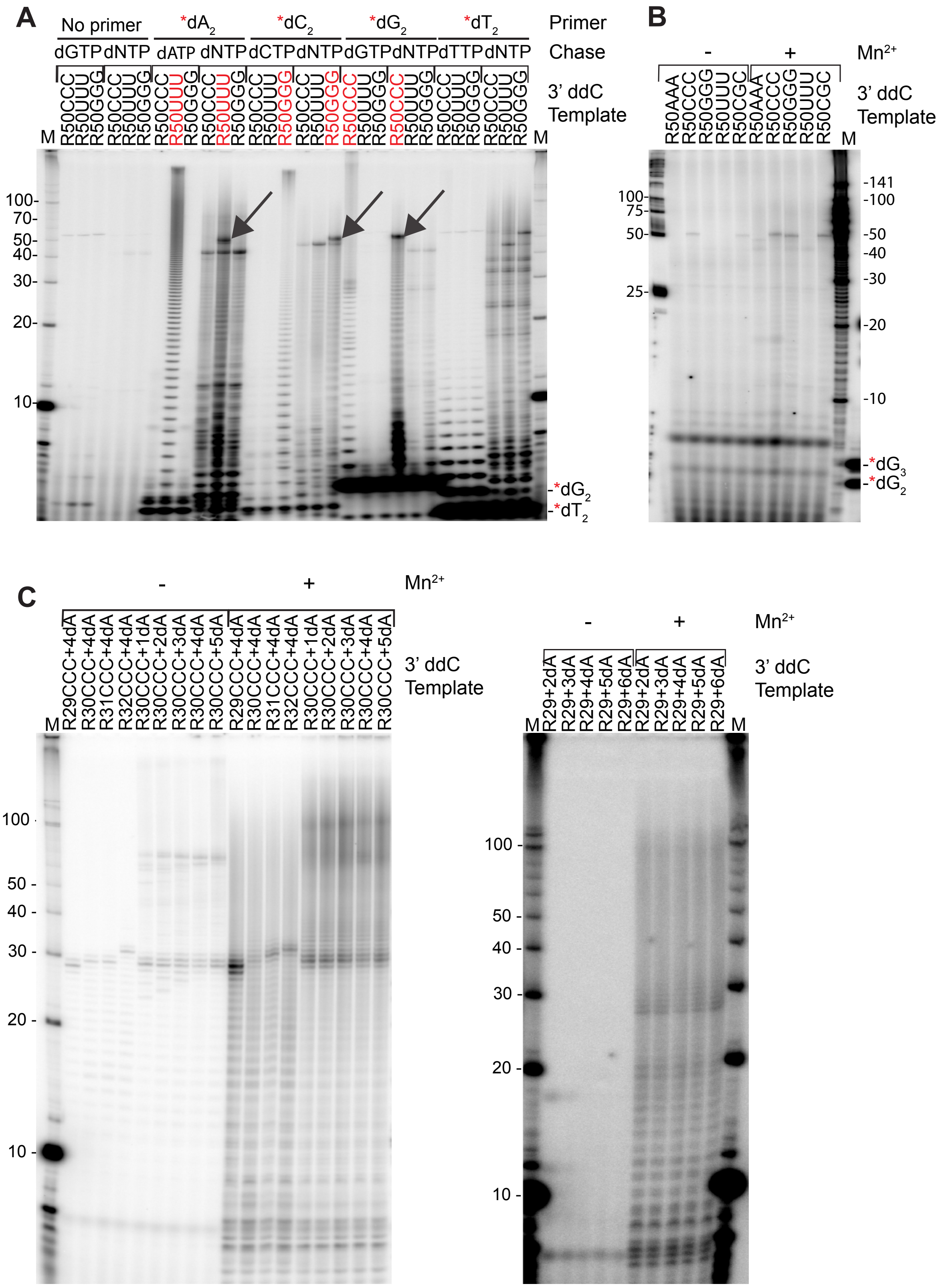

Fig. S14. Gels for spacer acquisition time courses. Gels for quantitation of spacer acquisition time courses. (**A**) Gels for Figure 9B and S6. (**B**) Gels for Figure 9C. (**C**) Gels for Figure 9D. (**D**) Gels for Figure S7A and S7C. (E) Gels for Figure S7B and S7C.

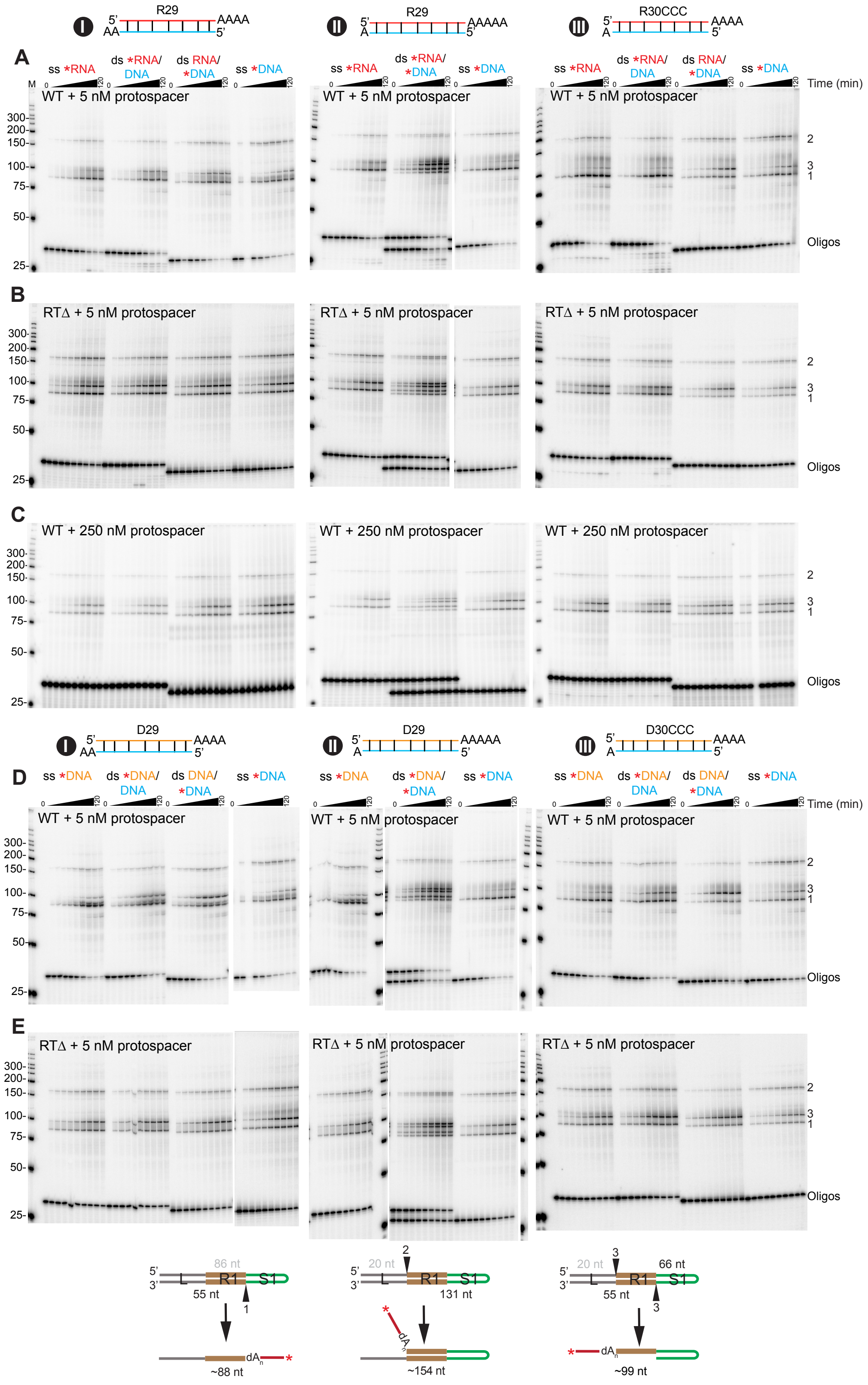

Fig. S15. Quantitation of fig. S1B. Counts for each lane (starting from the position of the 30 nt marker up to the well) were determined with ImageQuant. The total counts from each [α-^{32}P]-dNTP labeled oligonucleotide were divided by the total counts from the [α-^{32}P]-dATP reaction for the same oligonucleotide. The data were plotted in Prism.

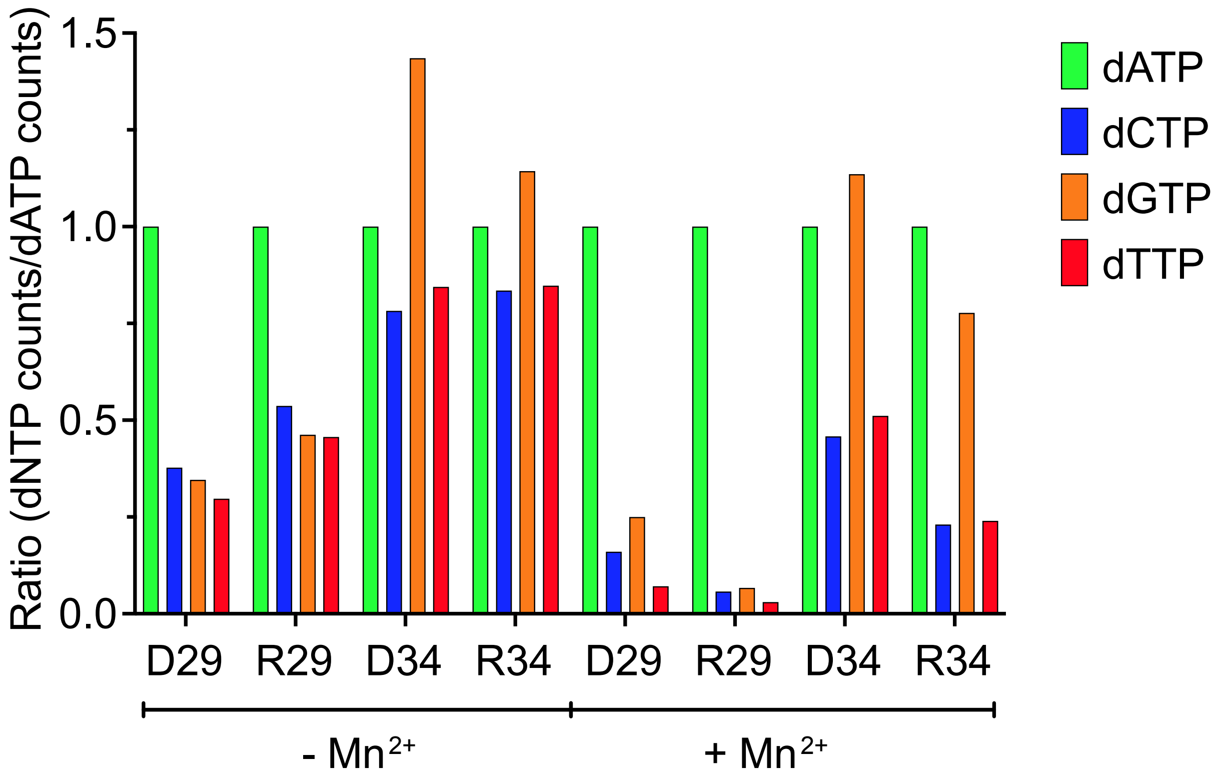

Fig. S16. Full gels for fig. S3B. (**A**) Gel for fig. S3B top. (**B**) Gel for fig. S3B bottom.

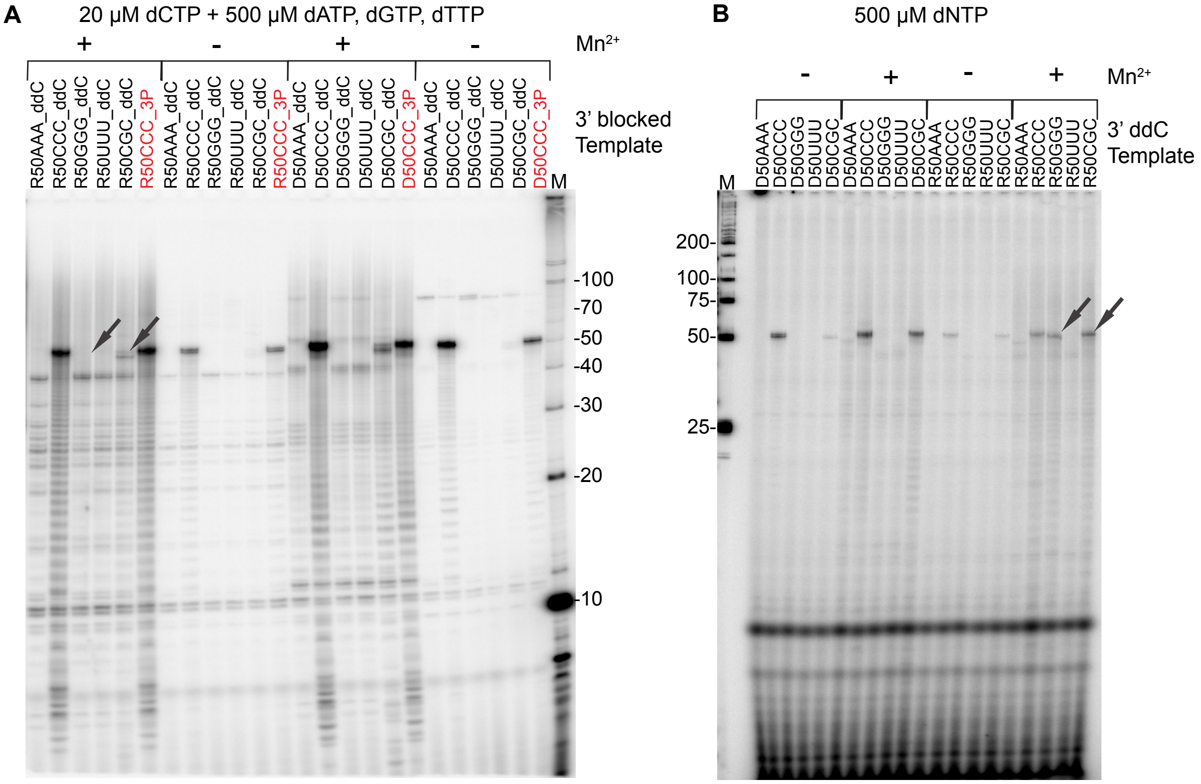

Fig. S17. Repeat experiments for fig. S5B.

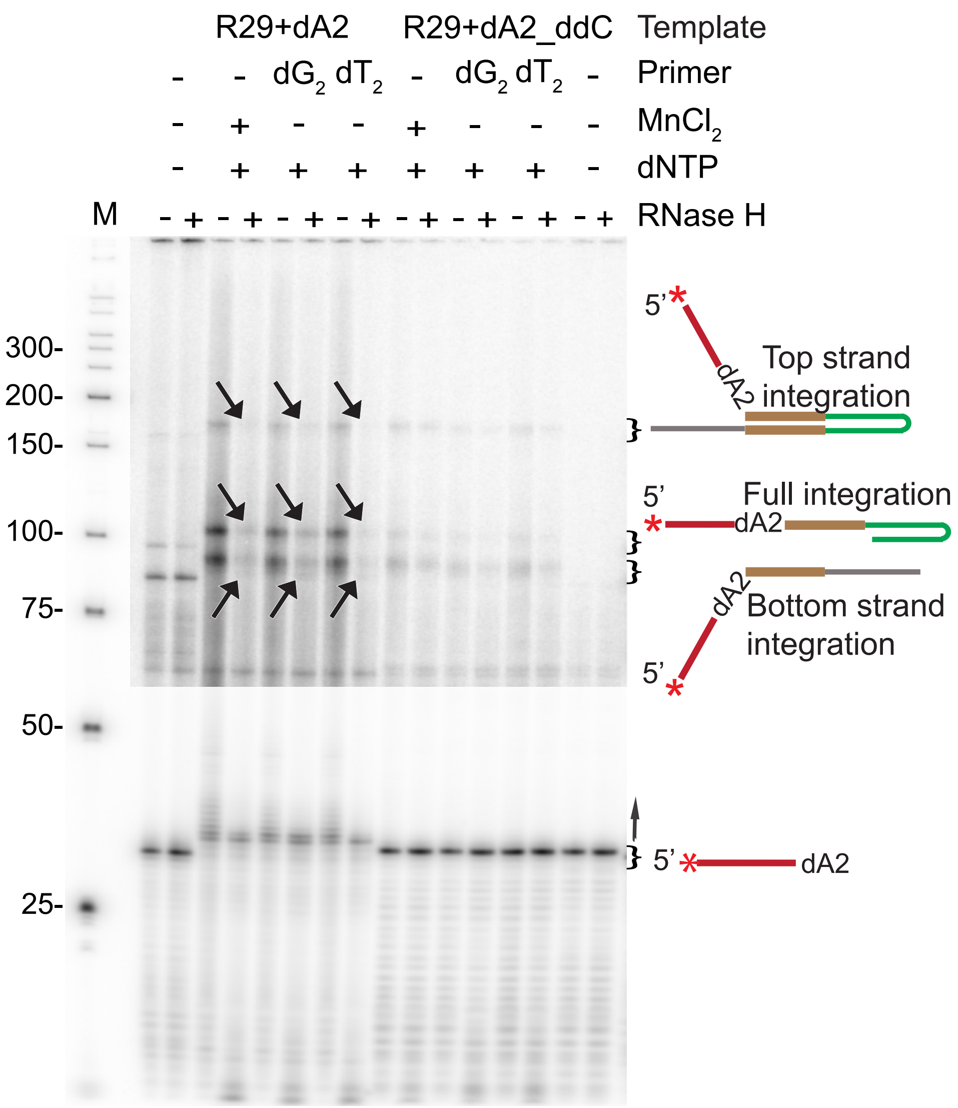

Table S1. List of oligonucleotides used. RNA sequences are shown in bold type, DNA sequences in regular type. N, equimolar dATP, dTTP, dGTP, dCTP; 3ddC, 3' dideoxy CTP; 3Phos, 3' phosphate; 3INVdT, 3' inverted dTTP; 5Phos, 5' phosphate; 3SpC3, 3' C3 Spacer.

Project	Name	Sequence
cDNA Templates	R50CCC	**GCAAUAAUCUAUACAAUACAACACAUACAAACAAAUUCUUAAGGUCCCAA**
	D50CCC	GCAATAATCTATACAATACAACACATACAAACAAATTCTTAAGGTCCCAA
	R50CCC_ddC	**GCAAUAAUCUAUACAAUACAACACAUACAAACAAAUUCUUAAGGUCCCAA**/3ddC
	D50CCC_ddC	GCAATAATCTATACAATACAACACATACAAACAAATTCTTAAGGTCCCAA/3ddC
	R50CCC_3P	**GCAAUAAUCUAUACAAUACAACACAUACAAACAAAUUCUUAAGGUCCCAA**/3Phos
	D50CCC_3P	GCAATAATCTATACAATACAACACATACAAACAAATTCTTAAGGTCCCAA/3Phos
	R50CCC_INVdT	**GCAAUAAUCUAUACAAUACAACACAUACAAACAAAUUCUUAAGGUCCCAA**/3INVdT
	D50CCC_INVdT	GCAATAATCTATACAATACAACACATACAAACAAATTCTTAAGGTCCCAA/3INVdT
	R50CGC_ddC	**GCAAUAAUCUAUACAAUACAACACAUACAAACAAAUUCUUAAGGUCGCAA**/3ddC
	D50CGC_ddC	GCAATAATCTATACAATACAACACATACAAACAAATTCTTAAGGTCGCAA/3ddC
	R50AAA_ddC	**GCAAUAAUCUAUACAAUACAACACAUACAAACAAAUUCUUAAGGUAAAAA**/3ddC
	D50AAA_ddC	GCAATAATCTATACAATACAACACATACAAACAAATTCTTAAGGTAAAAA/3ddC
	R50GGG_ddC	**GCAAUAAUCUAUACAAUACAACACAUACAAACAAAUUCUUAAGGUGGGAA**/3ddC
	D50GGG_ddC	GCAATAATCTATACAATACAACACATACAAACAAATTCTTAAGGTGGGAA/3ddC
	R50UUU_ddC	**GCAAUAAUCUAUACAAUACAACACAUACAAACAAAUUCUUAAGGUUUUAA**/3ddC
	D50TTT_ddC	GCAATAATCTATACAATACAACACATACAAACAAATTCTTAAGGTTTTAA/3ddC
	R29CCC+dA4_ddC	**CACAUACAAACAAAUUCUUAAGGUCCCAA**AAAA/3ddC
	R30CCC+dA4_ddC	**ACACAUACAAACAAAUUCUUAAGGUCCCAA**AAAA/3ddC
	R31CCC+dA4_ddC	**AACACAUACAAACAAAUUCUUAAGGUCCCAA**AAAA/3ddC
	R32CCC+dA4_ddC	**CAACACAUACAAACAAAUUCUUAAGGUCCCAA**AAAA/3ddC
	R31CCC+dA3_ddC	**AACACAUACAAACAAAUUCUUAAGGUCCCAA**AAA/3ddC
	R32CCC+dA4_ddC	**CAACACAUACAAACAAAUUCUUAAGGUCCCAA**AA/3ddC
	R30CCC+dA1ddC	**ACACAUACAAACAAAUUCUUAAGGUCCCAA**A/3ddC
	R30CCC+dA2ddC	**ACACAUACAAACAAAUUCUUAAGGUCCCAA**AA3ddC
	R30CCC+dA3ddC	**ACACAUACAAACAAAUUCUUAAGGUCCCAA**AAA/3ddC
	R30CCC+dA4ddC	**ACACAUACAAACAAAUUCUUAAGGUCCCAA**AAAA/3ddC
	R30CCC+dA5ddC	**ACACAUACAAACAAAUUCUUAAGGUCCCAA**AAAAA/3ddC
	R29+dA2_ddC	**UUUCUCGAGUCAUCUUUUAGGGCUCCAAG**AA/3ddC
	R29+dA3_ddC	**UUUCUCGAGUCAUCUUUUAGGGCUCCAAG**AAA/3ddC
	R29+dA4_ddC	**UUUCUCGAGUCAUCUUUUAGGGCUCCAAG**AAAA/3ddC
	R29+dA5_ddC	**UUUCUCGAGUCAUCUUUUAGGGCUCCAAG**AAAAA/3ddC
	R29+dA6_ddC	**UUUCUCGAGUCAUCUUUUAGGGCUCCAAG**AAAAAA/3ddC
Cloning	Cas2_pet_5	GATCCATATGATGAGAATATACCTTGCCTGTTT
	Cas2_pet_3	TTTGGATCCTTATCATAACAGGACGGCGG
Primers	dA_2	AA
	dC_2	CC
	dG_2	GG
	dT_2	TT
CRISPR DNA	MMB1Lead40-5	AGGTTAACCTGCTGAAATGATTGG
	MMB1crisp3_r1	GGAGATCTTTAAAGTCTCAACG
	L20R1top_3BIO	TTGGAAAAAATAAGGGTACTGTTTCAGACCCGCTGGCCGCTTAGGCCGTTGAGACTTTAAAGATCTCC/3BIOTIN
	L20R1bot_3Bio	GGAGATCTTTAAAGTCTCAACGGCCTAAGCGGCCAGCGGGTCTGAAACAGTACCCTTATTTTTTCCAA/3BIOTIN
	CRISPR-HP3blunt	TTGGAAAAAATAAGGGTACTGTTTCAGACCCGCTGGCCGCTTAGGCCGTTGAGACTTTAAAGATCTCCTACATGGAGATCTTTAAAGTCTCAACGGCCTAAGCGGCCAGCGGGTCTGAAACAGTACCCTTATTTTTTCCAA
CRISPR assay	R29	**UUUCUCGAGUCAUCUUUUAGGGCUCCAAG**
	R29+dA1	**UUUCUCGAGUCAUCUUUUAGGGCUCCAAG**A
	R29+dA2	**UUUCUCGAGUCAUCUUUUAGGGCUCCAAG**AA
	R29+dA3	**UUUCUCGAGUCAUCUUUUAGGGCUCCAAG**AAA
	R29+dA4	**UUUCUCGAGUCAUCUUUUAGGGCUCCAAG**AAAA
	R29+dA5	**UUUCUCGAGUCAUCUUUUAGGGCUCCAAG**AAAAA
	R29+dA6	**UUUCUCGAGUCAUCUUUUAGGGCUCCAAG**AAAAAA
	D29	TTTCTCGAGTCATCTTTTAGGGCTCCAAG
	D29+dA1	TTTCTCGAGTCATCTTTTAGGGCTCCAAGA
	D29+dA2	TTTCTCGAGTCATCTTTTAGGGCTCCAAGAA
	D29+dA3	TTTCTCGAGTCATCTTTTAGGGCTCCAAGAAA
	D29+dA4	TTTCTCGAGTCATCTTTTAGGGCTCCAAGAAAA
	D29+dA5	TTTCTCGAGTCATCTTTTAGGGCTCCAAGAAAAA
	D29+dA6	TTTCTCGAGTCATCTTTTAGGGCTCCAAGAAAAAA
	D29r+dA1	CTTGGAGCCCTAAAAGATGACTCGAGAAAA
	D29r+dA2	CTTGGAGCCCTAAAAGATGACTCGAGAAAAA
	R30CCC+dA4	**ACACAUACAAACAAAUUCUUAAGGUCCCAA**AAAA
	D30CCCr+dA1	TTGGGACCTTAAGAATTTGTTTGTATGTGTA
Terminal transferase	R29	**UUUCUCGAGUCAUCUUUUAGGGCUCCAAG**
	D29	TTTCTCGAGTCATCTTTTAGGGCTCCAAG
	R34	**AGCGUCCGUUCCAGACAUUCAGCCCUCUAGUAGA**
	D34	AGCGTCCGTTCCAGACATTCAGCCCTCTAGTAGA
Markers	dG_2	GG
	dG_3	GGG
TGIRT seq	R2 RNA	**AAGAUCGGAAGAGCACACGUCUGAACUCCAGUCAC/3SpC3**
	R2R DNA (N=equimolar dA, dT, dG, dC)	GTGACTGGAGTTCAGACGTGTGCTCTTCCGATCTTN
	R1R DNA	/5Phos/GATCGTCGGACTGTAGAACTCTGAACGTGTAG/3SpC3
	R1 DNA	CTACACGTTCAGAGTTCTACAGTCCGACGATC/3SpC3
	Illumina multiplex PCR primer	AATGATACGGCGACCACCGAGATCTACACGTTCAGAGTTCTACAGTCCGACGATC
	Illumina barcode PCR primer1	CAAGCAGAAGACGGCATACGAGATCGTGATGTGACTGGAGTTCAGACGTGTGCTCTTCCGATCT
	Illumina barcode PCR primer2	CAAGCAGAAGACGGCATACGAGATACATCGGTGACTGGAGTTCAGACGTGTGCTCTTCCGATCT
	Illumina barcode PCR primer3	CAAGCAGAAGACGGCATACGAGATGCCTAAGTGACTGGAGTTCAGACGTGTGCTCTTCCGATCT
	Illumina barcode PCR primer4	CAAGCAGAAGACGGCATACGAGATTGGTCAGTGACTGGAGTTCAGACGTGTGCTCTTCCGATCT
	Illumina barcode PCR primer5	CAAGCAGAAGACGGCATACGAGATCACTGTGTGACTGGAGTTCAGACGTGTGCTCTTCCGATCT
	Illumina barcode PCR primer6	CAAGCAGAAGACGGCATACGAGATATTGGCGTGACTGGAGTTCAGACGTGTGCTCTTCCGATCT
	Illumina barcode PCR primer7	CAAGCAGAAGACGGCATACGAGATGATCTGGTGACTGGAGTTCAGACGTGTGCTCTTCCGATCT
	Illumina barcode PCR primer8	CAAGCAGAAGACGGCATACGAGATTCAAGTGTGACTGGAGTTCAGACGTGTGCTCTTCCGATCT
	Illumina barcode PCR primer9	CAAGCAGAAGACGGCATACGAGATCTGATCGTGACTGGAGTTCAGACGTGTGCTCTTCCGATCT
	Illumina barcode PCR primer10	CAAGCAGAAGACGGCATACGAGATAAGCTAGTGACTGGAGTTCAGACGTGTGCTCTTCCGATCT
	Illumina barcode PCR primer11	CAAGCAGAAGACGGCATACGAGATGTAGCCGTGACTGGAGTTCAGACGTGTGCTCTTCCGATCT
	Illumina barcode PCR primer12	CAAGCAGAAGACGGCATACGAGATTACAAGGTGACTGGAGTTCAGACGTGTGCTCTTCCGATCT
	Illumina barcode PCR primer13	CAAGCAGAAGACGGCATACGAGATTTGACTGTGACTGGAGTTCAGACGTGTGCTCTTCCGATCT
	Illumina barcode PCR primer14	CAAGCAGAAGACGGCATACGAGATGGAACTGTGACTGGAGTTCAGACGTGTGCTCTTCCGATCT
	Illumina barcode PCR primer15	CAAGCAGAAGACGGCATACGAGATTGACATGTGACTGGAGTTCAGACGTGTGCTCTTCCGATCT
	Illumina barcode PCR primer16	CAAGCAGAAGACGGCATACGAGATGGACGGGTGACTGGAGTTCAGACGTGTGCTCTTCCGATCT
	Illumina barcode PCR primer17	CAAGCAGAAGACGGCATACGAGATCTCTACGTGACTGGAGTTCAGACGTGTGCTCTTCCGATCT
	Illumina barcode PCR primer18	CAAGCAGAAGACGGCATACGAGATGCGGACGTGACTGGAGTTCAGACGTGTGCTCTTCCGATCT
	Illumina barcode PCR primer19	CAAGCAGAAGACGGCATACGAGATTTTCACGTGACTGGAGTTCAGACGTGTGCTCTTCCGATCT
	Illumina barcode PCR primer20	CAAGCAGAAGACGGCATACGAGATGGCCACGTGACTGGAGTTCAGACGTGTGCTCTTCCGATCT
	Illumina barcode PCR primer21	CAAGCAGAAGACGGCATACGAGATCGAAACGTGACTGGAGTTCAGACGTGTGCTCTTCCGATCT
	Illumina barcode PCR primer22	CAAGCAGAAGACGGCATACGAGATCGTACGGTGACTGGAGTTCAGACGTGTGCTCTTCCGATCT
	Illumina barcode PCR primer23	CAAGCAGAAGACGGCATACGAGATCCACTCGTGACTGGAGTTCAGACGTGTGCTCTTCCGATCT
	Illumina barcode PCR primer24	CAAGCAGAAGACGGCATACGAGATATCAGTGTGACTGGAGTTCAGACGTGTGCTCTTCCGATCT
	Illumina barcode PCR primer25	CAAGCAGAAGACGGCATACGAGATGCTCATGTGACTGGAGTTCAGACGTGTGCTCTTCCGATCT
	Illumina barcode PCR primer26	CAAGCAGAAGACGGCATACGAGATAGGAATGTGACTGGAGTTCAGACGTGTGCTCTTCCGATCT
	Illumina barcode PCR primer27	CAAGCAGAAGACGGCATACGAGATCTTTTGGTGACTGGAGTTCAGACGTGTGCTCTTCCGATCT
	Illumina barcode PCR primer28	CAAGCAGAAGACGGCATACGAGATTAGTTGGTGACTGGAGTTCAGACGTGTGCTCTTCCGATCT
	Illumina barcode PCR primer29	CAAGCAGAAGACGGCATACGAGATCCGGTGGTGACTGGAGTTCAGACGTGTGCTCTTCCGATCT
	Illumina barcode PCR primer30	CAAGCAGAAGACGGCATACGAGATATCGTGGTGACTGGAGTTCAGACGTGTGCTCTTCCGATCT
	Illumina barcode PCR primer31	CAAGCAGAAGACGGCATACGAGATTGAGTGGTGACTGGAGTTCAGACGTGTGCTCTTCCGATCT
	Illumina barcode PCR primer32	CAAGCAGAAGACGGCATACGAGATCGCCTGGTGACTGGAGTTCAGACGTGTGCTCTTCCGATCT

Table S2. List of R^2 values for curve fits. Data are for experiments shown in Figures 2, 3C, 9B-D, S2, S4C, S6A-C and S7A-C.

Protospacer	Band	R^2 values		
		WT 5 nM protospacer	RTΔ 5 nM protospacer	WT 250 nM protospacer
*R29+dA4 ss	**154 nt**	0.9932	0.9865	0.8303
*R29+dA4 ss	**99 nt**	0.9983	0.9901	0.9993
*R29+dA4 ss	**88 nt**	0.9968	0.97	0.9676
*R29+dA4/D29r+dA2 ds	**154 nt**	0.9882	0.9255	0.8719
*R29+dA4/D29r+dA2 ds	**99 nt**	0.9929	0.9827	0.9972
*R29+dA4/D29r+dA2 ds	**88 nt**	0.9974	0.9357	0.9693
R29+dA4/*D29r+dA2 ds	**154 nt**	0.9727	0.9534	0.4491
R29+dA4/*D29r+dA2 ds	**99 nt**	0.9775	0.9595	0.9943
R29+dA4/*D29r+dA2 ds	**88 nt**	0.926	0.8398	0.8862
*D29r+dA2 ss	**154 nt**	0.9952	0.9679	0.8245
*D29r+dA2 ss	**99 nt**	0.9915	0.9721	0.9908
*D29r+dA2 ss	**88 nt**	0.9863	0.9393	0.9476
*D29+dA4 ss	**154 nt**	0.9984	0.9759	
*D29+dA4 ss	**99 nt**	0.9993	0.9712	
*D29+dA4 ss	**88 nt**	0.9983	0.9408	
*D29+dA4/D29r+dA2 ds	**154 nt**	0.9485	0.9013	
*D29+dA4/D29r+dA2 ds	**99 nt**	0.9726	0.9213	
*D29+dA4/D29r+dA2 ds	**88 nt**	0.989	0.7121	
D29+dA4/*D29r+dA2 ds	**154 nt**	0.9924	0.9633	
D29+dA4/*D29r+dA2 ds	**99 nt**	0.9853	0.9754	
D29+dA4/*D29r+dA2 ds	**88 nt**	0.9896	0.7568	
*R29+dA5 ss	**154 nt**	0.9681	0.9884	0.9306
*R29+dA5 ss	**99 nt**	0.9994	0.9661	0.9972
*R29+dA5 ss	**88 nt**	0.9981	0.974	0.9323
*R29+dA5/D29r+dA1 ds	**154 nt**	0.9439	0.9705	0.567
*R29+dA5/D29r+dA1 ds	**99 nt**	0.9893	0.9807	0.9939
*R29+dA5/D29r+dA1 ds	**88 nt**	0.9954	0.9695	0.9628
R29+dA5/*D29r+dA1 ds	**154 nt**	0.9908	0.9879	0.9125
R29+dA5/*D29r+dA1 ds	**99 nt**	0.9862	0.9946	0.9981
R29+dA5/*D29r+dA1 ds	**88 nt**	0.9766	0.9635	0.9778
*D29r+dA1 ss	**154 nt**	0.9959	0.9641	0.7696
*D29r+dA1 ss	**99 nt**	0.9794	0.9549	0.9964
*D29r+dA1 ss	**88 nt**	0.9934	0.9329	0.9346
*D29+dA5 ss	**154 nt**	0.9978	0.9413	
*D29+dA5 ss	**99 nt**	0.9961	0.972	
*D29+dA5 ss	**88 nt**	0.9981	0.9169	
*D29+dA5/D29r+dA1 ds	**154 nt**	0.8823	0.937	
*D29+dA5/D29r+dA1 ds	**99 nt**	0.9801	0.9854	
*D29+dA5/D29r+dA1 ds	**88 nt**	0.957	0.9118	
D29+dA5/*D29r+dA1 ds	**154 nt**	0.9781	0.9513	
D29+dA5/*D29r+dA1 ds	**99 nt**	0.9968	0.99	
D29+dA5/*D29r+dA1 ds	**88 nt**	0.995	0.9569	
*R30CCC+dA4 ss	**154 nt**	0.9507	0.9585	0.7574
*R30CCC+dA4 ss	**99 nt**	0.9885	0.9649	0.9938
*R30CCC+dA4 ss	**88 nt**	0.9974	0.9406	0.8893
*R30CCC+dA4/D30CCCr+dA1 ds	**154 nt**	0.9974	0.9385	0.5661
*R30CCC+dA4/D30CCCr+dA1 ds	**99 nt**	0.999	0.9826	0.9856
*R30CCC+dA4/D30CCCr+dA1 ds	**88 nt**	0.9976	0.9686	0.928
R30CCC+dA4/*D30CCCr+dA1 ds	**154 nt**	0.9614	0.8651	0.8928
R30CCC+dA4/*D30CCCr+dA1 ds	**99 nt**	0.9949	0.9763	0.9407
R30CCC+dA4/*D30CCCr+dA1 ds	**88 nt**	0.8734	0.8389	0.8487
*D30CCCr+dA1 ss	**154 nt**	0.928	0.8795	0.5414
*D30CCCr+dA1 ss	**99 nt**	0.9976	0.941	0.9415
*D30CCCr+dA1 ss	**88 nt**	0.9328	0.8814	0.8747
*D30CCC+dA4 ss	**154 nt**	0.9948	0.9655	
*D30CCC+dA4 ss	**99 nt**	0.9944	0.9742	
*D30CCC+dA4 ss	**88 nt**	0.9917	0.9289	
*D30CCC+dA4/D30CCCr+dA1 ds	**154 nt**	0.9946	0.9598	
*D30CCC+dA4/D30CCCr+dA1 ds	**99 nt**	0.9966	0.982	
*D30CCC+dA4/D30CCCr+dA1 ds	**88 nt**	0.9991	0.9555	
D30CCC+dA4/*D30CCCr+dA1 ds	**154 nt**	0.9891	0.8706	
D30CCC+dA4/*D30CCCr+dA1 ds	**99 nt**	0.9828	0.9903	
D30CCC+dA4/*D30CCCr+dA1 ds	**88 nt**	0.9802	0.8806	
*D30CCCr+dA1 ss	**154 nt**	0.9732	0.9246	
*D30CCCr+dA1 ss	**99 nt**	0.9979	0.9557	
*D30CCCr+dA1 ss	**88 nt**	0.9688	0.9009	

Protospacer		R^2 values	
		Fig. 2C	Fig. S2
*R29+dA2		0.9663	0.9652
*R29+dA3		0.9614	0.9720
*R29+dA4		0.9742	0.9743
*R29+dA5		0.9807	0.9851
*R29+dA6		0.960	0.9537
*D29r+dA2		0.9595	0.9769
*D29r+dA3		0.9886	0.9658
*D29r+dA4		0.9981	0.9904
*D29r+dA5		0.9004	0.9876
*D29r+dA6		0.9885	0.9761
	**dNTP**	**Fig. 2**	
*R29	dA	0.9501	
*R29	dA + Mn^{2+}	0.9990	
*R29	dC + Mn^{2+}	0.9033	
*R29	dG + Mn^{2+}	0.9858	
*D29	dA	0.9953	
*D29	dA + Mn^{2+}	0.9954	
*D29	dC^{+}	0.9913	
*D29	dC + Mn^{2+}	0.9821	
*D29	dG	0.9131	
*D29	dG + Mn^{2+}	0.9704	
*D29	dT	0.8459	
*D29	dT + Mn^{2+}	0.9050	
	**Primer**	**Fig. S4C**	
R50CCC_ddC	None	0.9992	
R50CCC_ddC	None + Mn^{2+}	0.9990	
R50CCC_ddC	dG$_2$	0.9983	
R50CCC_ddC	dG$_2$ + Mn^{2+}	0.9938	

Table S3. Datasets. RNA template sequences are shown in bold type, DNA template sequences in regular type. 3ddC, 3’ dideoxy CTP. TGIRT-seq dataset obtained in this study were deposited in the Sequence Read Archive (SRA) with the accession number PRJNA1003443.

Dataset	Template name	Primer	Mn^{2+}	RNase A	Template oligo sequence	Accession
Dataset1	R29+dA3_ddC	-	-	-	**UUUCUCGAGUCAUCUUUUAGGGCUCCAAGAA**AAA/3ddC	SRR25570276
Dataset2	R29+dA4_ddC	-	-	-	**UUUCUCGAGUCAUCUUUUAGGGCUCCAAGAA**AAAA/3ddC	SRR25570275
Dataset3	R29+dA6_ddC	-	-	-	**UUUCUCGAGUCAUCUUUUAGGGCUCCAAGAA**AAAAAA/3ddC	SRR25570264
Dataset4	R29+dA3_ddC	-	+	-	**UUUCUCGAGUCAUCUUUUAGGGCUCCAAGAA**AAA/3ddC	SRR25570253
Dataset5	R29+dA4_ddC	-	+	-	**UUUCUCGAGUCAUCUUUUAGGGCUCCAAGAA**AAAA/3ddC	SRR25570245
Dataset6	R29+dA6_ddC	-	+	-	**UUUCUCGAGUCAUCUUUUAGGGCUCCAAGAA**AAAAAA/3ddC	SRR25570244
Dataset7	R50AAA_ddC	-	-	+	**GCAAUAAUCUAUACAAUACAACACAUACAAACAAAUUCUUAAGGUAAAAA**/3ddC	SRR25570243
Dataset8	R50CCC_ddC	-	-	+	**GCAAUAAUCUAUACAAUACAACACAUACAAACAAAUUCUUAAGGUCCCAA**/3ddC	SRR25570242
Dataset9	R50GGG_ddC	-	-	+	**GCAAUAAUCUAUACAAUACAACACAUACAAACAAAUUCUUAAGGUGGGAA**/3ddC	SRR25570241
Dataset10	R50UUU_ddC	-	-	+	**GCAAUAAUCUAUACAAUACAACACAUACAAACAAAUUCUUAAGGUUUUAA**/3ddC	SRR25570240
Dataset11	R50CGC_ddC	-	-	+	**GCAAUAAUCUAUACAAUACAACACAUACAAACAAAUUCUUAAGGUCGCAA**/3ddC	SRR25570274
Dataset12	R50AAA_ddC	-	+	+	**GCAAUAAUCUAUACAAUACAACACAUACAAACAAAUUCUUAAGGUAAAAA**/3ddC	SRR25570273
Dataset13	R50CCC_ddC	-	+	+	**GCAAUAAUCUAUACAAUACAACACAUACAAACAAAUUCUUAAGGUCCCAA**/3ddC	SRR25570272
Dataset14	R50GGG_ddC	-	+	+	**GCAAUAAUCUAUACAAUACAACACAUACAAACAAAUUCUUAAGGUGGGAA**/3ddC	SRR25570271
Dataset15	R50UUU_ddC	-	+	+	**GCAAUAAUCUAUACAAUACAACACAUACAAACAAAUUCUUAAGGUUUUAA**/3ddC	SRR25570270
Dataset16	R50CGC_ddC	-	+	+	**GCAAUAAUCUAUACAAUACAACACAUACAAACAAAUUCUUAAGGUCGCAA**/3ddC	SRR25570269
Dataset17	R50AAA_ddC	dT_2	-	+	**GCAAUAAUCUAUACAAUACAACACAUACAAACAAAUUCUUAAGGUAAAAA**/3ddC	SRR25570268
Dataset18	R50CCC_ddC	dG_2	-	+	**GCAAUAAUCUAUACAAUACAACACAUACAAACAAAUUCUUAAGGUCCCAA**/3ddC	SRR25570267
Dataset19	R50GGG_ddC	dC_2	-	+	**GCAAUAAUCUAUACAAUACAACACAUACAAACAAAUUCUUAAGGUGGGAA**/3ddC	SRR25570266
Dataset20	R50UUU_ddC	dA_2	-	+	**GCAAUAAUCUAUACAAUACAACACAUACAAACAAAUUCUUAAGGUUUUAA**/3ddC	SRR25570265
Dataset21	R50CGC_ddC	dT_2	-	+	**GCAAUAAUCUAUACAAUACAACACAUACAAACAAAUUCUUAAGGUCGCAA**/3ddC	SRR25570263
Dataset22	R50CCC	-	-	-	**GCAAUAAUCUAUACAAUACAACACAUACAAACAAAUUCUUAAGGUCCCAA**	SRR25570262
Dataset23	R50CCC	-	-	+	**GCAAUAAUCUAUACAAUACAACACAUACAAACAAAUUCUUAAGGUCCCAA**	SRR25570261
Dataset24	R50CCC	-	+	-	**GCAAUAAUCUAUACAAUACAACACAUACAAACAAAUUCUUAAGGUCCCAA**	SRR25570260
Dataset25	R50CCC	-	+	+	**GCAAUAAUCUAUACAAUACAACACAUACAAACAAAUUCUUAAGGUCCCAA**	SRR25570259
Dataset26	R29CCC+dA4_ddC	-	-	+	**AACACAUACAAACAAAUUCUUAAGGUCCCAA**AAAA/3ddC	SRR25570258
Dataset27	R30CCC+dA4_ddC	-	-	+	**CAACACAUACAAACAAAUUCUUAAGGUCCCAA**AAAA/3ddC	SRR25570257
Dataset28	R31CCC+dA4_ddC	-	-	+	**ACAACACAUACAAACAAAUUCUUAAGGUCCCAA**AAAA/3ddC	SRR25570256
Dataset29	R32CCC+dA4_ddC	-	-	+	**UACAACACAUACAAACAAAUUCUUAAGGUCCCAA**AAAA/3ddC	SRR25570255
Dataset30	R31CCC+dA3_ddC	-	-	+	**ACAACACAUACAAACAAAUUCUUAAGGUCCCAA**AAA/3ddC	SRR25570254
Dataset31	R32CCC+dA2_ddC	-	-	+	**UACAACACAUACAAACAAAUUCUUAAGGUCCCAA**AA/3ddC	SRR25570252
Dataset32	R29CCC+dA4_ddC	-	+	+	**AACACAUACAAACAAAUUCUUAAGGUCCCAA**AAAA/3ddC	SRR25570251
Dataset33	R30CCC+dA4_ddC	-	+	+	**CAACACAUACAAACAAAUUCUUAAGGUCCCAA**AAAA/3ddC	SRR25570250
Dataset34	R31CCC+dA4d_dC	-	+	+	**ACAACACAUACAAACAAAUUCUUAAGGUCCCAA**AAAA/3ddC	SRR25570249
Dataset35	R32CCC+dA4_ddC	-	+	+	**UACAACACAUACAAACAAAUUCUUAAGGUCCCAA**AAAA/3ddC	SRR25570248
Dataset36	R31CCC+dA3_ddC	-	+	+	**ACAACACAUACAAACAAAUUCUUAAGGUCCCAA**AAA/3ddC	SRR25570247
Dataset37	R32CCC+dA2_ddC	-	+	+	**UACAACACAUACAAACAAAUUCUUAAGGUCCCAA**AA/3ddC	SRR25570246

Table S4. Data for Fig. 1B.

	MmRT-Cas1		FsRT-Cas1	VvRT-Cas1	Tt-Cas1	Se-Cas1	St-Cas1
Cas system	RT-Cas1	RTΔ-Cas1	RT-Cas1	RT-Cas1	Cas1	Cas1	Cas1
Origin organism	Marinomonas mediterranea		Fusicatenibacter saccharivorans	Vibrio vulnificus	Thermus thermophilus	Staphylococcus epidermidis	Streptococcus thermophilus
Assay organism	Marinomonas mediterranea		Escherichia coli	Escherichia coli	Thermus thermophilus	Staphylococcus aureus	Streptococcus thermophilus
Assay organism strain	MMB-1		BL21-Gold(DE3) pLysS AG	HMS174(DE3)	HB27c	RN4220	JIM 8232
Target genome	MMB-1		BL21-Gold(DE3) pLysS AG	HMS174(DE3)	Phage phiFa/Ko	RN4220	JIM 8232
Target genome ID	NC_015276.1		NC_012947.1	NZ_LM993812.1	MH673671.2, MH673672.2	NZ_CP076105.1	FR875178.1
BioProject	PRJNA301768		PRJNA484149	PRJNA539885	PRJNA631468	PRJNA769698	PRJNA762861
BioSample	SRR2913703-810	SRR2913815-818	SRR8102160, 163, 166-8, 170, 172-7, 194, 207	SRR8962131-134,137,138	SRR11818505-8,SRR12227011-4	SRR16249482-3	SRR1595171-3, 176,187,198,209
Spacers (unique)	15,498	7,678	22,245	356	6,116	67,607	178,486
None	4,809	5,192	6,464	295	1,208	63,824	131,867
5'and3' ends	279	117	2,693	4	813	130	2,967
5' end only	4,933	1,255	5,509	15	2,771	1,861	21,912
3' end only	5,477	1,114	7,579	42	1,324	1,792	21,740
Spacers with soft clip (only in one end, 5' or 3', not both)	10,410	2,369	13,088	57	4,095	3,653	43,652
1nt	3,089	1,075	1,787	18	3,388	2,798	20,221
≥2nt	7,321	1,294	11,301	39	707	855	23,431
